# Outcomes reported in interventional studies for mild cognitive impairment: a scoping umbrella review to inform core outcome set development

**DOI:** 10.1002/alz.71800

**Published:** 2026-09-05

**Authors:** Victoria Grace Gabb, Sam Harding, Tomas Lemke, Sophie Alderman, Natalie Woodward, Julie Clayton, Angus McNair, Winsome Barrett‐Muir, Alan Richardson, Jemima Dooley, Sarah Rudd, Elizabeth Coulthard, Nicholas Turner

**Affiliations:** ^1^ University of Bristol Bristol UK; ^2^ North Bristol NHS Trust Bristol UK; ^3^ NIHR Bristol Biomedical Research Centre Bristol UK; ^4^ University of Bath Bath UK; ^5^ ReMemBr Lived Experience Expert Group Bristol UK; ^6^ University of Exeter Exeter UK

**Keywords:** Alzheimer's disease, clinical trials, core outcome set (COS), mild cognitive impairment (MCI), outcome selection

## Abstract

There is currently no consensus on the most important treatment outcomes in patients with mild cognitive impairment (MCI). As part of developing a core outcome set for MCI, we conducted a pragmatic scoping umbrella review to summarize outcomes reported in previous studies. Six electronic databases were searched from inception to August 2023. Systematic and scoping reviews in English with ≥70% of studies describing at least one quantitative outcome of interventions involving adults with MCI were eligible. Verbatim outcomes were extracted from eligible papers within selected reviews. From 170 papers covering 153 studies from 42 reviews, 143 unique outcomes were identified. Global cognition was the only outcome reported by more than 50% of studies. Two‐thirds of outcomes were reported by no more than three studies. Heterogeneous outcome reporting limits evidence synthesis and treatment evaluation. A core outcome set for MCI is warranted to standardize outcome reporting and offer stronger evidence for decision‐making.

## Highlights

- MCI describes cognitive decline beyond that expected for age, but without significant impact on daily functioning.
- There is currently no consensus on what outcomes should be prioritized in clinical trials or routine care for MCI.
- This scoping umbrella review highlights the breadth of outcomes and outcome measures used in interventional trials for MCI, with a focus on cognition.
- These findings will support the development of a core outcome set for MCI to improve outcome reporting and trial design.

## 1 BACKGROUND

Mild cognitive impairment (MCI) describes a clinical syndrome where there is a decline in cognitive function worse than expected for age, but without significant impact on daily functioning.^1^ MCI is often the earliest symptomatic stage in neurodegenerative diseases, such as Alzheimer's disease (AD), preceding dementia onset.^2^ Around 39% of patients with MCI progress to dementia in cohort studies longer than 5 years, with an annual conversion rate of approximately 10%.^3^

MCI is etiologically and prognostically heterogeneous.^4^ It is often classified into subtypes depending on whether memory is affected (amnestic or non‐amnestic) and how many cognitive domains are affected (single‐domain or multi‐domain).^5^ The speed of decline can vary, and some patients may have stable MCI or revert to pre‐morbid cognition with treatment.^6^ It is also common for patients with MCI to present with multiple risk factors, co‐morbidities, and a complex neuropathological profile, all of which complicate diagnosis and treatment.^7^ As access to biomarker testing varies globally but is typically limited to specialist clinics or research,^8^ often the underlying cause and, therefore, likely trajectory of MCI remain unknown for both patients and their healthcare team.^9^

There is currently no standardized treatment pathway following a diagnosis of MCI.^10^, ^11^ Patients with MCI often have limited or no follow‐up until progression to dementia is suspected,^12^ or they may be offered non‐pharmacological treatments or symptomatic treatments off label.^13^, ^14^ Recent progress in anti‐amyloid therapies is promising for MCI due to AD; however, these treatments are currently expensive, not widely available, and are only modestly beneficial.^15^, ^16^ Moreover, many patients presenting to memory clinics will not be eligible for these treatments, with contraindications including other suspected causes of MCI, cerebrovascular disease, and APOE4 status.^11^, ^17^, ^18^ Therefore, MCI will likely need a multi‐therapeutic approach, and more research is needed to identify effective treatments.^19^

To develop better care for people living with MCI, it is crucial to understand what outcomes need to be improved. Choices of outcome (what is measured) and outcome measure (how it is measured) in interventional trials are critically important, as these determine whether a treatment can be considered effective.^20^ However, at present, consensus regarding which outcomes are most important for individuals living with MCI is lacking.^21^

A core outcome set (COS) is a minimum, standardized list of outcomes that should be measured and reported by clinical studies for a given condition.^22^ Developed through consensus with stakeholders including patients, families, healthcare professionals, and researchers, COS help to ensure that the most meaningful outcomes across stakeholder groups are consistently included, promote transparent outcome reporting, and enable synthesis across research studies.^22^, ^23^ COS development typically involves a literature review to identify outcomes reported in published studies, alongside qualitative interviews to identify additional outcomes that are important to stakeholders, followed by a Delphi study to prioritize important outcomes for the COS and a consensus meeting of stakeholders.^24^

This systematic scoping umbrella review forms the first part of a program of work to develop a COS for MCI.^21^ The aim of this review is to generate a list of outcomes that have been reported in interventional trials in participants with MCI. A secondary aim was to identify which outcomes and outcome measures have been most frequently reported.

## 2 METHODS

Given the volume of relevant trials identified in preliminary database searches, a scoping umbrella review of interventional studies in adults with MCI was undertaken. Scoping umbrella reviews are increasingly utilized in health research^25^, ^26^ and bring together the strengths of both scoping and umbrella reviews. Umbrella reviews integrate data from multiple reviews and enable research questions to be answered where a substantial number of systematic reviews are already available related to the area of interest.^27^ As the scope of the research question is broad and methodological, the scoping umbrella review approach is most suitable.^28^ The review was performed in accordance with the Joanna Briggs Institute (JBI) methodology for umbrella reviews.^29^ The review protocol was registered in the PROSPERO database (CRD42023452514). The full study protocol was published online^21^ and has been registered with the Core Outcome Measures in Effectiveness Trials (COMET) database (No. 2117).

### 2.1 Eligibility criteria

In line with the JBI guidance, the eligibility for inclusion in the review was defined using the Population, Concept, Context (PCC) framework.^29^ As this is an umbrella review, the only papers retained for inclusion were peer‐reviewed published reviews. Systematic or scoping reviews with at least 70% of studies meeting the criteria summarized below were eligible for inclusion (Table 1).

**TABLE 1:** Eligibility criteria for reviews and records in umbrella review.

Inclusion criteria	Exclusion criteria
**Population**: Adults aged 18 years or over, diagnosed with mild cognitive impairment (MCI) according to standardized research or clinical criteria **Concept**: Interventional trials reporting ≥1 quantitative outcome **Context**: Available in English language^*^, community‐dwelling	**Population**: Diagnosis not explicitly meeting standard or clinical diagnostic criteria for MCI (e.g., subjective memory complaints); mixed cohort (MCI and non‐MCI) where outcomes are not available separately for individuals with MCI **Concept**: Observational study, reporting qualitative outcomes **Context**: English translation unavailable, care home residents

* Primary articles not available in English were subtracted from the denominator used to calculate “≥ 70% included studies to be eligible” requirement to reduce bias against interventions predominantly conducted in non‐English‐speaking countries.

#### 2.1.1 Population

To be eligible for inclusion, reviews needed to include community‐dwelling adults (aged 18 or over) diagnosed with MCI according to standardized research criteria or clinical diagnosis. Where mixed cohorts (e.g., MCI and dementia) were included, outcomes for participants with MCI needed to be reported separately.

#### 2.1.2 Concept

We included scoping and systematic reviews reporting quantitative outcomes from interventional trials. Observational studies or studies including only qualitative outcomes were ineligible.

#### 2.1.3 Context

Reviews had to be published in English and focus on interventions that took place in the community (i.e., not in care homes). There was no restriction on geographical location.

### 2.2 Search strategy

The search strategies were developed by an experienced medical librarian (SR) in collaboration with the research team. Searches were conducted in Embase (Ovid), CINAHL (EBSCOhost), PsycINFO (Proquest), Medline (Ovid), PROSPERO, and COMET from inception to August 7, 2023. Free text terms and subject headings were used for MCI and evidence reviews (Appendix A). Search results were limited to human studies, and a language filter was applied to include only papers published in English. Medline records were removed from the Embase search to avoid duplication. Results were de‐duplicated using a reference management software package (EndNote 21.5) before being uploaded to Rayyan.^30^ Each citation reviewed at title, abstract, and full‐text screening was screened by at least two independent reviewers, with disagreements resolved via discussion or via the decision of a third reviewer.

**RESEARCH IN CONTEXT**

1. **Systematic review**: Despite several core outcome sets established for dementia, the trial outcomes most relevant to MCI are not yet established. Several publications describe challenges around outcome selection and how meaningful outcomes to patients may not reflect outcomes chosen in trials.
2. **Interpretation**: Our review highlights the breadth of outcomes used to assess interventions in MCI and is consistent with the need for a core outcome set in MCI.
3. **Future directions**: Outcomes reported in publications may reflect the interests of academics, funders, and regulatory agencies. Interviews with patients and relatives, as well as healthcare professionals and researchers, may identify further outcomes of importance and provide context. Integrating perspectives from different stakeholder groups will be needed to ensure the core outcome set is meaningful, pragmatic, and useful.

### 2.3 Data extraction and analysis

Verbatim outcome extraction from the source manuscripts is recommended for reviews as part of COS development.^24^ Thus, this review was initially designed as a modified umbrella review, with planned extraction of verbatim outcome data from all eligible primary studies included in all eligible systematic and scoping reviews.^21^ The number of eligible reviews was substantially higher than anticipated and full‐text screening suggested considerable overlap and saturation in both the primary studies and outcomes identified. There is a lack of guidance on how to manage unexpectedly large reviews as any protocol changes may introduce bias or not fully address the research question^31^, ^32^. Therefore, a stratified sampling approach based on intervention type was used. Data were extracted from all eligible primary studies of all reviews within an intervention type with fewer than five eligible reviews. For intervention types represented by five or more reviews, 50% of reviews were selected at random. Data was extracted from the full texts of eligible primary studies within these selected reviews. Further information is provided in Appendix B.

Data from the retained reviews was extracted using a researcher‐developed data extraction form. Study characteristics and verbatim outcomes and outcome measurement tools were extracted independently and checked for accuracy by another author. The wording of verbatim outcomes was reviewed and standardized to generate a list of unique outcomes. Outcomes were then categorized according to the core areas and outcome domains identified by Dodd's taxonomy for outcomes in medical research.^33^

We adopted the current working definition of a unique outcome as “one that has original meaning and context. Outcomes with different words, phrasing, or spelling addressing the same concept and context should be categorized as one outcome” and we did not consider outcomes measured at different timepoints as unique.^34^ Where it was unclear how granular the unique outcome should be, a pragmatic and iterative approach was adopted involving discussions within the research team and people with lived experience of MCI to determine whether including the more granular outcome was specifically meaningful in MCI.

The number and percentage of studies reporting each unique outcome is reported, alongside verbatim examples taken from papers. The wording of verbatim outcome measurement tools was reviewed and standardized and the number of times a tool was used is reported. Where studies used a composite (e.g., Z‐score of the sum of multiple instruments) or only part of a validated test without also reporting values for the full standalone assessment, this adaptation was described instead.

### 2.4 Patient and public involvement

Patient and public involvement (PPI) enhances the real‐world relevance and impact of research and is increasingly incorporated into systematic reviews.^35^ Two PPI co‐investigators with lived experience of MCI and dementia (WBM, AR) were integral to the study management group, which met regularly throughout the project to oversee study progress and quality. Additional PPI contributors with lived experience of MCI and dementia as patients, partners, and relatives helped to design the study. The PPI group were consulted on decisions on whether outcomes could be considered unique and outcome granularity. PPI contributors were not involved in the review process or reporting.

## 3 RESULTS

**FIGURE 1:**
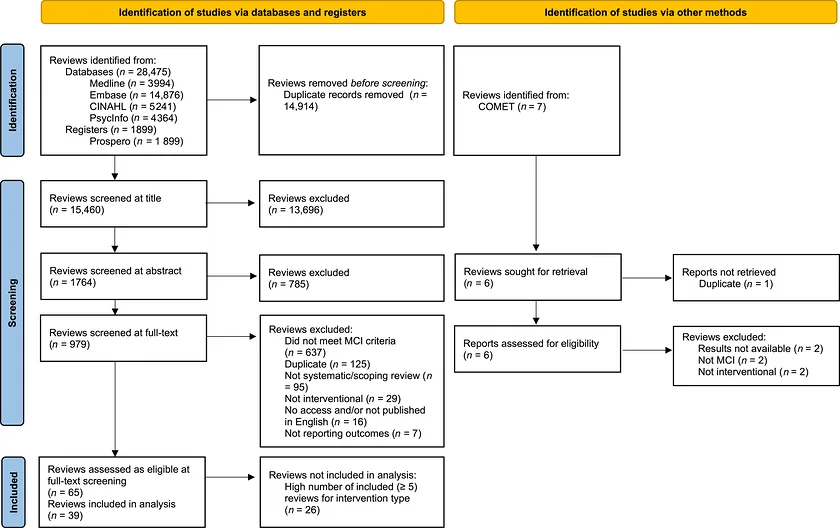
PRISMA flowchart, adapted from PRISMA 2020 flowchart by Page et al. (2021), https://doi.org/10.1371/journal.pmed.1003583.

The search resulted in a total of 30,374 records, of which 15,460 remained after duplicates were removed (Figure 1). After assessing titles and abstracts, 979 records were selected for full‐text screening, of which 65 records were considered eligible. Following stratification by intervention type, 39 reviews were included in the analysis, and data were extracted from 154 primary studies taken from 174 papers (Appendix B). Each review added a mean of 1.9 (range: 0 to 6) unique studies not included by another review. Approximately half of the studies (*N* = 74, 48%) were included by more than two reviews, mean (standard deviation [SD]) = 2.3 (1.8), range = 1 to 11.

### 3.1 Characteristics of included reviews

Thirty‐nine reviews were included covering the following intervention types: cognitive interventions (*N* = 10), traditional Chinese medicine (*N* = 7), non‐pharmacological interventions (*N* = 4), multidomain interventions (*N* = 4), exercise (*N* = 4), computer‐based cognitive training (*N* = 3), dance (*N* = 2), non‐invasive brain stimulation (*N* = 2), pharmacological (*N* = 2), and not specified (*N* = 1). Seven eligible reviews contained eligible articles but no unique studies not included in another review, suggesting saturation of relevant studies. Additional information on the reviews included is provided in Appendix B.

### 3.2 Characteristics of included studies

**FIGURE 2:**
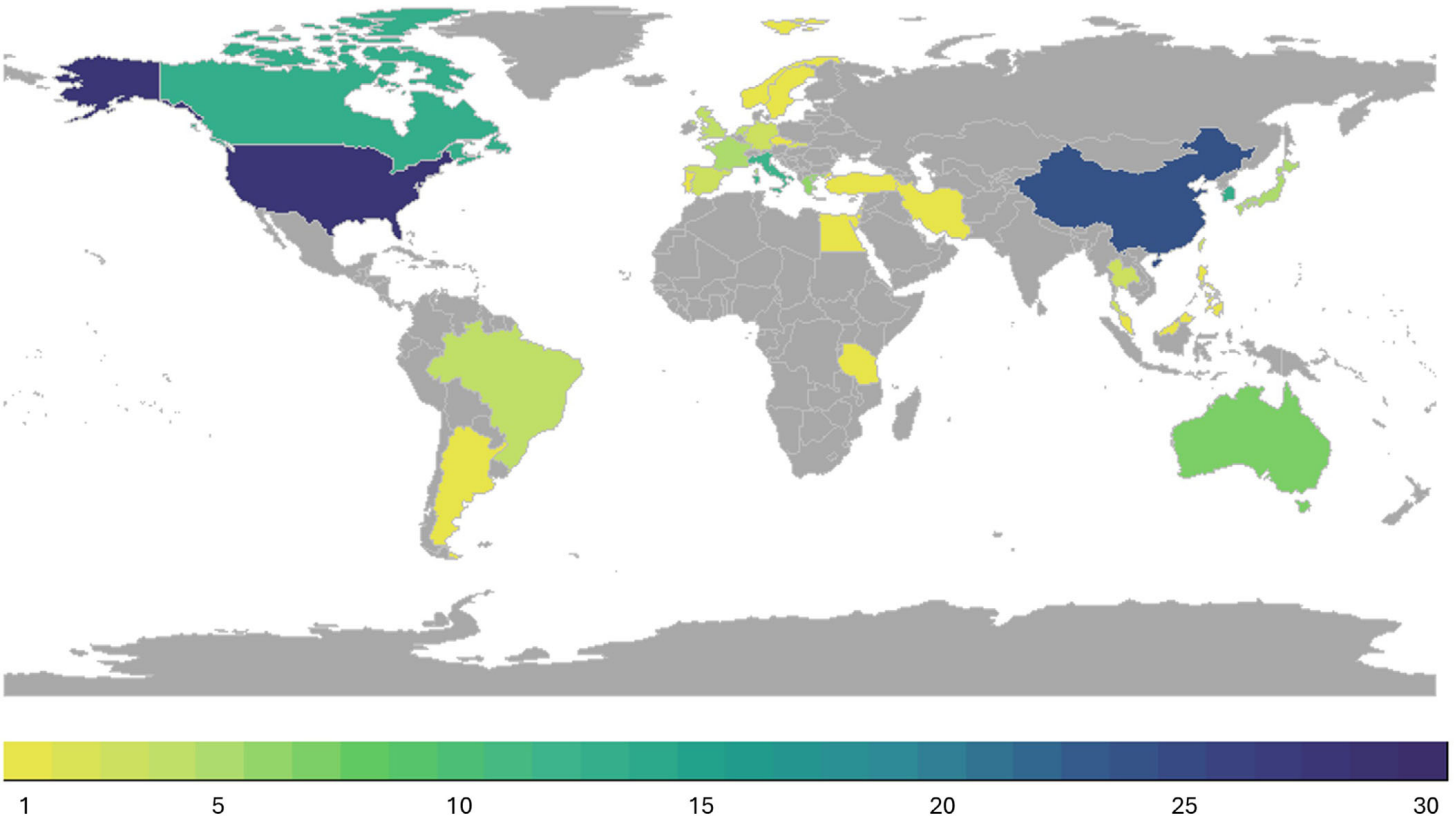
Geographical distribution of included studies.

**FIGURE 3 alz71800-fig-0003:**
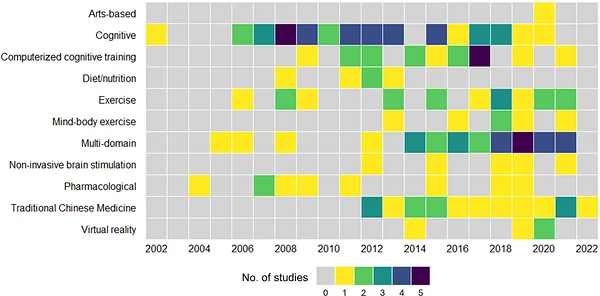
Heatmap of intervention types over time for included studies.

Study characteristics for included primary articles are provided in Appendix C. The analysis included studies published in 31 countries across six continents, with the highest proportion of studies undertaken in the United States (*N* = 26, 17%) and China (*N* = 24, 16%) (Figure 2). The most common intervention type was cognitive (*N* = 42, 27%), followed by multi‐domain interventions (*N* = 31, 20%) (Figure 3). Most studies were randomized controlled trials (*N* = 126, 82%) and included participants with MCI not otherwise specified (*N* = 89, 58%) or amnestic MCI (*N* = 61, 40%). Most studies used the Petersen criteria^36^, ^37^ for diagnosing or confirming MCI (*N* = 90, 58%). Studies were published between 2002 and 2022. Studies excluded from eligible reviews were predominantly due to not being available in English or not meeting defined MCI population criteria (Appendix C).

In total, 14,064 participants were included in the analysis. Of the 142 studies reporting sex, 57% (*N* = 7848) were female and 43% (*N* = 5859) were male. Three studies recruited only women.^38^, ^39^, ^40^ Of the 144 studies reporting or allowing a calculation of mean age, participants were aged 71.0 years (SD = 4.2) on average. Race, ethnicity, or nationality information was scarcely reported, though notable exceptions include a behavioral activation study focused on Black individuals^41^ and a virtual reality intervention based on Korean culture.^42^ Similarly, socioeconomic status or educational attainment of participants was typically not reported, though one study tested the effectiveness of visual art therapy and specifically recruited illiterate older adults in a Tanzanian village.^43^

### 3.3 Main findings

Five hundred fifty‐two outcomes were extracted verbatim and coded into 143 unique outcomes across 18 outcome domains and four of the five core areas of Dodd's taxonomy^33^: Physiological/Clinical, Life Impact, Resource Use, and Adverse Events (Table 2). The studies reporting each unique outcome are provided in Appendix D.

**TABLE 2:** Outcomes identified in the review, categorized by core area and outcome domain according to Dodd's taxonomy of medical outcomes. Within each outcome domain, outcomes are ordered by the most frequently reported outcome.

Core area	Outcome domain	Outcomes	Verbatim examples of outcome from primary studies	No. studies	Percentage of studies (%)
Adverse events	Adverse events/effects (*N* = 1)	Adverse events	“Adverse events,” “reactions,” “rates of serious adverse events,” “reported adverse effects,” “side effects”	20	14
Life impact	Cognitive functioning (*N* = 38)	Global cognition	“Global cognition,” “neurocognitive function,” “multiple cognitive functions,” “general cognition,” “cognitive performance”	100	69
		Executive function	“Executive function,” “frontal lobe executive function,” “prefrontal cognitive function,” “logical‐executive functions,” “prefrontal cognitive function”	52	36
		Episodic memory	“Episodic memory,” “immediate and delayed memory,” “recent, long‐term visuospatial memory,” “verbal memory,” “recognition memory”	49	34
		Memory	“Memory,” “memory abilities and frequency of memory slips,” “memory function,” “information originally learned that was retained,” “learning and memory”	36	25
		Attention	“Attention,” “attentional control,” “sustained attention,” “focused attention,” “divided attention”	32	22
		Working memory	“Working memory,” “working memory span,” “verbal working memory,” “spatial working memory,” “digit span”	25	17
		Processing speed	“Processing speed,” “information processing speed,” “cognitive processing speed,” "visual processing speed”	17	12
		Visuospatial abilities	“Visuospatial abilities,” “visuo‐perception,” “visuospatial skills,” “visuospatial functions,” “visual search capability”	16	11
		Verbal fluency	“Verbal fluency,” “semantic fluency,” “category fluency,” “phonemic fluency”	15	10
		Subjective memory	“Subjective memory,” “satisfaction with memory ability,” “self‐rated memory ability,” “perceptions of memory impairment,” "memory awareness”	14	10
		Language	“Language,” “verbal function,” “language ability,” “language function”	14	10
		Immediate (short‐term) memory	“Immediate memory,” “short‐term memory,” “immediate recall,” “immediate recognition,” "short‐term memory impairment”	10	7
		Neuropsychology	“Neuropsychological,” “neuropsychological assessment,” “neuropsychological evaluation,” “neuropsychological cognitive,” “neuropsychological testing”	7	5
		Associative memory	“Associative memory,” “associative memory encoding,” “associative learning,” “verbal memory for associated word pairs,” “face‐name associations”	7	5
		Visuo‐constructional abilities	“Visuo‐constructive abilities,” “visuoconstruction,” “visuospatial/constructional“, ”praxis and visuospatial skills", “visual constructive abilities”	7	5
		Logical memory	“Logical memory,” “logical memory function”	6	4
		Subjective cognitive functioning	“Cognitive complaints,” “subjective cognitive problems,” “subjective cognitive functioning,” “subjective cognitive assessment,” “subjective cognitive problems”	5	3
		Memory strategies	“How frequently participants use memory strategies in everyday life,” “memory strategies,” “knowledge and use of memory strategies,” “memory‐strategy knowledge and behaviour”	5	3
		Everyday memory	“Retrospective and prospective memory slips in everyday life,” “everyday memory”	3	2
		Inhibitory control	“Inhibition,” “response inhibition”	3	2
		Semantic memory	“Semantic memory,” “language and semantic memory”	3	2
		Non‐memory cognition	“Non‐memory cognitive functions,” “non‐memory, global cognition”	2	1
		Confidence in cognition and memory	“Subjective confidence in own cognition and memory,” “memory confidence”	2	1
		Everyday cognitive function	“Everyday cognitive function,” “cognitive problems in daily life”	2	1
		Information processing	“Rapid visual information processing,” “complex information processing”	2	1
		Meta‐cognitive ability	“Meta‐cognitive sensitivity,” “meta‐memory”	2	1
		Perceived control over memory	“Perceived control over memory”	2	1
		Abstract thinking	“Abstract thinking”	1	1
		Cognitive flexibility	“Cognitive flexibility”	1	1
		Cognitive/perceptual load	“Dual cognitive task”	1	1
		Concentration	“Concentration”	1	1
		Declarative memory	“Declarative memory”	1	1
		Fluid intelligence	“Fluid intelligence”	1	1
		Prospective memory	“Prospective memory performance”	1	1
		Spiritual wellbeing	“Spiritual well‐being”	1	1
		Understanding of factors affecting memory	“Participants' thoughts about the relationship between lifestyle factors (i.e., stress/relaxation, nutrition, physical activity, and participation in cognitively stimulating activities) and memory”	1	1
		Orientation	“Orientation and spatial attention”	1	1
		Word finding	“Confrontation naming for language”	1	1
	Delivery of care (*N* = 6)	Adherence and tolerability	“Adherence to dance and music programs,” “adherence,” “integration into daily life,” “tolerability and compliance,” “compliance,” “compliance to exercise training programs,” “attendance”	12	
		Satisfaction with the intervention	“Appreciation,” “enjoyment,” “motivation,” “level of satisfaction”	4	3
		Satisfaction with outcomes	“Satisfaction with the outcomes,” "satisfaction”	3	2
		Acceptability and feasibility	“Acceptability and feasibility,” “acceptance of information and communication technologies (ICT),”	2	1
		Long‐term maintenance of intervention	“Post‐intervention practice”	1	1
		Willingness to continue with intervention	“Desire to continue”	1	1
	Emotional functioning/wellbeing (*N* = 14)	Mood	“Mood,” “mood status and depression,” “mood and wellbeing”	15	10
		Psychological well‐being	“Psychological health,” “psychological wellbeing,” “psychological well‐being”	3	2
		Well‐being	“Well‐being,” “general well‐being,” “subjective personal well‐being”	3	2
		Apathy	“Apathy”	3	2
		Self‐esteem	“Individual's beliefs and emotions regarding self‐perception,” “global self‐worth”	2	1
		Stress	“Perceived psychological stress,” “psychological stress”	2	1
		Acceptance	“Acceptance”	2	1
		Helplessness	“helplessness”	2	1
		Mindfulness	“Mindfulness attitudes,” “awareness and attention to present events and experiences”	2	1
		Distress	“Distress”	1	1
		Coping strategies	“Coping strategies”	1	1
		Resilience to stress	“Psychological resilience to stress”	1	1
		Self‐confidence	“Level of self‐confidence”	1	1
		Self‐efficacy	“Self‐efficacy”	1	1
	Global quality of life (*N* = 2)	Quality of life	“Quality of life,” “general quality of life”	16	11
		Health‐related quality of life	“Health‐related quality of life,” “self‐rated health status,” “illness‐induced disruptions to lifestyle and activities that negatively impinge on quality of life”	5	3
	Physical functioning (*N* = 12)	Physical activity levels	“Physical activity,” “exercise,” “physical activity and other lifestyle habits,” “total amount of exercise performed,” “objective measures of physical activity”	13	9
		Balance	“Balance,” “locomotion and dynamic balance ability,” “dynamic balance,” “forward stability,” "balance control”	10	7
		Physical functioning	“Physical function,” “physical functioning”	7	5
		Walking	“Single‐task walking,” “complex walking,” “walking speed”	6	4
		Functional mobility	“Functional mobility,” “mobility”	4	3
		Falls	“Fall incidence,” “overall risk for falls,” “fall risk,” “fear of falling”	4	3
		Gait	“Gait velocity,” “gait performance,” “gait variability,” “gait analysis”	4	3
		Psychomotor skills	“Psychomotor speed,” “motor speed,” “speed,” “motor control”	4	3
		Physical fitness	“Physical fitness,” “senior functional physical fitness,” “changes in physical fitness,” “intensity”	4	3
		Reaction time	“Reaction time,” “ability to react as quickly as possible to basic stimuli,” “psychomotor and personal speed”	3	2
		Confidence in balance	“Perceived confidence in performing balance‐related activities”	1	1
		Number of outdoor activities	“Number of outdoor activities”	1	1
	Role functioning (*N* = 5)	Instrumental activities of daily living	“Instrumental activities of daily living,” “mental capacity to perform daily tasks,” “performance‐based function,” “basic and social functioning,” “individual's self‐sufficiency”	20	14
		Functional ability	"Global functioning,” “functional assessment,” “functional activities,” “daily functionality”	16	11
		Basic activities of daily living	“Activities of daily living,” “daily activities,” “daily self‐care,” “basic activities of elderly individuals,” “everyday activities”	12	8
		Participation in activities	“Participation in activities,” “engagement in leisure activities”	2	1
		Occupational performance	“Occupational performance”	1	1
	Social functioning (*N* = 4)	Relationships with others	“Social and human relationships,” “social support”	2	1
		Conversational ability	“Conversation time”	1	1
		Satisfaction with relationships	“Satisfaction with the partner relationship”	1	1
		Social participation	“Social participation”	1	1
	Personal circumstances (*N* = 1)	Goal‐oriented rehabilitation	“Goal‐oriented rehabilitation”	1	1
Physiological/Clinical	Endocrine outcomes (*N* = 3)	Insulin‐like growth factor 1	“IGF‐1,” “insulin‐like growth factor 1,” “serum IGF‐1”	3	2
		Cortisol	“Cortisol”	1	1
		Cortisol awakening response	“Cortisol awakening”	1	1
	General outcomes (*N* = 10)	Body composition	“Body composition,” “body height and weight,” “anthropometry”	4	3
		Bespoke outcome related to intervention	“Training progress in experimental participants,” “behavioral effects in the MRI,” “fMRI task performance” *We checked this with a PPI group to ensure these outcomes could not be re‐worded in a way that they were understandable as an outcome in their own right*.	2	1
		Traditional Chinese medicine symptoms of dementia	“Syndrome differentiation,” “Shen (Kidney) deficiency”	2	1
		Physical health	“Ratings of physical health,” “health issues”	2	1
		Deqi sensations	“Subjective ‘deqi’ sensations”	1	1
		Fibroblast growth factor‐2	“Serum FGF‐2”	1	1
		Sensory functioning	“Olfactory discrimination, taste identification, olfactory recognition”	1	1
		Sleep quality	“Sleep quality”	1	1
		Alcohol intake	“Alcohol use”	1	1
		Telomere length	“Telomere length”	1	1
	Immune system outcomes (*N* = 5)	Cytokines	“cytokines,” “plasma IL‐10 levels,” “TNF‐alpha,” “plasma TNF‐α”	4	3
		C‐reactive protein	“CRP,” “high‐sensitivity C‐reactive protein”	2	1
		Platelet factor 4	“Platelet factor 4”	1	1
		Peripheral blood monocytes	“Peripheral blood monocytes”	1	1
		Phagocytic activity	“Phagocytic activity”	1	1
	Metabolism and nutrition outcomes (*N* = 10)	Cholesterol	“Total cholesterol,” “cholesterol levels,” “low‐density lipoprotein cholesterol”	4	3
		Insulin	“Insulin”	3	2
		Insulin sensitivity	“Insulin sensitivity,” “insulin resistance,” “glucose metabolism”	2	1
		Hemoglobin A1c	“HbA1c”	2	1
		Erythrocytes fatty acid membrane composition	“Plasma erythrocyte membrane fatty acid compositions,” “erythrocyte membrane fatty acid”	2	1
		Leptin	“Leptin”	2	1
		Adiposity	“Adiposity”	1	1
		Vitamin B metabolism	“B vitamin metabolism”	1	1
		Homocysteine	“Total homocysteine”	1	1
		Omega‐3 intake	“Omega‐3 fatty acid intake”	1	1
	Musculoskeletal and connective tissue outcomes (*N* = 3)	Muscular strength	“Variation in muscle strength,” “strength,” “lower extremity muscle strength”	4	3
		Handgrip strength	“Maximum hand grip strength,” “grip strength,” “hand grip strength,” “handgrip strength (HGS) of the non‐dominant hand”	4	3
		Muscle endurance (lower limbs)	“Muscle endurance of the lower limbs”	1	1
	Nervous system outcomes (*N* = 16)	Brain volume or structure	“Brain volume,” “total intracranial volume,” “rate of volumetric changes,” “ventricular volume,” “white matter lesions”	16	11
		Functional connectivity	“Functional connectivity,” “functional connectivity density,” “resting brain networks,” “resting state functional connectivity,” “hippocampal functional connectivity,” “DMN functional connectivity,” “functional connectivity within DMN,” “connectivity within memory‐related areas”	11	8
		Resting state or spontaneous brain activity	“Resting state change of brain activity,” “spontaneous brain activities,” “brain spontaneous activity change,” “brain activity,” “improvement in long‐term brain activity”	10	7
		Brain activation during a cognitive task	“Brain activation,” “memory fMRI activation,” “fMRI activation changes,” “activation changes during encoding,” “blood oxygen level dependent (BOLD) signal elicited by a cognitive task”	10	7
		Brain‐derived neurotrophic factor	“Plasma BDNF,” “serum BDNF,” “BDNF,” “changes in BDNF levels”	5	3
		Progression to dementia (incidence)	“Conversion to AD,” “reduction of the risk of transition to dementia,” “screen for dementia,” “dementia,” “progression to dementia”	5	3
		Dementia severity	“Clinical dementia rating,” “dementia severity,” “overall dementia severity,” “stage level”	4	3
		Severity of symptoms	“Global deterioration,” “disease severity and progression of illness,” “improvement”	4	3
		Brain metabolism	“Brain glucose metabolism,” “neuroimaging…F‐FDG‐PET acquisitions,” “brain metabolism…FDG PET”	3	2
		Amyloid beta burden	“Aß40,” CSF Aß40,” “Aß42,” “CSF Aß42,” “brain Aβ deposition”	3	2
		Brain function	“Brain function,” “brain function change”	2	1
		Brain perfusion and cerebrovascular health	“Brain perfusion,” “cerebrovascular hemodynamics,” “modifications in cerebral blood flow”	2	1
		EEG frequency	“EEG frequency,” “delta/alpha ratio,” “theta/beta ratio,” “theta/alpha ratio”	2	1
		Time to progression to dementia	“Time to the clinical diagnosis of AD,” “time to the development of possible or probable Alzheimer's disease dementia”	2	1
		EEG signatures	“Neurophysiological performance”	1	1
		Tau burden	“CSF tau”	1	1
	Psychiatric outcomes (*N* = 6)	Depression	“Depression,” “depressive symptoms,” “symptoms of depression,” “depressive mood,” “subjective experience of depression”	35	24
		Anxiety	“Anxiety,” “anxiety level,” “anxiety symptoms”	7	5
		Neuropsychiatric symptoms	“Psycho‐affective symptoms,” “neuropsychiatric symptoms”	5	3
		Behavioral disturbance	“Behavioral assessment,” “behavioral disturbances”	2	1
		Rumination	“Changes in ruminations”	1	1
		Behavioral and psychological symptoms of dementia (BPSD)	“Behavioral and psychological symptoms (BPSD)”	1	1
	Respiratory, thoracic, and mediastinal outcomes (*N* = 2)	Cardiorespiratory fitness	“Cardiorespiratory fitness”	7	5
		Aerobic fitness	“Aerobic fitness,” “VO2 max,” “aerobic conditioning”	3	2
	Vascular outcomes (*N* = 3)	Cardiovascular health	“Cardiovascular outcomes,” “cardiovascular fitness,” “intima media thickness of the distal right and left common carotid artery”	3	2
		Vascular endothelial growth factor	“Serum VEGF,” “VEGFR1”	2	1
		Blood pressure	“Arterial blood pressure”	1	1
Resource Use	Societal/carer burden (*N* = 1)	Caregiver burden	“Burden of caregiving,” “caregiver burden”	5	3

There was substantial heterogeneity in how verbatim outcomes were worded and the level of detail or granularity provided (Table 2). Eighty‐nine (61%) outcomes were reported by three or fewer studies, with around one‐third of outcomes (46/144, 32%) reported by only a single study. Global cognition was the most commonly reported outcome, and the only outcome reported by over half of included studies. Only six outcomes were reported by at least 20% of included studies: global cognition, executive function, episodic memory, memory, depression, and attention.

#### 3.3.1 Life Impact

Eighty‐four Life Impact outcomes were reported covering eight outcome domains: cognitive functioning, emotional functioning/wellbeing, physical functioning, social functioning, role functioning, global quality of life, personal circumstances, and delivery of care.

##### 3.3.1.1 Cognitive functioning

**FIGURE 4:**
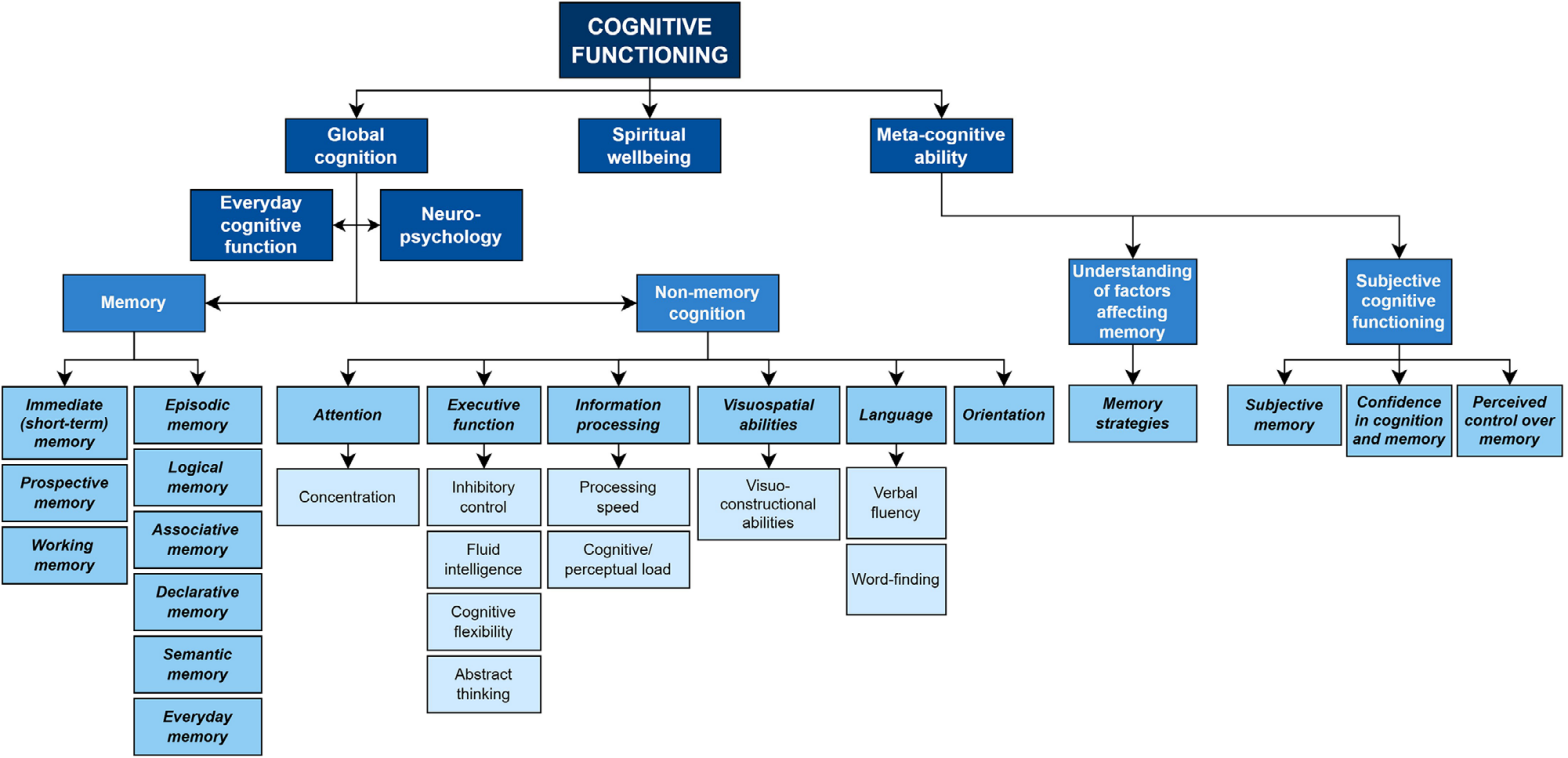
Schematic showing hierarchy of unique outcomes for cognitive functioning domain.

In [Fig alz71800-fig-0003]total, 38 unique outcomes relating to cognitive functioning were reported at different levels of granularity (Figure 4). Broadly, the outcomes mostly related to overall cognitive functioning and specific domains and subdomains of cognitive functioning such as memory, attention, executive functioning, language, and orientation. Additional outcomes related to meta‐cognitive abilities and spiritual well‐being.

The top five most frequently reported outcomes across all studies related to the cognitive functioning outcome domain. Global cognition was the most frequently reported outcome, followed by executive function, episodic memory, attention, and memory. Global cognition was typically measured using one or more standardized cognitive assessments covering multiple domains such as the Mini‐Mental State Examination (MMSE)^44^ or Montreal Cognitive Assessment (MoCA)^45^ or a combination of domain‐specific assessments (e.g., digit span, word list recall, drawing, digit‐symbol substitution).

Memory was frequently reported across different studies in different ways. Ten outcomes were specific subtypes of memory (e.g., prospective memory, semantic memory, associative memory), while three outcomes related to meta‐memory (understanding of factors affecting memory, perceived control over memory, and memory strategies). However, modalities were collapsed (e.g., verbal memory, visuospatial memory) to prevent an excessive number of unique memory outcomes and because these were often not explicitly stated within papers.

##### 3.3.1.2 Physical functioning

Fourteen physical functioning outcomes were identified. Broadly, these outcomes were related to balance, mobility, physical fitness, and activity levels. Physical activity was the most frequently reported outcome and was often measured using either self‐report or actigraphy.

##### 3.3.1.3 Emotional functioning/well‐being

Fourteen unique outcomes were identified relating to emotional functioning/well‐being and covered general mood and well‐being outcomes, adjusting and accepting the MCI diagnosis and related symptoms, and attitudes toward the patient's self (e.g., self‐esteem, self‐confidence). Mood was typically measured with depression and anxiety scales, with other aspects of emotional functioning and well‐being measured with more specific scales (e.g., self‐efficacy for managing MCI scale,^46^ Brief‐COPE^47^).

##### 3.3.1.4 Delivery of care

Six delivery‐of‐care outcomes were identified which related to participants’ subjective experience of interventions and outcomes relating to adherence and feasibility.

##### 3.3.1.5 Role functioning

Five role functioning outcomes were identified. The most common role functioning outcome was instrumental activities of daily living (IADLs), which was typically assessed with a specific IADL scale or performance‐based assessment. The second most common physical functioning outcome was the broader outcome of functional ability, which was measured by a wide range of assessments (e.g., cognitive scales, activities of daily living [ADL] or IADL scales, Clinical Global Impression scales).

##### 3.3.1.6 Social functioning

Four outcomes relating to social functioning were identified, with three outcomes reported by a single study. Aspects of social functioning covered included social participation/engagement, conversational ability, and relationships with partners, family, and friends.

##### 3.3.1.7 Global quality of life

Two global quality of life outcomes were identified, quality of life and health‐related quality of life, which were reported across 15 and five studies, respectively. The RAND 36‐item Health Survey,^47^ the EuroQol 5‐Dimension 5‐Level questionnaire (EQ‐5D‐5L),^48^ and Quality of Life‐Alzheimer's Disease^49^ scales were frequently used to measure quality of life.

##### 3.3.1.8 Personal circumstances

One outcome relating to personal circumstances, goal‐oriented rehabilitation, was identified. It was measured in a single case study^38^ using bespoke goal attainment ratings and the Canadian Occupational Performance Measure.^50^

#### 3.3.2 Physiological/Clinical

Fifty‐eight Physiological/Clinical outcomes were reported covering nine outcome domains: nervous system outcomes; psychiatric outcomes; immune system outcomes; metabolism and nutrition outcomes; endocrine outcomes; respiratory, thoracic, and mediastinal outcomes; musculoskeletal and connective tissue outcomes; vascular outcomes; and general outcomes.

##### 3.3.2.1 Nervous system outcomes

Sixteen nervous system outcomes were reported, including outcomes relating to neuropathology (e.g., amyloid burden, tau burden), brain structure and function, and progression to dementia. The most frequently reported outcome was brain volume or structure followed by functional connectivity.

##### 3.3.2.2 Metabolism and nutrition outcomes

Ten metabolism and nutrition outcomes were identified and included outcomes usually measured in the blood and related to glucose metabolism, lipid and fatty acid metabolism, and vitamin and amino acid metabolism.

##### 3.3.2.3 General outcomes

Nine general outcomes were reported, covering physical health, sleep, traditional Chinese medicine outcomes, and a signaling protein relating to cell growth, tissue repair, and angiogenesis (fibroblast growth factor 2). An additional general outcome, bespoke outcome related to intervention, was generated to capture three instances in two studies where reviewers and lived experience experts agreed that the outcome was highly intervention‐specific and it was unclear how to extrapolate to another outcome. Body composition was the most commonly reported general outcome and was typically calculated using height and weight.

##### 3.3.2.4 Psychiatric outcomes

Six psychiatric outcomes were reported and included both generic terminology (e.g., behavioral and psychological symptoms of dementia) and more specific psychiatric conditions and symptoms (e.g., anxiety, rumination). The most frequently reported psychiatric outcome was depression, which was often measured using standardized questionnaires such as the Geriatric Depression Scale.^51^

##### 3.3.2.5 Immune system outcomes

Five immune system outcomes were reported covering immune cells, immune signaling, and phagocytosis. All were measured in the blood.

##### 3.3.2.6 Endocrine outcomes

Three endocrine outcomes (cortisol, cortisol awakening response, and insulin‐like growth factor 1 [IGF‐1]) were reported. Cortisol awakening response was measured using serial saliva sampling, with cortisol and IGF‐1 measured in the blood.

##### 3.3.2.7 Vascular outcomes

Three vascular outcomes were identified. Cardiovascular health was the most commonly reported vascular outcome and was measured in various ways including lipids, blood pressure, maximal oxygen consumption (VO2 max), and thickness of the carotid artery.

##### 3.3.2.8 Musculoskeletal and connective tissue outcomes

Three outcomes relating to muscular strength and muscle endurance were categorized as musculoskeletal and connective tissue outcomes and were measured using a sit‐stand test, how much weight someone could lift, and handgrip strength.

##### 3.3.2.9 Respiratory, thoracic, and mediastinal outcomes

Two related outcomes, cardiorespiratory fitness and aerobic fitness, were reported and categorized as respiratory, thoracic, and mediastinal outcomes but could also have been categorized as cardiac outcomes as they related to both cardiovascular and respiratory health. VO2 max was typically used as a measure of cardiorespiratory or aerobic fitness.

#### 3.3.3 Resource use

A single outcome relating to societal/carer burden, caregiver burden, was reported across five studies.

#### 3.3.4 Adverse events/effects

A single outcome, adverse events, was reported across 20 studies.

#### 3.3.5 Outcome measures

Several challenges emerged in mapping outcomes and outcome measurement tools. Multiple outcome measures were used for a single outcome, outcome measures were listed without defining the specific outcome of interest, and outcomes and outcome measures could be listed separately without clarification of which tools were measuring which outcome. Additionally, a single outcome measure could be used to operationalize different outcomes across different studies. For example, verbal fluency tests were used to measure verbal fluency as an outcome itself, as well as executive function or as part of a cognitive battery for global cognition, and the Stroop Color and Word test^52^ was used to measure attention, executive function, and psychomotor skills. The most frequently used outcome measures are described in Appendix E.

## 4 DISCUSSION

This review provided a systematic overview of outcomes, outcome domains, and outcome measures used in interventional studies for community‐dwelling adults with MCI. We identified substantial heterogeneity in both what outcomes were reported and how outcomes were measured. The results confirm the need for a COS for MCI to support future evidence synthesis and ensure that the most meaningful outcomes to stakeholders are included across interventional studies.

A decline in cognition without a significant impact on ability to function independently is the defining clinical characteristic of MCI.^53^ In line with this, we found that outcomes relating to cognitive functioning were prioritized and often included at different levels of granularity, ranging from global cognition to word finding. In contrast, functional, social, or quality of life outcomes were reported by relatively few studies.^54^, ^55^ Cognitive difficulties may have a notable, albeit mild, impact on everyday functioning, such as experiencing more challenges dealing with financial matters, difficulty making or keeping plans, or decreased involvement in hobbies.^53^, ^56^ In addition to cognition, outcomes that more closely translate to an individual's experience of day‐to‐day life are important to individuals with MCI and those who care for them.^57^ The outcomes included within this review likely reflect those considered important by researchers, clinicians, and funders and may not necessarily represent outcomes of greatest importance to individuals with MCI or those who support them day‐to‐day, such as partners or relatives.^58^ To ensure that stakeholder views are represented within the final COS, the outcomes from this review will be collated with outcomes identified through one‐to‐one interviews with individuals with MCI, their partners, and relatives, as well as professionals who support their health and well‐being.^21^ The findings from the interviews and final COS will be reported elsewhere.

We followed recent recommendations^34^ to define a “unique” outcome as “one that has original meaning and context” and collated assessments across different time points to reduce duplication of similar outcomes. However, deciding on whether an outcome was unique remained a challenge. Studies described outcomes in varying terminology and granularity, particularly for cognitive functioning, physical functioning, and across physiological/clinical domains. Granularity has recently received more attention in COS development, as it has important implications for perceptions of outcome reporting heterogeneity, as well as the specificity of outcomes included in the final COS.^59^ Ambiguity in whether outcomes represented the same or different outcomes and how granular outcomes should be was resolved via iterative review against Dodd's taxonomy^33^ and discussions between lived experience experts from a PPI group and the study management group who provided multidisciplinary input and expertise. For example, our lived experience experts felt strongly that “memory” was not sufficiently granular despite being two levels beneath the outcome domain “cognitive functioning,” as different types of memory were more likely to be affected in MCI than others and may be assessed very differently. Granularity of outcomes will be reviewed again following evidence synthesis with outcomes identified in interviews ahead of the Delphi study and at the consensus meeting.^21^ Nonetheless, it is possible that other researchers may have categorized the outcomes with greater or less granularity, and we support the guidance recently published that now recommends more explicitly specifying the level of granularity in the protocol for future COS development.^59^

Most outcomes were measured using validated questionnaires or assessments (e.g., MMSE,^44^ Geriatric Depression Scale^51^) and physiological measurements taken via blood samples or imaging, though some studies also used unvalidated bespoke measures, subtests, or composite scores as outcome measures. We also identified several outcome measures that had been specifically adapted for use in individuals with MCI, including the Activities of Daily Living for Mild Cognitive Impairment scale,^60^ the Alzheimer's Disease Assessment Scale–Cognitive Subscale‐MCI,^61^ and the Self‐Efficacy for Managing MCI scale.^62^ However, outcome measurements specifically tailored for MCI were rarely utilized.

Identifying clinically meaningful change in MCI can be challenging due to the subtle symptomatic changes observed in MCI,^20^ high variability in premorbid ability,^63^ and compensatory mechanisms that may mask difficulties.^64^, ^65^ We found that some outcome measurement instruments were used to measure many different outcomes, which could be related both to different levels of outcome granularity (e.g., story recall to measure memory, logical memory, declarative memory) and to different interpretations of what the tool was designed to measure. For example, our review identified studies using the Stroop Color and Word test^52^ to measure psychomotor skills, reaction time, executive function, attention, and inhibitory control. Disagreement on the interpretation of outcome measurement instruments, particularly on what cognitive outcomes are being measured, has been reported^52^ and likely reflects how even seemingly straightforward tasks involve multiple cognitive or physiological processes.^66^ Therefore, in addition to the COS, a core measurement set recommending which outcome measurement instruments should be used for each core outcome is also warranted to ensure the most appropriate available instruments are selected for use in clinical trials.

Though it was not the focus of this review, we noted that the quality of outcome reporting was heterogeneous. Some studies clearly defined outcome variables and explicitly mapped outcomes to outcome measurement instruments, while this information was more implicit or vague in other papers. Similarly, whether variables were considered trial outcomes, baseline characteristics, or screening measures was not always clear. Reporting guidelines published in recent years, such as the CONSORT‐Outcomes 2022 extension, highlight the increasing focus on outcome reporting and may help to increase transparency and support interpretation of future trials.^67^

### 4.1 Limitations

This review included a large number of studies published internationally across 20 years of MCI research, examining effectiveness across a range of intervention types. The choice of a scoping umbrella review was suitable for our review purposes to identify outcomes for consideration in a COS for MCI, given the breadth of intervention types and high number of trials.^21^, ^29^ However, we recognize several limitations.

First, the high number of eligible reviews and retained articles following our comprehensive literature search resulted in our reviewing and adapting our methodology. The decision to stratify reviews by intervention type resulted in many retained articles and reviews that were representative in terms of intervention type while keeping the scale of the review feasible. It is possible that additional unique outcomes could have been identified by reviewing the 28 additional reviews that were eligible. However, nearly all unique outcomes were reported by at least two studies, suggesting that we reached outcome saturation.

Second, as only studies published in reviews at the time of the literature search were included, the review did not encompass the most recently published studies, including those of second‐generation monoclonal antibodies such as lecanemab and donanemab. However, the outcomes included in such trials focused on cognitive, functional, and safety outcomes, which were identified by our review from earlier trials. For example, lecanemab was evaluated against its ability to slow cognitive and functional decline and reduce amyloid burden in the CLARITY‐AD trial,^68^ and TRAILBLAZER ALZ‐2^69^ utilized integrated composite outcomes that combined established cognitive and functional outcome measures to test the efficacy of donanemab. We note there may be changing trends in the most common outcomes and how they are measured, for example, increasing utilization of biomarkers both to determine eligibility and as trial outcomes.^70^ However, we do not know of any “missed” outcomes used in recent trials but not picked up by our review.

The review was limited to studies reported in English, resulting in the exclusion of non‐English articles published within reviews, predominantly studies of traditional Chinese medicine. Our original protocol specified that more than 70% of studies contained within a review were required to be eligible at full‐text review, which would have excluded a high proportion of traditional Chinese medicine studies published in English, exacerbating the bias. Therefore, we adjusted the protocol to remove the articles only published in another language from the denominator used to calculate this eligibility, which retained more traditional Chinese medicine studies. Restricting reviews to English‐language publications is considered an accepted approach for reviews with limited resources, and the impact on the results in meta‐analyses has been considered “negligible,”^71^ though it remains possible that outcomes used in non‐English‐speaking countries are different.

Finally, in addition to relevance, outcome choice and measurement are likely influenced by sensitivity to change, feasibility, and acceptability (e.g., cognitive testing is more readily available, less expensive, and lower burden than amyloid positron emission tomography).^61^, ^72^, ^73^ Requirements from funders or regulatory agencies may also impact decisions. For example, the United States Food and Drug Administration specifically recommend measuring cognition and daily functioning in drug trials for AD‐MCI.^74^ While our review captures the breadth and frequency of outcomes and measures used, it does not capture the rationale behind the choice of outcome. Through qualitative interviews, we will explore what outcomes are important to different stakeholder groups in MCI and provide additional context regarding why these outcomes are meaningful.

### 4.2 Conclusions

The heterogeneity in outcomes reported in MCI interventional trials provides a rationale for the development of a COS to promote standardization in future trials of the most meaningful outcomes. Identifying a COS will help to standardize outcome reporting, allowing for better synthesis and comparisons in future literature reviews and meta‐analyses, and encourage transparent outcome reporting of the most meaningful outcomes for individuals living with MCI.

## AUTHOR CONTRIBUTIONS

**Victoria Grace Gabb**: Conceptualization; project administration; methodology; investigation; data curation; formal analysis; visualization; writing—original draft; writing—review and editing. **Sam Harding**: Methodology; investigation; writing—review and editing; supervision. **Tomas Lemke**: Investigation; writing—review and editing. **Sophie Alderman**: Investigation; writing—review and editing. **Natalie Woodward**: Investigation; writing—review and editing. **Julie Clayton**: Writing—review and editing; funding acquisition. **Angus McNair**: Writing—review and editing; funding acquisition. **Winsome Barrett‐Muir**: Writing—review and editing; funding acquisition. **Alan Richardson**: Writing—review and editing; funding acquisition. **Jemima Dooley**: Writing—review and editing; funding acquisition. **Sarah Rudd**: Methodology; Investigation; resources; writing—review and editing. **Elizabeth Coulthard**: Conceptualization; writing—review and editing; supervision; funding acquisition. **Nicholas Turner**: Conceptualization; writing—review and editing; supervision; funding acquisition.

## CONFLICT OF INTEREST STATEMENT

The authors declare no conflicts of interest. Author disclosures are available in the Supporting Information.

## Supporting information



Supporting Information

Supporting Information

Supporting Information

Supporting Information

Supporting Information

Supporting Information
