# Supplementary material for: Outcomes reported in interventional studies for mild cognitive impairment: a scoping umbrella review to inform core outcome set development
Source: Alzheimers Dement. 2026 Sep 5;22(9):e71800. doi: 10.1002/alz.71800 (PMC13546403; doi:10.1002/alz.71800)
Supplement: Supplementary file 2 [file ALZ-22-e71800-s001.docx]

Supplementary Materials – Appendix A

Search strategies

# Embase/Ovid MEDLINE

Embase <1974 to 2023 August 04>

Ovid MEDLINE(R) ALL <1946 to August 04, 2023>

1	((Mild or minimal* or Early) adj3 ((Cognit* or Neurocognit* or memory) adj1 (disorder* or impair* or declin* or deficit* or dysfunction*))).ti,ab.	73023

2	"Cognitive Impairment No Dementia".ti,ab.	761

3	COGNITIVE DYSFUNCTION/	176150

4	NEUROCOGNITIVE DISORDERS/	12500

5	MEMORY DISORDERS/	47668

6	(MCI or MNCD or MCD or NMCI or MMCI or SMCI or MCIA).ti,ab.	77745

7	or/1-6	316688

8	(systematic review or meta-analysis).pt.	318010

9	meta-analysis/ or systematic review/ or systematic reviews as topic/ or meta-analysis as topic/ or "meta analysis (topic)"/ or "systematic review (topic)"/ or network meta-analysis/	953853

10	((systematic* or scoping or methodologic*) adj3 (review* or overview*)).ti,ab,kf.	755697

11	((quantitative adj3 (review* or overview* or synthes*)) or (research adj3 (integrati* or overview*))).ti,ab,kf.	34224

12	((integrative adj3 (review* or overview*)) or (collaborative adj3 (review* or overview*)) or (pool* adj3 analy*)).ti,ab,kf.	94576

13	(data synthes* or data extraction* or data abstraction*).ti,ab,kf.	90986

14	(handsearch* or hand search*).ti,ab,kf.	24836

15	(mantel haenszel or peto or der simonian or dersimonian or fixed effect* or latin square*).ti,ab,kf.	83538

16	(met*analy* or met?analy*).ti,ab,kf.	19757

17	(meta regression* or metaregression*).ti,ab,kf.	32816

18	(meta-analy* or metaanaly* or systematic review*).mp,hw.	1199659

19	(medline or cochrane or pubmed or medlars or embase or cinahl).ti,ab,hw.	797171

20	(cochrane or (health adj2 technology assessment) or evidence report).jw.	52724

21	(comparative adj3 (efficacy or effectiveness)).ti,ab,kf.	43597

22	(outcomes research or relative effectiveness).ti,ab,kf.	27505

23	((indirect or indirect treatment or mixed-treatment or bayesian) adj3 comparison*).ti,ab,kf.	11905

24	(multi* adj3 treatment adj3 comparison*).ti,ab,kf.	717

25	(mixed adj3 treatment adj3 (meta-analy* or metaanaly*)).ti,ab,kf.	439

26	umbrella review*.ti,ab,kf.	3169

27	(multi* adj2 paramet* adj2 evidence adj2 synthesis).ti,ab,kf.	44

28	(multiparamet* adj2 evidence adj2 synthesis).ti,ab,kf.	37

29	(multi-paramet* adj2 evidence adj2 synthesis).ti,ab,kf.	37

30	or/8-29	1666527

31	and/7,30	15514

32	exp animals/ not humans/	16795265

33	31 not 32	14182

34	33	14182

35	limit 34 to english language	13787

36	35 use medall	3994

37	cognition disorder/ or mild cognitive impairment/	343953

38	1 or 2 or 4 or 5 or 6 or 37	444021

39	30 and 38	21853

40	(exp ANIMAL/ or NONHUMAN/) not exp HUMAN/	12268638

41	39 not 40	21600

42	limit 41 to english language	20911

43	limit 42 to "remove medline records"	11409

44	43 use oemezd	5374

# CINAHL (EbscoHost)

| **#** | **Query** |
| --- | --- |
| S1 | TI((Mild or "age associate*" or early or minimal* or Early or prodromal or "age related" or "associated with age") N3 ((Cognit* or Neurocognit* or memory) N1 (disorder* or impair* or declin* or deficit* or dysfunction*))) OR AB((Mild or "age associate*" or early or minimal* or Early or prodromal or "age related" or "associated with age") N3 ((Cognit* or Neurocognit* or memory) N1 (disorder* or impair* or declin* or deficit* or dysfunction*))) |
| S2 | TI("Cognitive Impairment No Dementia") or AB("Cognitive Impairment No Dementia") |
| S3 | (MH "Cognition Disorders") |
| S4 | (MH "Mild Cognitive Impairment") |
| S5 | (MH "Memory Disorders") |
| S6 | TI(MCI or MNCD or AAMI or AACD or AACD or MCD or ARCD or NMCI or AMCI or MMCI or SMCI or MCIA) or AB(MCI or MNCD or AAMI or AACD or AACD or MCD or ARCD or NMCI or AMCI or MMCI or SMCI or MCIA) |
| S7 | S1 OR S2 OR S3 OR S4 OR S5 OR S6 |
| S8 | (MH "meta analysis" OR MH "systematic review" OR MH "Scoping Review" OR PT "systematic review" OR PT "meta analysis" OR PT review OR (((TI systematic* OR AB systematic*) or (TI methodologic* OR AB methodologic*) or (TI quantitative OR AB quantitative) or (TI scoping or AB scoping)) N3 ((TI review* OR AB review*) OR (TI overview* OR AB overview*) or OR (TI synthes* OR AB synthes*))) OR ((TI research OR AB research) N3 ((TI integrati* OR AB integrati*) OR (TI overview* OR AB overview*))) OR (((TI integrative OR AB integrative) N3 ((TI review* OR AB review*) OR (TI overview* OR AB overview*))) OR ((TI collaborative OR AB collaborative) N3 ((TI review* OR AB review*) OR (TI overview* OR AB overview*))) OR ((TI pool* OR AB pool*) N3 (TI analy* OR AB analy*))) OR ((TI "data synthes*" OR AB "data synthes*") OR (TI "data extraction*" OR AB "data extraction*") OR (TI "data abstraction*" OR AB "data abstraction*")) OR ((TI handsearch* OR AB handsearch*) OR (TI "hand search*" OR AB "hand search*")) OR ((TI "mantel haenszel" OR AB "mantel haenszel") OR (TI peto OR AB peto) OR (TI "der simonian" OR AB "der simonian") OR (TI dersimonian OR AB dersimonian) OR (TI "fixed effect*" OR AB "fixed effect*") OR (TI "latin square*" OR AB "latin square*")) OR ((TI "meta analy*" OR AB "meta analy*") OR (TI metanaly* OR AB metanaly*)) OR ((TI "meta regression*" OR AB "meta regression*") OR (TI metaregression* OR AB metaregression*)) OR (MW meta-analy* OR MW "systematic review*" OR MW scoping review ) OR ((TI medline OR AB medline OR MW medline) OR (TI cochrane OR AB cochrane OR MW cochrane) OR (TI pubmed OR AB pubmed OR MW pubmed) OR (TI medlars OR AB medlars OR MW medlars) OR (TI embase OR AB embase OR MW embase) OR (TI cinahl OR AB cinahl OR MW cinahl)) OR (SO Cochrane) OR ((TI comparative OR AB comparative) N3 ((TI efficacy OR AB efficacy) OR (TI effectiveness OR AB effectiveness))) OR ((TI "outcomes research" OR AB "outcomes research") OR (TI "relative effectiveness" OR AB "relative effectiveness")) OR (((TI indirect OR AB indirect) OR (TI "indirect treatment" OR AB "indirect treatment") OR (TI mixed-treatment OR AB mixed-treatment) OR (TI bayesian OR AB bayesian)) N3 (TI comparison* OR AB comparison*)) OR ((TI multi* OR AB multi*) N3 (TI treatment OR AB treatment) N3 (TI comparison* OR AB comparison*)) OR ((TI mixed OR AB mixed) N3 (TI treatment OR AB treatment) N3 ((TI meta-analy* OR AB meta-analy*) OR (TI metaanaly* OR AB metaanaly*))) OR (TI "umbrella review*" OR AB "umbrella review*") OR (TI “scoping review” or AB “scoping review”) OR ((TI multi* OR AB multi*) N2 (TI paramet* OR AB paramet*) N2 (TI evidence OR AB evidence) N2 (TI synthesis OR AB synthesis)) OR ((TI multiparamet* OR AB multiparamet*) N2 (TI evidence OR AB evidence) N2 (TI synthesis OR AB synthesis)) OR ((TI multi-paramet* OR AB multi-paramet*) N2 (TI evidence OR AB evidence) N2 (TI synthesis OR AB synthesis)) |
| S9 | S7 AND S8 |
| S10 | (MH "Vertebrates+") NOT (MH "Human") |
| S11 | S9 NOT S10 |

# PsycInfo (Proquest)

| Set# | Searched for | Results |
| --- | --- | --- |
| S1 | TI,ab((Mild or minimal* or Early) N/3 ((Cognit* or Neurocognit* or memory) N/1 (disorder* or impair* or declin* or deficit* or dysfunction*))) | 16739 |
| S2 | MAINSUBJECT.EXACT("Cognitive Impairment") | 44368 |
| S3 | MAINSUBJECT.EXACT("Mild Cognitive Impairment") OR MAINSUBJECT.EXACT("Memory Disorders") OR MAINSUBJECT.EXACT("Neurocognitive Disorders") | 29485 |
| S4 | Ti,ab(MCI or MNCD or MCD or NMCI or MMCI or SMCI or MCIA) | 9351 |
| S5 | [S1] OR [S2] OR [S3] OR [S4] | 68214 |
| S6 | ME(“systematic review” or “meta-analysis”) | 64842 |
| S7 | MAINSUBJECT.EXACT("Meta Analysis") OR MAINSUBJECT.EXACT("Systematic Review") | 6054 |
| S8 | ti,ab,if((systematic* or scoping or methodologic*) near/2 (review* or overview*)) | 64291 |
| S9 | Ti,ab,if((quantitative near/2 (review* or overview* or synthes*)) or (research near/2 (integrati* or overview*))) | 9129 |
| S10 | Ti,ab,if((integrative near/2 (review* or overview*)) or (collaborative near/2 (review* or overview*)) or (pool* near/2 analy*)) | 6308 |
| S11 | Ti,ab,if(“data synthes*” or “data extraction*” or “data abstraction*”) | 3858 |
| S12 | Ti,ab,if(handsearch* or “hand search*”) | 1575 |
| S13 | Ti,ab,if(“mantel Haenszel” or peto or “der simonian” or dersimonian or ("fixed effect") or ("latin squares")) | 1750 |
| S14 | Ti,ab,if(“met*-analy*” or metanaly* or metaanaly*) | 55242 |
| S15 | Ti,ab,if(("meta regression") or metaregression*) | 2456 |
| S16 | Ti,ab(medline or cochrane or pubmed or medlars or embase or cinahl) | 35992 |
| S17 | pub(cochrane or (health near/1 “technology assessment”) or “evidence report”) | 13 |
| S18 | ti,ab,if(comparative near/2 (efficacy or effectiveness)) | 2453 |
| S19 | Ti,ab,if(“outcomes research” or “relative effectiveness”) | 3864 |
| S20 | Ti,ab,if((indirect or “indirect treatment” or mixed-treatment or bayesian) near/2 comparison*) | 528 |
| S21 | Ti,ab,if(multi* near/2 treatment near/2 comparison*) | 67 |
| S22 | Ti,ab,if(mixed near/2 treatment near/2 (meta-analy* or metaanaly*)) | 18 |
| S23 | Ti,ab,if(“umbrella review*”) | 270 |
| S24 | Ti,ab,if(multi* near/1 paramet* near/1 evidence near/1 synthesis) | 2 |
| S25 | Ti,ab,if(multiparamet* near/1 evidence near/1 synthesis) | 8 |
| S26 | Ti,ab,if(“multi-paramet*” near/1 evidence near/1 synthesis) | 2 |
| S27 | [S6] OR [S7] OR [S8] OR [S9] OR [S10] OR [S11] OR [S12] OR [S13] | 98641 |
| S28 | [S14] OR [S15] OR [S16] OR [S17] OR [S18] OR [S19] OR [S20] OR [S21] OR [S22] OR [S23] OR [S24] OR [S25] OR [S26] | 86328 |
| S29 | [s27] AND [S5] | 2250 |
| S30 | [s28] AND [S5] | 2114 |

# PROSPERO

1. (early or mild or minimal) NEAR3 neurocognit* = 111

2. (early or mild or minimal) NEAR3 memory = 0

3. (early or mild or minimal) NEAR3 cognitive = 1788

# COMET

Mild cognitive (n=7) 1 removed

Cognitive dysfunction (n=14) 14 removed

Memory (n=6) 5 removed

Neurocognitive (n=15) 15 removed
