# Supplementary material for: Outcomes reported in interventional studies for mild cognitive impairment: a scoping umbrella review to inform core outcome set development
Source: Alzheimers Dement. 2026 Sep 5;22(9):e71800. doi: 10.1002/alz.71800 (PMC13546403; doi:10.1002/alz.71800)
Supplement: Supplementary file 3 [file ALZ-22-e71800-s002.docx]

Supplementary Materials –

Appendix D

Outcomes extracted from included primary articles

For each outcome identified within a primary article, wording was extracted verbatim before being coded as a cleaned outcome and categorised into Dodd’s outcome taxonomy (core area and outcome domain).

| Study ID | First Author | Verbatim Outcome | Cleaned Outcome | Dodd's taxonomy:  Core Area | Dodd's taxonomy:  Outcome Domain |
| --- | --- | --- | --- | --- | --- |
| 32 | Rojas | Abstract thinking | abstract thinking | life impact | cognitive functioning |
| 81 | Djabelkhir | acceptance of information and communication technologies (ICT) | acceptability and feasibility | life impact | delivery of care |
| 147 | Marciniak | acceptability and feasibility | acceptability and feasibility | life impact | delivery of care |
| 3 | Joosten-Weyn Banningh | Acceptance | acceptance | life impact | emotional functioning/wellbeing |
| 36 | Joosten-Weyn Banningh | Acceptance / general wellbeing and quality of life | acceptance | life impact | emotional functioning/wellbeing |
| 21 | Jean | tolerability and compliance | adherence and tolerability | life impact | delivery of care |
| 30 | Greenaway | adherence | adherence and tolerability | life impact | delivery of care |
| 50 | Han | compliance | adherence and tolerability | life impact | delivery of care |
| 61 | Lam | Adherence | adherence and tolerability | life impact | delivery of care |
| 81 | Djabelkhir | attendance / acceptability / attrition rates | adherence and tolerability | life impact | delivery of care |
| 85 | Combourieu Donnezan | attendance | adherence and tolerability | life impact | delivery of care |
| 88 | Köbe | capsule intake (study adherence) | adherence and tolerability | life impact | delivery of care |
| 97 | Larouche | integration into daily life | adherence and tolerability | life impact | delivery of care |
| 102 | Eyre | attendance | adherence and tolerability | life impact | delivery of care |
| 109 | Doi | adherence to the dance and music programs | adherence and tolerability | life impact | delivery of care |
| 127 | Thomas | compliance | adherence and tolerability | life impact | delivery of care |
| 135 | Lü | compliance | adherence and tolerability | life impact | delivery of care |
| 5 | Baker | Adiposity | adiposity | physiological/clinical | metabolism and nutrition outcomes |
| 11 | Feldman | adverse events | adverse events | adverse events | adverse events |
| 12 | Winblad | adverse events | adverse events | adverse events | adverse events |
| 41 | Rovner | Rates of serious adverse events | adverse events | adverse events | adverse events |
| 52 | Zhang | adverse events | adverse events | adverse events | adverse events |
| 68 | Martin | adverse events | adverse events | adverse events | adverse events |
| 102 | Eyre | side effects | adverse events | adverse events | adverse events |
| 104 | Doody | adverse events | adverse events | adverse events | adverse events |
| 106 | Salloway | adverse events | adverse events | adverse events | adverse events |
| 108 | Yakoot | reported adverse effects | adverse events | adverse events | adverse events |
| 109 | Doi | adverse events | adverse events | adverse events | adverse events |
| 111 | Miao | Safety outcome measures / Adverse reactions | adverse events | adverse events | adverse events |
| 114 | Zhang | adverse events | adverse events | adverse events | adverse events |
| 115 | Dubois | adverse events | adverse events | adverse events | adverse events |
| 116 | Montero-Odasso | adverse events | adverse events | adverse events | adverse events |
| 118 | Devanand | adverse events | adverse events | adverse events | adverse events |
| 120 | Zhang | adverse events | adverse events | adverse events | adverse events |
| 127 | Thomas | adverse events | adverse events | adverse events | adverse events |
| 129 | ten Brinke | adverse effects | adverse events | adverse events | adverse events |
| 135 | Lü | adverse events | adverse events | adverse events | adverse events |
| 135 | Lü | adverse events | adverse events | adverse events | adverse events |
| 48 | Langoni | Aerobic conditioning | aerobic fitness | physiological/clinical | Respiratory, thoracic, and mediastinal outcomes |
| 85 | Combourieu Donnezan | VO2 max | aerobic fitness | physiological/clinical | Respiratory, thoracic, and mediastinal outcomes |
| 88 | Köbe | aerobic fitness | aerobic fitness | physiological/clinical | Respiratory, thoracic, and mediastinal outcomes |
| 16 | Vidovich | Alcohol Use | alcohol intake | physiological/clinical | general outcomes |
| 5 | Baker | Aß40 / Aß42 | amyloid-beta burden | physiological/clinical | nervous system outcomes |
| 63 | Bayer-Carter | CSF Aβ40 / CSF Aβ42 | amyloid-beta burden | physiological/clinical | nervous system outcomes |
| 127 | Thomas | Brain Aβ deposition | amyloid-beta burden | physiological/clinical | nervous system outcomes |
| 37 | Wenisch | Anxiety | anxiety | physiological/clinical | psychiatric outcomes |
| 45 | Belleville | anxiety symptoms | anxiety | physiological/clinical | psychiatric outcomes |
| 80 | Ciarmiello | anxiety | anxiety | physiological/clinical | psychiatric outcomes |
| 81 | Djabelkhir | anxiety | anxiety | physiological/clinical | psychiatric outcomes |
| 97 | Larouche | anxiety symptoms | anxiety | physiological/clinical | psychiatric outcomes |
| 113 | Savulich | anxiety | anxiety | physiological/clinical | psychiatric outcomes |
| 147 | Marciniak | anxiety level | anxiety | physiological/clinical | psychiatric outcomes |
| 102 | Eyre | apathy | apathy | life impact | emotional functioning/wellbeing |
| 113 | Savulich | apathy | apathy | life impact | emotional functioning/wellbeing |
| 149 | Padala | apathy | apathy | life impact | emotional functioning/wellbeing |
| 2 | Clare | associative learning | associative memory | life impact | cognitive functioning |
| 26 | Hampstead | associative memory / Object-location associations (OLA) accuracy | associative memory | life impact | cognitive functioning |
| 40 | Troyer | Objective memory ability / face-name learning | associative memory | life impact | cognitive functioning |
| 55 | Finn | verbal memory for associated word pairs | associative memory | life impact | cognitive functioning |
| 59 | Jean | face-name associations / memory | associative memory | life impact | cognitive functioning |
| 92 | Giuli | verbal memory and learning of word pairs | associative memory | life impact | cognitive functioning |
| 132 | Yogev-Seligmanna | associative memory encoding | associative memory | life impact | cognitive functioning |
| 6 | van Uffelen | Attention | attention | life impact | cognitive functioning |
| 12 | Winblad | sustained attention | attention | life impact | cognitive functioning |
| 14 | Belleville | Attentional control | attention | life impact | cognitive functioning |
| 16 | Vidovich | Attention | attention | life impact | cognitive functioning |
| 24 | Tsolaki | Attention | attention | life impact | cognitive functioning |
| 25 | Gagnon | attentional control | attention | life impact | cognitive functioning |
| 28 | Moro | Attention | attention | life impact | cognitive functioning |
| 32 | Rojas | Attention | attention | life impact | cognitive functioning |
| 42 | Park | Attention | attention | life impact | cognitive functioning |
| 53 | Jeong | attention | attention | life impact | cognitive functioning |
| 54 | Finn | Attentional set shifting / attention / Visual sustained attention | attention | life impact | cognitive functioning |
| 55 | Finn | Attention | attention | life impact | cognitive functioning |
| 58 | Fiatarone Singh | attention/speed | attention | life impact | cognitive functioning |
| 62 | Law | visual attention and task switching | attention | life impact | cognitive functioning |
| 67 | Tarnanas | attention | attention | life impact | cognitive functioning |
| 68 | Martin | attention and working memory | attention | life impact | cognitive functioning |
| 70 | Park | attention | attention | life impact | cognitive functioning |
| 74 | Bernini | attention | attention | life impact | cognitive functioning |
| 75 | Doshi | attention | attention | life impact | cognitive functioning |
| 79 | Yu | divided attention | attention | life impact | cognitive functioning |
| 83 | Lin | attention | attention | life impact | cognitive functioning |
| 91 | Hong | attention / selective attention | attention | life impact | cognitive functioning |
| 92 | Giuli | Assessment of selective and the sustained attention | attention | life impact | cognitive functioning |
| 95 | Park | attention | attention | life impact | cognitive functioning |
| 97 | Larouche | focused attention / networks of attention | attention | life impact | cognitive functioning |
| 100 | Zhu | attention | attention | life impact | cognitive functioning |
| 101 | Mahendran | attention | attention | life impact | cognitive functioning |
| 110 | Lazarou | attention | attention | life impact | cognitive functioning |
| 112 | Park | Attention | attention | life impact | cognitive functioning |
| 118 | Devanand | attention | attention | life impact | cognitive functioning |
| 143 | Hagovská | Attention | attention | life impact | cognitive functioning |
| 147 | Marciniak | attention | attention | life impact | cognitive functioning |
| 33 | Suzuki | Balance/postural stability | balance | life impact | physical functioning |
| 48 | Langoni | Balance | balance | life impact | physical functioning |
| 51 | Hagovská | Balance / dynamic balance / static balance | balance | life impact | physical functioning |
| 85 | Combourieu Donnezan | locomotion and dynamic balance ability | balance | life impact | physical functioning |
| 86 | Fogarty | balance | balance | life impact | physical functioning |
| 100 | Zhu | balance control | balance | life impact | physical functioning |
| 116 | Montero-Odasso | balance | balance | life impact | physical functioning |
| 134 | Khanthong | balance | balance | life impact | physical functioning |
| 135 | Lü | forward stability | balance | life impact | physical functioning |
| 152 | Lipardo | dynamic balance and functional mobility | balance | life impact | physical functioning |
| 10 | Petersen | Activities of Daily Living | basic activities of daily living | life impact | role functioning |
| 11 | Feldman | activities of daily living | basic activities of daily living | life impact | role functioning |
| 32 | Rojas | Activities of Daily Living | basic activities of daily living | life impact | role functioning |
| 35 | Kurz | Basic... ADL | basic activities of daily living | life impact | role functioning |
| 44 | Jeong | activities of daily living | basic activities of daily living | life impact | role functioning |
| 51 | Hagovská | Activities of daily living / ability to perform everyday activities | basic activities of daily living | life impact | role functioning |
| 62 | Law | daily self care | basic activities of daily living | life impact | role functioning |
| 71 | Brum | activities of daily living / functionality | basic activities of daily living | life impact | role functioning |
| 73 | Kim | basic activities of elderly individuals | basic activities of daily living | life impact | role functioning |
| 92 | Giuli | functional status of basic activities of daily living | basic activities of daily living | life impact | role functioning |
| 137 | Zhu | daily activities | basic activities of daily living | life impact | role functioning |
| 149 | Padala | Activities of daily living | basic activities of daily living | life impact | role functioning |
| 18 | Rozzini | behavioural and psychological symptoms (BPSD) | behavioural and psychological symptoms of dementia (BPSD) | physiological/clinical | psychiatric outcomes |
| 34 | Talassi | Behavioral assessment | behavioural disturbance | physiological/clinical | psychiatric outcomes |
| 104 | Doody | behavioral disturbances | behavioural disturbance | physiological/clinical | psychiatric outcomes |
| 23 | Rosen | Behavioral effects in the MRI / Training progress in experimental participants | bespoke outcome related to intervention | physiological/clinical | general outcomes |
| 60 | Smith | fMRI task performance | bespoke outcome related to intervention | physiological/clinical | general outcomes |
| 42 | Park | Arterial blood pressure | blood pressure | physiological/clinical | vascular outcomes |
| 42 | Park | Body height and weight | body composition | physiological/clinical | general outcomes |
| 53 | Jeong | Body composition | body composition | physiological/clinical | general outcomes |
| 88 | Köbe | anthropometry | body composition | physiological/clinical | general outcomes |
| 91 | Hong | Body composition | body composition | physiological/clinical | general outcomes |
| 2 | Clare | brain activation | brain activation during a cognitive task | physiological/clinical | nervous system outcomes |
| 15 | Liao | brain activation | brain activation during a cognitive task | physiological/clinical | nervous system outcomes |
| 38 | Belleville | Brain activation related to memory encoding / Brain activation related to memory retrieval | brain activation during a cognitive task | physiological/clinical | nervous system outcomes |
| 52 | Zhang | Brain activation | brain activation during a cognitive task | physiological/clinical | nervous system outcomes |
| 60 | Smith | memory fMRI activation | brain activation during a cognitive task | physiological/clinical | nervous system outcomes |
| 64 | Hampstead | fMRI activation changes… within memory-related areas | brain activation during a cognitive task | physiological/clinical | nervous system outcomes |
| 66 | Hampstead | Activation changes during encoding…fMRI / Activation changes during retrieval…fMRI | brain activation during a cognitive task | physiological/clinical | nervous system outcomes |
| 89 | Maffei | blood oxygen level dependent (BOLD) signal elicited by a cognitive task | brain activation during a cognitive task | physiological/clinical | nervous system outcomes |
| 114 | Zhang | brain activation | brain activation during a cognitive task | physiological/clinical | nervous system outcomes |
| 132 | Yogev-Seligmanna | functional MRI experiments of brain activation | brain activation during a cognitive task | physiological/clinical | nervous system outcomes |
| 23 | Rosen | brain function change | brain function | physiological/clinical | nervous system outcomes |
| 67 | Tarnanas | brain function | brain function | physiological/clinical | nervous system outcomes |
| 22 | Buschert | brain glucose metabolism | brain metabolism | physiological/clinical | nervous system outcomes |
| 70 | Park | brain metabolism...FDG PET | brain metabolism | physiological/clinical | nervous system outcomes |
| 80 | Ciarmiello | neuroimaging...F-FDG-PET acquisitions | brain metabolism | physiological/clinical | nervous system outcomes |
| 89 | Maffei | modifications in cerebral blood flow | brain perfusion and cerebrovascular health | physiological/clinical | nervous system outcomes |
| 127 | Thomas | brain perfusion / Cardiovascular hemodynamics / cerebrovascular hemodynamics | brain perfusion and cerebrovascular health | physiological/clinical | nervous system outcomes |
| 11 | Feldman | rate of volumetric changes | brain volume or structure | physiological/clinical | nervous system outcomes |
| 26 | Hampstead | medial temporal lobe volume | brain volume or structure | physiological/clinical | nervous system outcomes |
| 33 | Suzuki | brain volume | brain volume or structure | physiological/clinical | nervous system outcomes |
| 49 | Shimada | brain atrophy changes in medial temporal areas | brain volume or structure | physiological/clinical | nervous system outcomes |
| 58 | Fiatarone Singh | volume and thickness / hippocampal subfiled segmentation | brain volume or structure | physiological/clinical | nervous system outcomes |
| 79 | Yu | neuroimaging… whole brain analyses on CT | brain volume or structure | physiological/clinical | nervous system outcomes |
| 88 | Köbe | changes in gray matter volume | brain volume or structure | physiological/clinical | nervous system outcomes |
| 89 | Maffei | loss of grey and white matter in the cortex and the hippocampus with respect to T0 | brain volume or structure | physiological/clinical | nervous system outcomes |
| 93 | Zheng | gray matter volume | brain volume or structure | physiological/clinical | nervous system outcomes |
| 115 | Dubois | global cerebral volume / ventricular volume / hippocampal atrophy / left and right hippocampal volume | brain volume or structure | physiological/clinical | nervous system outcomes |
| 118 | Devanand | entorhinal cortical atrophy / MRI hyperintensities / hippocampal ... cortical atrophy | brain volume or structure | physiological/clinical | nervous system outcomes |
| 127 | Thomas | brain volume / white matter hyperintensity | brain volume or structure | physiological/clinical | nervous system outcomes |
| 129 | ten Brinke | Hippocampal volume | brain volume or structure | physiological/clinical | nervous system outcomes |
| 131 | Tao | gray matter, white matter, and cerebrospinal fluid | brain volume or structure | physiological/clinical | nervous system outcomes |
| 133 | Wells | total intracranial volume / Whole brain volume / bilateral hippocampal volumes / white matter | brain volume or structure | physiological/clinical | nervous system outcomes |
| 141 | Vermeij | Global brain atrophy / white matter lesions / hippocampal atrophy | brain volume or structure | physiological/clinical | nervous system outcomes |
| 5 | Baker | BDNF | brain-derived neurotrophic factor | physiological/clinical | nervous system outcomes |
| 33 | Suzuki | BDNF | brain-derived neurotrophic factor | physiological/clinical | nervous system outcomes |
| 44 | Jeong | Changes in BDNF levels | brain-derived neurotrophic factor | physiological/clinical | nervous system outcomes |
| 128 | Tsai | serum BDNF | brain-derived neurotrophic factor | physiological/clinical | nervous system outcomes |
| 136 | Sungkarat | plasma BDNF | brain-derived neurotrophic factor | physiological/clinical | nervous system outcomes |
| 5 | Baker | Cardiorespiratory Fitness | cardiorespiratory fitness | physiological/clinical | Respiratory, thoracic, and mediastinal outcomes |
| 58 | Fiatarone Singh | cardiorespiratory fitness | cardiorespiratory fitness | physiological/clinical | Respiratory, thoracic, and mediastinal outcomes |
| 60 | Smith | cardiorespiratory fitness | cardiorespiratory fitness | physiological/clinical | Respiratory, thoracic, and mediastinal outcomes |
| 88 | Köbe | cardiorespiratory fitness | cardiorespiratory fitness | physiological/clinical | Respiratory, thoracic, and mediastinal outcomes |
| 127 | Thomas | cardiorespiratory fitness | cardiorespiratory fitness | physiological/clinical | Respiratory, thoracic, and mediastinal outcomes |
| 128 | Tsai | cardiorespiratory fitness | cardiorespiratory fitness | physiological/clinical | Respiratory, thoracic, and mediastinal outcomes |
| 132 | Yogev-Seligmanna | cardiorespiratory fitness | cardiorespiratory fitness | physiological/clinical | Respiratory, thoracic, and mediastinal outcomes |
| 5 | Baker | cardiovascular outcomes | cardiovascular health | physiological/clinical | vascular outcomes |
| 88 | Köbe | Intima Media Thickness of the distal right and left common carotid artery | cardiovascular health | physiological/clinical | vascular outcomes |
| 127 | Thomas | cardiovascular fitness | cardiovascular health | physiological/clinical | vascular outcomes |
| 4 | Greenaway | caregiver burden | caregiver burden | resource use | societal/carer burden |
| 30 | Greenaway | caregiver burden | caregiver burden | resource use | societal/carer burden |
| 36 | Joosten-Weyn Banningh | burden of caregiving | caregiver burden | resource use | societal/carer burden |
| 142 | Bahar-Fuchs | caregiver burden | caregiver burden | resource use | societal/carer burden |
| 149 | Padala | caregiver burden | caregiver burden | resource use | societal/carer burden |
| 5 | Baker | cholesterol | cholesterol | physiological/clinical | metabolism and nutrition outcomes |
| 33 | Suzuki | total cholesterol | cholesterol | physiological/clinical | metabolism and nutrition outcomes |
| 63 | Bayer-Carter | Cholesterol levels / Low-density lipoprotein cholesterol (LDL-C) concentrations | cholesterol | physiological/clinical | metabolism and nutrition outcomes |
| 88 | Köbe | lipid / high-to-low density lipoprotein (HDL-to-LDL) ratio / total cholesterol / serum levels of triacylglycerides | cholesterol | physiological/clinical | metabolism and nutrition outcomes |
| 55 | Finn | cognitive flexibility | cognitive flexibility | life impact | cognitive functioning |
| 51 | Hagovská | dual cognitive task | cognitive/perceptual load | life impact | cognitive functioning |
| 90 | Busse | concentration | concentration | life impact | cognitive functioning |
| 135 | Lü | perceived confidence in performing balance-related activities | confidence in balance | life impact | physical functioning |
| 39 | Hampstead | memory confidence | confidence in cognition and memory | life impact | cognitive functioning |
| 92 | Giuli | subjective confidence in own cognition and memory | confidence in cognition and memory | life impact | cognitive functioning |
| 99 | Bae | conversation time | conversational ability | life impact | social functioning |
| 97 | Larouche | coping strategies | coping strategies | life impact | emotional functioning/wellbeing |
| 5 | Baker | cortisol | cortisol | physiological/clinical | endocrine outcomes |
| 97 | Larouche | cortisol awakening response | cortisol awakening response | physiological/clinical | endocrine outcomes |
| 88 | Köbe | high-sensitive C-reactive protein / inflammation | C-reactive protein | physiological/clinical | immune system outcomes |
| 147 | Marciniak | Plasma analysis… CRP | C-reactive protein | physiological/clinical | immune system outcomes |
| 88 | Köbe | IL-6 / inflammation / tumor necrosis factor-alpha (TNF-α) | cytokines | physiological/clinical | immune system outcomes |
| 128 | Tsai | Cytokines | cytokines | physiological/clinical | immune system outcomes |
| 136 | Sungkarat | cytokines / plasma IL-10 levels / plasma TNF-α | cytokines | physiological/clinical | immune system outcomes |
| 147 | Marciniak | Plasma analysis...IL-6 / TNF-alpha | cytokines | physiological/clinical | immune system outcomes |
| 5 | Baker | declarative memory | declarative memory | life impact | cognitive functioning |
| 11 | Feldman | clinical dementia rating | dementia severity | physiological/clinical | nervous system outcomes |
| 32 | Rojas | Stage level | dementia severity | physiological/clinical | nervous system outcomes |
| 92 | Giuli | overall dementia severity | dementia severity | physiological/clinical | nervous system outcomes |
| 104 | Doody | dementia severity | dementia severity | physiological/clinical | nervous system outcomes |
| 9 | Chiu | Depression | depression | physiological/clinical | psychiatric outcomes |
| 11 | Feldman | depression | depression | physiological/clinical | psychiatric outcomes |
| 18 | Rozzini | depressive symptoms | depression | physiological/clinical | psychiatric outcomes |
| 19 | Barnes | Depressive symptomatology | depression | physiological/clinical | psychiatric outcomes |
| 35 | Kurz | Depressive symptoms | depression | physiological/clinical | psychiatric outcomes |
| 36 | Joosten-Weyn Banningh | depressive symptoms / Distress | depression | physiological/clinical | psychiatric outcomes |
| 37 | Wenisch | depression symptoms | depression | physiological/clinical | psychiatric outcomes |
| 42 | Park | Symptoms of depression | depression | physiological/clinical | psychiatric outcomes |
| 44 | Jeong | depression | depression | physiological/clinical | psychiatric outcomes |
| 45 | Belleville | depressive symptoms | depression | physiological/clinical | psychiatric outcomes |
| 47 | Han | Depressive mood | depression | physiological/clinical | psychiatric outcomes |
| 48 | Langoni | Depressive symptoms | depression | physiological/clinical | psychiatric outcomes |
| 53 | Jeong | depression | depression | physiological/clinical | psychiatric outcomes |
| 57 | González-Palau | Depression | depression | physiological/clinical | psychiatric outcomes |
| 60 | Smith | depression symptoms | depression | physiological/clinical | psychiatric outcomes |
| 61 | Lam | depressive symptoms | depression | physiological/clinical | psychiatric outcomes |
| 70 | Park | depressive mood | depression | physiological/clinical | psychiatric outcomes |
| 71 | Brum | depression | depression | physiological/clinical | psychiatric outcomes |
| 73 | Kim | depressive symptomatology | depression | physiological/clinical | psychiatric outcomes |
| 80 | Ciarmiello | depression | depression | physiological/clinical | psychiatric outcomes |
| 94 | Emsaki | depression | depression | physiological/clinical | psychiatric outcomes |
| 95 | Park | Depressive symptoms | depression | physiological/clinical | psychiatric outcomes |
| 96 | Masika | depression | depression | physiological/clinical | psychiatric outcomes |
| 97 | Larouche | depressive symptoms | depression | physiological/clinical | psychiatric outcomes |
| 98 | Klainin-Yobas | depression | depression | physiological/clinical | psychiatric outcomes |
| 99 | Bae | depressive symptoms | depression | physiological/clinical | psychiatric outcomes |
| 100 | Zhu | subjective experience of depression | depression | physiological/clinical | psychiatric outcomes |
| 107 | Lee | Depressive symptomatology | depression | physiological/clinical | psychiatric outcomes |
| 113 | Savulich | depression | depression | physiological/clinical | psychiatric outcomes |
| 117 | Reynolds | recurrence of major depression | depression | physiological/clinical | psychiatric outcomes |
| 118 | Devanand | depression | depression | physiological/clinical | psychiatric outcomes |
| 119 | Özenli | depression rate | depression | physiological/clinical | psychiatric outcomes |
| 128 | Tsai | depression | depression | physiological/clinical | psychiatric outcomes |
| 139 | Kim | depressive symptoms | depression | physiological/clinical | psychiatric outcomes |
| 147 | Marciniak | depressive symptoms | depression | physiological/clinical | psychiatric outcomes |
| 124 | Liu | subjective "deqi" sensations | deqi sensations (subjective sensation from acupuncture) | physiological/clinical | general outcomes |
| 3 | Joosten-Weyn Banningh | Distress | distress | life impact | emotional functioning/wellbeing |
| 91 | Hong | EEG frequency | EEG frequency | physiological/clinical | nervous system outcomes |
| 103 | Thapa | delta/alpha ratio; EEG brain power ratio-theta/beta ratio; theta/alpha ratio | EEG frequency | physiological/clinical | nervous system outcomes |
| 128 | Tsai | Neurophysiological Performance | EEG signatures | physiological/clinical | nervous system outcomes |
| 3 | Joosten-Weyn Banningh | word-list learning | episodic memory | life impact | cognitive functioning |
| 12 | Winblad | immediate and delayed (i.e., 5-minute) recall | episodic memory | life impact | cognitive functioning |
| 14 | Belleville | episodic memory | episodic memory | life impact | cognitive functioning |
| 15 | Liao | Verbal memory | episodic memory | life impact | cognitive functioning |
| 21 | Jean | recall | episodic memory | life impact | cognitive functioning |
| 22 | Buschert | story memory and story recall | episodic memory | life impact | cognitive functioning |
| 27 | Herrera | Verbal memory / Visual memory | episodic memory | life impact | cognitive functioning |
| 29 | Carretti | Long-term memory | episodic memory | life impact | cognitive functioning |
| 32 | Rojas | Episodic memory | episodic memory | life impact | cognitive functioning |
| 34 | Talassi | Episodic memory | episodic memory | life impact | cognitive functioning |
| 35 | Kurz | Verbal episodic memory / Non-verbal/visual episodic memory | episodic memory | life impact | cognitive functioning |
| 38 | Belleville | delayed recall | episodic memory | life impact | cognitive functioning |
| 43 | Jirayucharoensak | Pattern recognition memory / Pattern Delayed Matching to Sample | episodic memory | life impact | cognitive functioning |
| 49 | Shimada | verbal memory | episodic memory | life impact | cognitive functioning |
| 52 | Zhang | Episodic memory | episodic memory | life impact | cognitive functioning |
| 54 | Finn | Visual recognition / Visual learning | episodic memory | life impact | cognitive functioning |
| 61 | Lam | episodic memory | episodic memory | life impact | cognitive functioning |
| 62 | Law | Verbal Learning | episodic memory | life impact | cognitive functioning |
| 63 | Bayer-Carter | immediate and delayed memory | episodic memory | life impact | cognitive functioning |
| 65 | Akhtar | free recall | episodic memory | life impact | cognitive functioning |
| 68 | Martin | verbal memory | episodic memory | life impact | cognitive functioning |
| 69 | Poptsi | verbal memory / visual memory / verbal episodic memory | episodic memory | life impact | cognitive functioning |
| 70 | Park | memory | episodic memory | life impact | cognitive functioning |
| 74 | Bernini | episodic long-term memory | episodic memory | life impact | cognitive functioning |
| 75 | Doshi | delayed memory | episodic memory | life impact | cognitive functioning |
| 76 | Duff | list learning / list recall / list recognition / Story recall / Story memory | episodic memory | life impact | cognitive functioning |
| 79 | Yu | immediate and delayed recall | episodic memory | life impact | cognitive functioning |
| 80 | Ciarmiello | Episodic memory | episodic memory | life impact | cognitive functioning |
| 81 | Djabelkhir | episodic memory | episodic memory | life impact | cognitive functioning |
| 87 | Turriziani | recognition memory | episodic memory | life impact | cognitive functioning |
| 92 | Giuli | memory of prose | episodic memory | life impact | cognitive functioning |
| 94 | Emsaki | Autobiographical memory / visual memory | episodic memory | life impact | cognitive functioning |
| 97 | Larouche | episodic memory | episodic memory | life impact | cognitive functioning |
| 99 | Bae | delayed recall | episodic memory | life impact | cognitive functioning |
| 101 | Mahendran | delayed recall / recognition trial (memory) / list learning | episodic memory | life impact | cognitive functioning |
| 102 | Eyre | Verbal memory | episodic memory | life impact | cognitive functioning |
| 105 | de Jager | episodic memory | episodic memory | life impact | cognitive functioning |
| 110 | Lazarou | learning and long-term memory; story direct and delayed recall | episodic memory | life impact | cognitive functioning |
| 112 | Park | memory / word-list | episodic memory | life impact | cognitive functioning |
| 113 | Savulich | episodic memory / visuospatial learning and memory / recent, long-term visuospatial memory | episodic memory | life impact | cognitive functioning |
| 114 | Zhang | episodic memory | episodic memory | life impact | cognitive functioning |
| 117 | Reynolds | Delayed Memory | episodic memory | life impact | cognitive functioning |
| 118 | Devanand | nonverbal learning and memory / episodic verbal memory | episodic memory | life impact | cognitive functioning |
| 120 | Zhang | episodic memory | episodic memory | life impact | cognitive functioning |
| 127 | Thomas | verbal learning and episodic memory | episodic memory | life impact | cognitive functioning |
| 129 | ten Brinke | Verbal memory and learning | episodic memory | life impact | cognitive functioning |
| 132 | Yogev-Seligmanna | Immediate and delayed verbal learning and memory | episodic memory | life impact | cognitive functioning |
| 141 | Vermeij | episodic memory | episodic memory | life impact | cognitive functioning |
| 147 | Marciniak | visual learning | episodic memory | life impact | cognitive functioning |
| 9 | Chiu | Plasma erythrocyte membrane fatty acid compositions | erythrocytes fatty acid membrane composition | physiological/clinical | metabolism and nutrition outcomes |
| 88 | Köbe | erythrocyte membrane fatty acid | erythrocytes fatty acid membrane composition | physiological/clinical | metabolism and nutrition outcomes |
| 55 | Finn | Everyday Cognitive function | everyday cognitive function | life impact | cognitive functioning |
| 141 | Vermeij | cognitive problems in daily life | everyday cognitive function | life impact | cognitive functioning |
| 44 | Jeong | everyday memory | everyday memory | life impact | cognitive functioning |
| 94 | Emsaki | retrospective and prospective memory slips in everyday life | everyday memory | life impact | cognitive functioning |
| 144 | Marra | everyday memory | everyday memory | life impact | cognitive functioning |
| 5 | Baker | Executive Function | executive function | life impact | cognitive functioning |
| 6 | van Uffelen | Executive function | executive function | life impact | cognitive functioning |
| 15 | Liao | executive function | executive function | life impact | cognitive functioning |
| 16 | Vidovich | Executive Functions | executive function | life impact | cognitive functioning |
| 19 | Barnes | Executive/Attention | executive function | life impact | cognitive functioning |
| 22 | Buschert | attentional and additionally executive function | executive function | life impact | cognitive functioning |
| 24 | Tsolaki | Executive function | executive function | life impact | cognitive functioning |
| 26 | Hampstead | executive functioning | executive function | life impact | cognitive functioning |
| 28 | Moro | Executive functions | executive function | life impact | cognitive functioning |
| 32 | Rojas | Executive functions | executive function | life impact | cognitive functioning |
| 33 | Suzuki | Executive function | executive function | life impact | cognitive functioning |
| 37 | Wenisch | Executive functioning | executive function | life impact | cognitive functioning |
| 42 | Park | Executive function | executive function | life impact | cognitive functioning |
| 44 | Jeong | Executive function | executive function | life impact | cognitive functioning |
| 46 | Barban | Executive functions | executive function | life impact | cognitive functioning |
| 49 | Shimada | executive function | executive function | life impact | cognitive functioning |
| 52 | Zhang | Executive function | executive function | life impact | cognitive functioning |
| 57 | González-Palau | sustained attention and executive functions | executive function | life impact | cognitive functioning |
| 58 | Fiatarone Singh | executive function | executive function | life impact | cognitive functioning |
| 63 | Bayer-Carter | Executive function | executive function | life impact | cognitive functioning |
| 67 | Tarnanas | executive function | executive function | life impact | cognitive functioning |
| 69 | Poptsi | executive function | executive function | life impact | cognitive functioning |
| 70 | Park | executive function | executive function | life impact | cognitive functioning |
| 74 | Bernini | logical-executive functions | executive function | life impact | cognitive functioning |
| 80 | Ciarmiello | logical-deductive skill | executive function | life impact | cognitive functioning |
| 81 | Djabelkhir | executive functions | executive function | life impact | cognitive functioning |
| 83 | Lin | cognitive control | executive function | life impact | cognitive functioning |
| 85 | Combourieu Donnezan | executive function | executive function | life impact | cognitive functioning |
| 95 | Park | frontal lobe executive function | executive function | life impact | cognitive functioning |
| 97 | Larouche | executive functioning | executive function | life impact | cognitive functioning |
| 99 | Bae | attention and executive function | executive function | life impact | cognitive functioning |
| 100 | Zhu | executive function | executive function | life impact | cognitive functioning |
| 101 | Mahendran | executive function | executive function | life impact | cognitive functioning |
| 102 | Eyre | executive functions | executive function | life impact | cognitive functioning |
| 103 | Thapa | prefrontal cognitive function | executive function | life impact | cognitive functioning |
| 105 | de Jager | executive function | executive function | life impact | cognitive functioning |
| 107 | Lee | executive function and attention | executive function | life impact | cognitive functioning |
| 110 | Lazarou | executive function | executive function | life impact | cognitive functioning |
| 111 | Miao | executive function | executive function | life impact | cognitive functioning |
| 112 | Park | Executive function | executive function | life impact | cognitive functioning |
| 114 | Zhang | executive function | executive function | life impact | cognitive functioning |
| 117 | Reynolds | Executive Function | executive function | life impact | cognitive functioning |
| 118 | Devanand | executive function | executive function | life impact | cognitive functioning |
| 120 | Zhang | executive function | executive function | life impact | cognitive functioning |
| 127 | Thomas | EXECUTIVE FUNCTION | executive function | life impact | cognitive functioning |
| 128 | Tsai | executive control / global switching cost | executive function | life impact | cognitive functioning |
| 134 | Khanthong | executive function | executive function | life impact | cognitive functioning |
| 135 | Lü | executive function | executive function | life impact | cognitive functioning |
| 136 | Sungkarat | executive function | executive function | life impact | cognitive functioning |
| 144 | Marra | Executive function tests (frontal lobe tasks) | executive function | life impact | cognitive functioning |
| 147 | Marciniak | executive functions | executive function | life impact | cognitive functioning |
| 149 | Padala | Executive Function | executive function | life impact | cognitive functioning |
| 51 | Hagovská | fear of falling | falls | life impact | physical functioning |
| 116 | Montero-Odasso | falls / cognitive-motor interaction and fall risk | falls | life impact | physical functioning |
| 136 | Sungkarat | fall risk | falls | life impact | physical functioning |
| 152 | Lipardo | fall incidence/ Overall risk for falls | falls | life impact | physical functioning |
| 128 | Tsai | serum FGF-2 | fibroblast growth factor-2 | physiological/clinical | general outcomes |
| 29 | Carretti | Fluid intelligence | fluid intelligence | life impact | cognitive functioning |
| 4 | Greenaway | functional ability | functional ability | life impact | role functioning |
| 12 | Winblad | global functioning | functional ability | life impact | role functioning |
| 15 | Liao | functional status | functional ability | life impact | role functioning |
| 16 | Vidovich | Functional Ability | functional ability | life impact | role functioning |
| 18 | Rozzini | functional status | functional ability | life impact | role functioning |
| 24 | Tsolaki | Daily life activities | functional ability | life impact | role functioning |
| 34 | Talassi | Functional assessment | functional ability | life impact | role functioning |
| 51 | Hagovská | FUNCTIONAL ACTIVITIES | functional ability | life impact | role functioning |
| 69 | Poptsi | general functional performance / caregiver’s opinion about daily function of people with cognitive disorders | functional ability | life impact | role functioning |
| 78 | Pantoni | Functional status | functional ability | life impact | role functioning |
| 81 | Djabelkhir | global functioning | functional ability | life impact | role functioning |
| 106 | Salloway | global function | functional ability | life impact | role functioning |
| 110 | Lazarou | daily functionality | functional ability | life impact | role functioning |
| 118 | Devanand | Functional Activities | functional ability | life impact | role functioning |
| 143 | Hagovská | Functional Activities | functional ability | life impact | role functioning |
| 149 | Padala | Functional status | functional ability | life impact | role functioning |
| 64 | Hampstead | connectivity within memory-related areas | functional connectivity | physiological/clinical | nervous system outcomes |
| 120 | Zhang | functional connectivity within DMN | functional connectivity | physiological/clinical | nervous system outcomes |
| 121 | Tan | resting brain networks | functional connectivity | physiological/clinical | nervous system outcomes |
| 123 | Li | Hippocampal Functional Connectivity | functional connectivity | physiological/clinical | nervous system outcomes |
| 131 | Tao | resting state functional MRI / functional connectivity | functional connectivity | physiological/clinical | nervous system outcomes |
| 133 | Wells | functional connectivity | functional connectivity | physiological/clinical | nervous system outcomes |
| 140 | Styliadis | resting state functional connectivity | functional connectivity | physiological/clinical | nervous system outcomes |
| 145 | Cui | DMN functional connectivity | functional connectivity | physiological/clinical | nervous system outcomes |
| 148 | Fam | Functional connectivity | functional connectivity | physiological/clinical | nervous system outcomes |
| 150 | Yuan | Functional connectivity | functional connectivity | physiological/clinical | nervous system outcomes |
| 154 | Liu | Functional connectivity density | functional connectivity | physiological/clinical | nervous system outcomes |
| 48 | Langoni | Mobility | functional mobility | life impact | physical functioning |
| 103 | Thapa | mobility | functional mobility | life impact | physical functioning |
| 135 | Lü | functional mobility | functional mobility | life impact | physical functioning |
| 146 | Bisbe | Functional mobility | functional mobility | life impact | physical functioning |
| 15 | Liao | Gait performance | gait | life impact | physical functioning |
| 33 | Suzuki | Gait analysis | gait | life impact | physical functioning |
| 86 | Fogarty | gait velocity, stride time, strime time variability | gait | life impact | physical functioning |
| 116 | Montero-Odasso | Gait performance / gait variability | gait | life impact | physical functioning |
| 1 | Londos | cognition | global cognition | life impact | cognitive functioning |
| 3 | Joosten-Weyn Banningh | Cognitive impairment | global cognition | life impact | cognitive functioning |
| 7 | Lodgson | global cognitive status | global cognition | life impact | cognitive functioning |
| 9 | Chiu | global cognitive function / global clinical function | global cognition | life impact | cognitive functioning |
| 10 | Petersen | cognitive function | global cognition | life impact | cognitive functioning |
| 11 | Feldman | cognitive function | global cognition | life impact | cognitive functioning |
| 12 | Winblad | cognitive status | global cognition | life impact | cognitive functioning |
| 13 | Rapp | Cognitive function | global cognition | life impact | cognitive functioning |
| 15 | Liao | global cognition | global cognition | life impact | cognitive functioning |
| 16 | Vidovich | Global Cognition | global cognition | life impact | cognitive functioning |
| 17 | Zhang | cognitive function | global cognition | life impact | cognitive functioning |
| 18 | Rozzini | cognitive functions | global cognition | life impact | cognitive functioning |
| 19 | Barnes | global cognitive function | global cognition | life impact | cognitive functioning |
| 21 | Jean | global cognitive screening / cognitive functioning | global cognition | life impact | cognitive functioning |
| 22 | Buschert | change in cognitive function | global cognition | life impact | cognitive functioning |
| 24 | Tsolaki | General cognitive performance | global cognition | life impact | cognitive functioning |
| 30 | Greenaway | cognition | global cognition | life impact | cognitive functioning |
| 31 | Olchik | Cognitive | global cognition | life impact | cognitive functioning |
| 32 | Rojas | Global cognitive level | global cognition | life impact | cognitive functioning |
| 33 | Suzuki | cognition | global cognition | life impact | cognitive functioning |
| 34 | Talassi | Cognitive functions | global cognition | life impact | cognitive functioning |
| 36 | Joosten-Weyn Banningh | cognitive changes during the intervention period | global cognition | life impact | cognitive functioning |
| 41 | Rovner | Cognition | global cognition | life impact | cognitive functioning |
| 42 | Park | Global cognitive function / Detailed neurocognitive function | global cognition | life impact | cognitive functioning |
| 44 | Jeong | Global cognitive function | global cognition | life impact | cognitive functioning |
| 45 | Belleville | Cognitive measures | global cognition | life impact | cognitive functioning |
| 46 | Barban | Global cognition | global cognition | life impact | cognitive functioning |
| 47 | Han | Global cognition | global cognition | life impact | cognitive functioning |
| 48 | Langoni | Cognition | global cognition | life impact | cognitive functioning |
| 49 | Shimada | general cognitive status and memory function | global cognition | life impact | cognitive functioning |
| 50 | Han | cognitive functions | global cognition | life impact | cognitive functioning |
| 51 | Hagovská | examination of basic cognitive functions | global cognition | life impact | cognitive functioning |
| 52 | Zhang | General mental status | global cognition | life impact | cognitive functioning |
| 56 | Sugano | Cognitive function | global cognition | life impact | cognitive functioning |
| 57 | González-Palau | cognitive functions | global cognition | life impact | cognitive functioning |
| 58 | Fiatarone Singh | global cognitive function | global cognition | life impact | cognitive functioning |
| 59 | Jean | global cognition | global cognition | life impact | cognitive functioning |
| 61 | Lam | global cognitive ability / function / cognitive outcomes | global cognition | life impact | cognitive functioning |
| 62 | Law | Cognitive Status | global cognition | life impact | cognitive functioning |
| 68 | Martin | cognitive outcomes | global cognition | life impact | cognitive functioning |
| 69 | Poptsi | general cognitive function | global cognition | life impact | cognitive functioning |
| 70 | Park | overall cognitive function | global cognition | life impact | cognitive functioning |
| 71 | Brum | cognitive decline | global cognition | life impact | cognitive functioning |
| 72 | Hwang | Cognition | global cognition | life impact | cognitive functioning |
| 73 | Kim | improvement in cognitive function | global cognition | life impact | cognitive functioning |
| 74 | Bernini | global cognition | global cognition | life impact | cognitive functioning |
| 75 | Doshi | global cognition | global cognition | life impact | cognitive functioning |
| 77 | Li | multiple cognitive functions | global cognition | life impact | cognitive functioning |
| 78 | Pantoni | Improvement in cognitive performance | global cognition | life impact | cognitive functioning |
| 80 | Ciarmiello | global cognitive status | global cognition | life impact | cognitive functioning |
| 82 | Hyer | cognitive | global cognition | life impact | cognitive functioning |
| 84 | Li | cognitive function | global cognition | life impact | cognitive functioning |
| 86 | Fogarty | cognitive function | global cognition | life impact | cognitive functioning |
| 88 | Köbe | global cognition | global cognition | life impact | cognitive functioning |
| 89 | Maffei | extent of cognitive decline | global cognition | life impact | cognitive functioning |
| 90 | Busse | general cognition | global cognition | life impact | cognitive functioning |
| 92 | Giuli | Global cognitive abilities | global cognition | life impact | cognitive functioning |
| 93 | Zheng | global cognitive function | global cognition | life impact | cognitive functioning |
| 95 | Park | General cognition / neurocognitive function | global cognition | life impact | cognitive functioning |
| 96 | Masika | brief cognitive assessment | global cognition | life impact | cognitive functioning |
| 97 | Larouche | general cognitive functioning | global cognition | life impact | cognitive functioning |
| 98 | Klainin-Yobas | cognitive function | global cognition | life impact | cognitive functioning |
| 99 | Bae | Global cognitive status | global cognition | life impact | cognitive functioning |
| 100 | Zhu | cognitive function | global cognition | life impact | cognitive functioning |
| 103 | Thapa | global cognitive function | global cognition | life impact | cognitive functioning |
| 104 | Doody | cognition | global cognition | life impact | cognitive functioning |
| 105 | de Jager | global cognition | global cognition | life impact | cognitive functioning |
| 106 | Salloway | Cognitive function | global cognition | life impact | cognitive functioning |
| 108 | Yakoot | Cognitive Impairment | global cognition | life impact | cognitive functioning |
| 109 | Doi | general cognitive status | global cognition | life impact | cognitive functioning |
| 110 | Lazarou | general cognitive function | global cognition | life impact | cognitive functioning |
| 111 | Miao | global cognition | global cognition | life impact | cognitive functioning |
| 114 | Zhang | general mental status | global cognition | life impact | cognitive functioning |
| 115 | Dubois | neuropsychological and  cognitive evaluation | global cognition | life impact | cognitive functioning |
| 116 | Montero-Odasso | cognition | global cognition | life impact | cognitive functioning |
| 117 | Reynolds | Neuropsychological functioning/global cognition | global cognition | life impact | cognitive functioning |
| 118 | Devanand | several cognitive domains | global cognition | life impact | cognitive functioning |
| 119 | Özenli | cognitive state | global cognition | life impact | cognitive functioning |
| 120 | Zhang | general mental status | global cognition | life impact | cognitive functioning |
| 121 | Tan | cognition / cognitive performance | global cognition | life impact | cognitive functioning |
| 123 | Li | Cognitive Assessment | global cognition | life impact | cognitive functioning |
| 127 | Thomas | cognition / executive function, visuospatial abilities, motor programming, attention, and concentration | global cognition | life impact | cognitive functioning |
| 131 | Tao | cognitive screening | global cognition | life impact | cognitive functioning |
| 132 | Yogev-Seligmanna | general cognitive screening / different cognitive domains | global cognition | life impact | cognitive functioning |
| 133 | Wells | global cognitive measure | global cognition | life impact | cognitive functioning |
| 134 | Khanthong | global cognitive tests | global cognition | life impact | cognitive functioning |
| 135 | Lü | global cognitive function | global cognition | life impact | cognitive functioning |
| 137 | Zhu | cognitive function | global cognition | life impact | cognitive functioning |
| 138 | Sun | cognitive functions | global cognition | life impact | cognitive functioning |
| 139 | Kim | cognitive dysfunction / cognitive decline | global cognition | life impact | cognitive functioning |
| 140 | Styliadis | Cognitive function | global cognition | life impact | cognitive functioning |
| 142 | Bahar-Fuchs | global cognitive function | global cognition | life impact | cognitive functioning |
| 143 | Hagovská | Detailed cognitive profile | global cognition | life impact | cognitive functioning |
| 145 | Cui | global cognitive function | global cognition | life impact | cognitive functioning |
| 146 | Bisbe | Global cognition | global cognition | life impact | cognitive functioning |
| 148 | Fam | neurocognitive function | global cognition | life impact | cognitive functioning |
| 149 | Padala | Cognition | global cognition | life impact | cognitive functioning |
| 150 | Yuan | overall cognitive function | global cognition | life impact | cognitive functioning |
| 151 | Siu | global cognitive assessment | global cognition | life impact | cognitive functioning |
| 154 | Liu | cognitive function | global cognition | life impact | cognitive functioning |
| 2 | Clare | goal-oriented rehabilitation / goal attainment | goal-oriented rehabilitation | life impact | personal circumstances |
| 33 | Suzuki | hemoglobin A1c | haemoglobin A1c | physiological/clinical | metabolism and nutrition outcomes |
| 88 | Köbe | HbA1c / glucose | haemoglobin A1c | physiological/clinical | metabolism and nutrition outcomes |
| 33 | Suzuki | Hand grip strength | handgrip strength | physiological/clinical | musculoskeletal and connective tissue outcomes |
| 42 | Park | Grip strength | handgrip strength | physiological/clinical | musculoskeletal and connective tissue outcomes |
| 99 | Bae | maximum hand grip strength | handgrip strength | physiological/clinical | musculoskeletal and connective tissue outcomes |
| 103 | Thapa | Handgrip strength (HGS) of the non-dominant hand | handgrip strength | physiological/clinical | musculoskeletal and connective tissue outcomes |
| 7 | Lodgson | self-rated health status | health-related quality of life | life impact | global quality of life |
| 40 | Troyer | illness-induced disruptions to lifestyle and activities that negativelyimpinge on quality of life | health-related quality of life | life impact | global quality of life |
| 73 | Kim | health-related quality of life | health-related quality of life | life impact | global quality of life |
| 112 | Park | health-related quality of life | health-related quality of life | life impact | global quality of life |
| 139 | Kim | health-related quality of life | health-related quality of life | life impact | global quality of life |
| 3 | Joosten-Weyn Banningh | helplessness | helplessness | life impact | emotional functioning/wellbeing |
| 36 | Joosten-Weyn Banningh | Helplessness | helplessness | life impact | emotional functioning/wellbeing |
| 88 | Köbe | total homocysteine | homocysteine | physiological/clinical | metabolism and nutrition outcomes |
| 29 | Carretti | Short-term memory | immediate (short-term) memory | life impact | cognitive functioning |
| 38 | Belleville | immediate recall | immediate (short-term) memory | life impact | cognitive functioning |
| 75 | Doshi | immediate memory | immediate (short-term) memory | life impact | cognitive functioning |
| 80 | Ciarmiello | Short-term memory impairment | immediate (short-term) memory | life impact | cognitive functioning |
| 92 | Giuli | Immediate verbal recall | immediate (short-term) memory | life impact | cognitive functioning |
| 99 | Bae | immediate recognition | immediate (short-term) memory | life impact | cognitive functioning |
| 100 | Zhu | short-term verbal memory | immediate (short-term) memory | life impact | cognitive functioning |
| 104 | Doody | short-term memory and attention | immediate (short-term) memory | life impact | cognitive functioning |
| 135 | Lü | immediate memory and attention | immediate (short-term) memory | life impact | cognitive functioning |
| 145 | Cui | short-term memory | immediate (short-term) memory | life impact | cognitive functioning |
| 43 | Jirayucharoensak | Rapid Visual information processing | information processing | life impact | cognitive functioning |
| 132 | Yogev-Seligmanna | complex information processing | information processing | life impact | cognitive functioning |
| 15 | Liao | inhibition | inhibitory control | life impact | cognitive functioning |
| 29 | Carretti | Inhibition | inhibitory control | life impact | cognitive functioning |
| 91 | Hong | response inhibition | inhibitory control | life impact | cognitive functioning |
| 12 | Winblad | basic and social functioning | instrumental activities of daily living | life impact | role functioning |
| 25 | Gagnon | difficulty of different combinations of everyday activities | instrumental activities of daily living | life impact | role functioning |
| 30 | Greenaway | IADL | instrumental activities of daily living | life impact | role functioning |
| 35 | Kurz | instrumental ADL | instrumental activities of daily living | life impact | role functioning |
| 41 | Rovner | Performance-based function | instrumental activities of daily living | life impact | role functioning |
| 45 | Belleville | difficulties in performing complex activities of daily living | instrumental activities of daily living | life impact | role functioning |
| 56 | Sugano | IADL | instrumental activities of daily living | life impact | role functioning |
| 58 | Fiatarone Singh | mental capacity to perform daily tasks | instrumental activities of daily living | life impact | role functioning |
| 61 | Lam | instrumental activity of daily living | instrumental activities of daily living | life impact | role functioning |
| 68 | Martin | instrumental activities of daily living | instrumental activities of daily living | life impact | role functioning |
| 73 | Kim | complex activities representing instrumental self-maintenance and social behavior | instrumental activities of daily living | life impact | role functioning |
| 76 | Duff | difficulty with IADLs / daily functioning | instrumental activities of daily living | life impact | role functioning |
| 83 | Lin | performance speed and accuracy of multiple IADL domains | instrumental activities of daily living | life impact | role functioning |
| 92 | Giuli | Assessment of functional status of IADL | instrumental activities of daily living | life impact | role functioning |
| 100 | Zhu | IADL | instrumental activities of daily living | life impact | role functioning |
| 111 | Miao | ability of daily living (ADL) | instrumental activities of daily living | life impact | role functioning |
| 117 | Reynolds | Instrumental Activities of Daily Living (IADL) | instrumental activities of daily living | life impact | role functioning |
| 142 | Bahar-Fuchs | Independence in day-to-day activities | instrumental activities of daily living | life impact | role functioning |
| 143 | Hagovská | Individual's self-sufficiency | instrumental activities of daily living | life impact | role functioning |
| 151 | Siu | IALD | instrumental activities of daily living | life impact | role functioning |
| 5 | Baker | insulin | insulin | physiological/clinical | metabolism and nutrition outcomes |
| 88 | Köbe | insulin | insulin | physiological/clinical | metabolism and nutrition outcomes |
| 128 | Tsai | insulin | insulin | physiological/clinical | metabolism and nutrition outcomes |
| 5 | Baker | glucose metabolism / insulin sensitivity | insulin sensitivity | physiological/clinical | metabolism and nutrition outcomes |
| 63 | Bayer-Carter | Insulin resistance | insulin sensitivity | physiological/clinical | metabolism and nutrition outcomes |
| 5 | Baker | IGF-I | insulin-like growth factor 1 | physiological/clinical | endocrine outcomes |
| 88 | Köbe | Insulin-like growth factor 1 | insulin-like growth factor 1 | physiological/clinical | endocrine outcomes |
| 128 | Tsai | serum IGF-1 | insulin-like growth factor 1 | physiological/clinical | endocrine outcomes |
| 19 | Barnes | Language | language | life impact | cognitive functioning |
| 24 | Tsolaki | Language | language | life impact | cognitive functioning |
| 32 | Rojas | Language | language | life impact | cognitive functioning |
| 42 | Park | Verbal function | language | life impact | cognitive functioning |
| 52 | Zhang | Language ability | language | life impact | cognitive functioning |
| 69 | Poptsi | language | language | life impact | cognitive functioning |
| 70 | Park | language | language | life impact | cognitive functioning |
| 75 | Doshi | language | language | life impact | cognitive functioning |
| 95 | Park | verbal function | language | life impact | cognitive functioning |
| 97 | Larouche | language | language | life impact | cognitive functioning |
| 111 | Miao | language function | language | life impact | cognitive functioning |
| 114 | Zhang | language ability | language | life impact | cognitive functioning |
| 117 | Reynolds | Language Function | language | life impact | cognitive functioning |
| 120 | Zhang | language | language | life impact | cognitive functioning |
| 88 | Köbe | leptin | leptin | physiological/clinical | metabolism and nutrition outcomes |
| 128 | Tsai | leptin | leptin | physiological/clinical | metabolism and nutrition outcomes |
| 33 | Suzuki | logical memory function | logical memory | life impact | cognitive functioning |
| 44 | Jeong | Logical memory | logical memory | life impact | cognitive functioning |
| 49 | Shimada | logical memory function | logical memory | life impact | cognitive functioning |
| 57 | González-Palau | Logical memory | logical memory | life impact | cognitive functioning |
| 100 | Zhu | logical memory / memory performance | logical memory | life impact | cognitive functioning |
| 144 | Marra | logical memory | logical memory | life impact | cognitive functioning |
| 147 | Marciniak | Post-Intervention Practice | long-term maintenance of intervention | life impact | delivery of care |
| 2 | Clare | memory | memory | life impact | cognitive functioning |
| 5 | Baker | memory / visual memory | memory | life impact | cognitive functioning |
| 6 | van Uffelen | Memory | memory | life impact | cognitive functioning |
| 13 | Rapp | Memory performance | memory | life impact | cognitive functioning |
| 16 | Vidovich | Memory | memory | life impact | cognitive functioning |
| 19 | Barnes | Memory | memory | life impact | cognitive functioning |
| 20 | Kinsella | Memory (dis)ability | memory | life impact | cognitive functioning |
| 21 | Jean | everyday memory functioning | memory | life impact | cognitive functioning |
| 24 | Tsolaki | Visual or verbal memory | memory | life impact | cognitive functioning |
| 28 | Moro | Memory | memory | life impact | cognitive functioning |
| 37 | Wenisch | Memory | memory | life impact | cognitive functioning |
| 39 | Hampstead | memory | memory | life impact | cognitive functioning |
| 40 | Troyer | Self-reported everyday memory functioning / objective memory ability | memory | life impact | cognitive functioning |
| 46 | Barban | Memory | memory | life impact | cognitive functioning |
| 47 | Han | memory | memory | life impact | cognitive functioning |
| 57 | González-Palau | memory | memory | life impact | cognitive functioning |
| 58 | Fiatarone Singh | memory | memory | life impact | cognitive functioning |
| 59 | Jean | everyday memory functioning | memory | life impact | cognitive functioning |
| 64 | Hampstead | Memory performance | memory | life impact | cognitive functioning |
| 67 | Tarnanas | memory | memory | life impact | cognitive functioning |
| 86 | Fogarty | memory abilities and frequency of memory slips | memory | life impact | cognitive functioning |
| 90 | Busse | memory | memory | life impact | cognitive functioning |
| 91 | Hong | memory | memory | life impact | cognitive functioning |
| 93 | Zheng | memory | memory | life impact | cognitive functioning |
| 104 | Doody | overall change in patient memory | memory | life impact | cognitive functioning |
| 107 | Lee | memory | memory | life impact | cognitive functioning |
| 109 | Doi | memory | memory | life impact | cognitive functioning |
| 111 | Miao | memory | memory | life impact | cognitive functioning |
| 113 | Savulich | information originally learned that was retained / learning | memory | life impact | cognitive functioning |
| 119 | Özenli | memory | memory | life impact | cognitive functioning |
| 132 | Yogev-Seligmanna | memory | memory | life impact | cognitive functioning |
| 134 | Khanthong | memory function | memory | life impact | cognitive functioning |
| 136 | Sungkarat | Memory | memory | life impact | cognitive functioning |
| 142 | Bahar-Fuchs | Learning and memory | memory | life impact | cognitive functioning |
| 144 | Marra | short-term auditory-verbal memory, rate of learning, learning, and retrieval | memory | life impact | cognitive functioning |
| 147 | Marciniak | memory / learning | memory | life impact | cognitive functioning |
| 20 | Kinsella | Knowledge and use of memory strategies | memory strategies | life impact | cognitive functioning |
| 40 | Troyer | Memory-strategy knowledge and behaviour | memory strategies | life impact | cognitive functioning |
| 44 | Jeong | memory strategies | memory strategies | life impact | cognitive functioning |
| 45 | Belleville | how frequently participants use memory strategies in everyday life | memory strategies | life impact | cognitive functioning |
| 86 | Fogarty | memory strategies | memory strategies | life impact | cognitive functioning |
| 65 | Akhtar | metacognitive sensitivity | meta-cognitive ability | life impact | cognitive functioning |
| 142 | Bahar-Fuchs | Meta-memory | meta-cognitive ability | life impact | cognitive functioning |
| 75 | Doshi | awareness and attention to present events and experiences | mindfulness | life impact | emotional functioning/wellbeing |
| 97 | Larouche | mindfulness attitudes | mindfulness | life impact | emotional functioning/wellbeing |
| 2 | Clare | mood | mood | life impact | emotional functioning/wellbeing |
| 13 | Rapp | Mood | mood | life impact | emotional functioning/wellbeing |
| 16 | Vidovich | mood | mood | life impact | emotional functioning/wellbeing |
| 22 | Buschert | Mood | mood | life impact | emotional functioning/wellbeing |
| 30 | Greenaway | mood | mood | life impact | emotional functioning/wellbeing |
| 54 | Finn | Mood | mood | life impact | emotional functioning/wellbeing |
| 55 | Finn | Mood | mood | life impact | emotional functioning/wellbeing |
| 68 | Martin | mood | mood | life impact | emotional functioning/wellbeing |
| 75 | Doshi | mood | mood | life impact | emotional functioning/wellbeing |
| 81 | Djabelkhir | mood | mood | life impact | emotional functioning/wellbeing |
| 88 | Köbe | mood | mood | life impact | emotional functioning/wellbeing |
| 92 | Giuli | mood status and depression | mood | life impact | emotional functioning/wellbeing |
| 102 | Eyre | mood | mood | life impact | emotional functioning/wellbeing |
| 110 | Lazarou | mood | mood | life impact | emotional functioning/wellbeing |
| 142 | Bahar-Fuchs | mood and wellbeing | mood | life impact | emotional functioning/wellbeing |
| 48 | Langoni | Muscle endurance of the lower limbs | muscle endurance (lower limbs) | physiological/clinical | musculoskeletal and connective tissue outcomes |
| 58 | Fiatarone Singh | strength | muscular strength | physiological/clinical | musculoskeletal and connective tissue outcomes |
| 90 | Busse | variation in muscle strength | muscular strength | physiological/clinical | musculoskeletal and connective tissue outcomes |
| 134 | Khanthong | strength | muscular strength | physiological/clinical | musculoskeletal and connective tissue outcomes |
| 152 | Lipardo | lower extremity muscle strength | muscular strength | physiological/clinical | musculoskeletal and connective tissue outcomes |
| 11 | Feldman | neuropsychiatric… outcomes | neuropsychiatric symptoms | physiological/clinical | psychiatric outcomes |
| 32 | Rojas | Neuropsychiatric | neuropsychiatric symptoms | physiological/clinical | psychiatric outcomes |
| 44 | Jeong | neuropsychiatric symptoms | neuropsychiatric symptoms | physiological/clinical | psychiatric outcomes |
| 146 | Bisbe | psycho-affective symptoms | neuropsychiatric symptoms | physiological/clinical | psychiatric outcomes |
| 153 | Cintoli | Informant ratings of neuropsychiatric symptoms | neuropsychiatric symptoms | physiological/clinical | psychiatric outcomes |
| 23 | Rosen | Neuropsychological evaluation | neuropsychology | life impact | cognitive functioning |
| 26 | Hampstead | Neuropsychological Status | neuropsychology | life impact | cognitive functioning |
| 53 | Jeong | Neuropsychological cognitive | neuropsychology | life impact | cognitive functioning |
| 60 | Smith | neuropsychological testing | neuropsychology | life impact | cognitive functioning |
| 88 | Köbe | neuropsychological assessment | neuropsychology | life impact | cognitive functioning |
| 130 | Lin | Neuropsychological | neuropsychology | life impact | cognitive functioning |
| 146 | Bisbe | Neuropsychological assessment | neuropsychology | life impact | cognitive functioning |
| 109 | Doi | nonmemory cognitive functions | non-memory cognition | life impact | cognitive functioning |
| 142 | Bahar-Fuchs | Non memory, global cognition | non-memory cognition | life impact | cognitive functioning |
| 99 | Bae | number of outdoor activities | number of outdoor activities | life impact | physical functioning |
| 1 | Londos | occupational performance | occupational performance | life impact | role functioning |
| 88 | Köbe | omega-3 FA intake | omega-3 intake | physiological/clinical | metabolism and nutrition outcomes |
| 92 | Giuli | Orientation and spatial attention | orientation | life impact | cognitive functioning |
| 16 | Vidovich | enagement in leisure activities | participation in activities | life impact | role functioning |
| 41 | Rovner | Participation in Activities | participation in activities | life impact | role functioning |
| 13 | Rapp | Perceived control over memory | perceived control over memory | life impact | cognitive functioning |
| 54 | Finn | Perceived control over memory | perceived control over memory | life impact | cognitive functioning |
| 147 | Marciniak | immunophenotyping, peripheral blood monocytes | peripheral blood monocytes | physiological/clinical | immune system outcomes |
| 147 | Marciniak | Phagocytic activity | phagocytic activity | physiological/clinical | immune system outcomes |
| 7 | Lodgson | physical activity | physical activity levels | life impact | physical functioning |
| 16 | Vidovich | weekly physical activity | physical activity levels | life impact | physical functioning |
| 42 | Park | Exercise | physical activity levels | life impact | physical functioning |
| 49 | Shimada | physical activity/Mobility | physical activity levels | life impact | physical functioning |
| 53 | Jeong | Habitual Physical Activity | physical activity levels | life impact | physical functioning |
| 58 | Fiatarone Singh | physical activity | physical activity levels | life impact | physical functioning |
| 60 | Smith | exercise | physical activity levels | life impact | physical functioning |
| 86 | Fogarty | levels of everyday physical activity | physical activity levels | life impact | physical functioning |
| 88 | Köbe | physical activity and other lifestyle habits | physical activity levels | life impact | physical functioning |
| 99 | Bae | Physical activity measure / step count | physical activity levels | life impact | physical functioning |
| 109 | Doi | objective measures of physical activity | physical activity levels | life impact | physical functioning |
| 127 | Thomas | total amount of exercise performed by the AE group over 12 months | physical activity levels | life impact | physical functioning |
| 146 | Bisbe | Habitual Physical Activity | physical activity levels | life impact | physical functioning |
| 85 | Combourieu Donnezan | intensity | physical fitness | life impact | physical functioning |
| 88 | Köbe | changes in physical fitness / maximum effort of the elderly patients | physical fitness | life impact | physical functioning |
| 91 | Hong | Physical fitness | physical fitness | life impact | physical functioning |
| 128 | Tsai | senior functional physical fitness | physical fitness | life impact | physical functioning |
| 42 | Park | Physical function | physical functioning | life impact | physical functioning |
| 56 | Sugano | Physical function | physical functioning | life impact | physical functioning |
| 60 | Smith | physical function | physical functioning | life impact | physical functioning |
| 84 | Li | physical function | physical functioning | life impact | physical functioning |
| 103 | Thapa | physical function | physical functioning | life impact | physical functioning |
| 139 | Kim | physical function | physical functioning | life impact | physical functioning |
| 146 | Bisbe | Physical functioning assessment | physical functioning | life impact | physical functioning |
| 16 | Vidovich | Health Issues | physical health | physiological/clinical | general outcomes |
| 86 | Fogarty | ratings of physical health | physical health | physiological/clinical | general outcomes |
| 5 | Baker | platelet factor 4 | platelet factor 4 | physiological/clinical | immune system outcomes |
| 1 | Londos | cognitive processing speed | processing speed | life impact | cognitive functioning |
| 6 | van Uffelen | information processing speed | processing speed | life impact | cognitive functioning |
| 16 | Vidovich | Proc. Speed | processing speed | life impact | cognitive functioning |
| 29 | Carretti | Processing speed | processing speed | life impact | cognitive functioning |
| 33 | Suzuki | Processing speed | processing speed | life impact | cognitive functioning |
| 52 | Zhang | Processing speed | processing speed | life impact | cognitive functioning |
| 53 | Jeong | processing speed | processing speed | life impact | cognitive functioning |
| 54 | Finn | processing speed | processing speed | life impact | cognitive functioning |
| 68 | Martin | processing speed | processing speed | life impact | cognitive functioning |
| 74 | Bernini | processing speed | processing speed | life impact | cognitive functioning |
| 83 | Lin | visual processing speed | processing speed | life impact | cognitive functioning |
| 97 | Larouche | Processing speed | processing speed | life impact | cognitive functioning |
| 99 | Bae | Processing speed | processing speed | life impact | cognitive functioning |
| 100 | Zhu | processing speed | processing speed | life impact | cognitive functioning |
| 117 | Reynolds | Information Processing Speed | processing speed | life impact | cognitive functioning |
| 118 | Devanand | processing speed | processing speed | life impact | cognitive functioning |
| 120 | Zhang | processing speed | processing speed | life impact | cognitive functioning |
| 3 | Joosten-Weyn Joosten-Weyn Banningh | Dementia | progression to dementia (incidence) | physiological/clinical | nervous system outcomes |
| 12 | Winblad | progression to dementia | progression to dementia (incidence) | physiological/clinical | nervous system outcomes |
| 22 | Buschert | conversion to AD | progression to dementia (incidence) | physiological/clinical | nervous system outcomes |
| 78 | Pantoni | Reduction of the risk of transition to dementia | progression to dementia (incidence) | physiological/clinical | nervous system outcomes |
| 113 | Savulich | screen for dementia | progression to dementia (incidence) | physiological/clinical | nervous system outcomes |
| 20 | Kinsella | Prospective Memory Performance | prospective memory | life impact | cognitive functioning |
| 20 | Kinsella | Psychological well-being | psychological wellbeing | life impact | emotional functioning/wellbeing |
| 45 | Belleville | Psychological health | psychological wellbeing | life impact | emotional functioning/wellbeing |
| 101 | Mahendran | psychological wellbeing | psychological wellbeing | life impact | emotional functioning/wellbeing |
| 8 | Yan | motor control | psycho-motor skills | life impact | physical functioning |
| 63 | Bayer-Carter | Motor speed | psycho-motor skills | life impact | physical functioning |
| 107 | Lee | Psychomotor speed | psycho-motor skills | life impact | physical functioning |
| 147 | Marciniak | psychomotor speed | psycho-motor skills | life impact | physical functioning |
| 1 | Londos | quality of life | quality of life | life impact | global quality of life |
| 11 | Feldman | quality of life | quality of life | life impact | global quality of life |
| 14 | Belleville | quality of life | quality of life | life impact | global quality of life |
| 16 | Vidovich | quality of life | quality of life | life impact | global quality of life |
| 22 | Buschert | Quality of life | quality of life | life impact | global quality of life |
| 30 | Greenaway | quality of life | quality of life | life impact | global quality of life |
| 32 | Rojas | Quality of Life | quality of life | life impact | global quality of life |
| 44 | Jeong | QOL | quality of life | life impact | global quality of life |
| 68 | Martin | quality of life | quality of life | life impact | global quality of life |
| 72 | Hwang | quality of life | quality of life | life impact | global quality of life |
| 78 | Pantoni | Quality of life | quality of life | life impact | global quality of life |
| 81 | Djabelkhir | quality of life | quality of life | life impact | global quality of life |
| 97 | Larouche | General quality of life (gQOL) | quality of life | life impact | global quality of life |
| 100 | Zhu | quality of life | quality of life | life impact | global quality of life |
| 146 | Bisbe | quality of life | quality of life | life impact | global quality of life |
| 153 | Cintoli | Quality of Life | quality of life | life impact | global quality of life |
| 33 | Suzuki | Reaction time | reaction time | life impact | physical functioning |
| 51 | Hagovská | personal tempo / psychomotor and personal speed | reaction time | life impact | physical functioning |
| 113 | Savulich | ability to react as quickly as possible to basic stimuli | reaction time | life impact | physical functioning |
| 16 | Vidovich | social support / Social activity | relationships with others | life impact | social functioning |
| 56 | Sugano | Social and human relationships | relationships with others | life impact | social functioning |
| 102 | Eyre | Psychological resilience to stress | resilience to stress | life impact | emotional functioning/wellbeing |
| 58 | Fiatarone Singh | spontaneous functional changes in the brain | resting state or spontaneous brain activity | physiological/clinical | nervous system outcomes |
| 77 | Li | spontaneous brain activities... resting-state fMRI, fractional ALFF (fALFF) approach | resting state or spontaneous brain activity | physiological/clinical | nervous system outcomes |
| 78 | Pantoni | improvement in long-term brain activity | resting state or spontaneous brain activity | physiological/clinical | nervous system outcomes |
| 83 | Lin | neuroimaging… MRI | resting state or spontaneous brain activity | physiological/clinical | nervous system outcomes |
| 100 | Zhu | brain spontaneous activity change / brain neural activity | resting state or spontaneous brain activity | physiological/clinical | nervous system outcomes |
| 122 | Jia | resting-state brain activity | resting state or spontaneous brain activity | physiological/clinical | nervous system outcomes |
| 124 | Liu | resting state brain activity | resting state or spontaneous brain activity | physiological/clinical | nervous system outcomes |
| 125 | Shan | brain activity | resting state or spontaneous brain activity | physiological/clinical | nervous system outcomes |
| 126 | Wang | regional brain activities | resting state or spontaneous brain activity | physiological/clinical | nervous system outcomes |
| 140 | Styliadis | resting state change of brain activity | resting state or spontaneous brain activity | physiological/clinical | nervous system outcomes |
| 97 | Larouche | changes in ruminations | rumination | physiological/clinical | psychiatric outcomes |
| 72 | Hwang | satisfaction | satisfaction with outcomes | life impact | delivery of care |
| 82 | Hyer | satisfaction | satisfaction with outcomes | life impact | delivery of care |
| 102 | Eyre | satisfaction with the outcomes | satisfaction with outcomes | life impact | delivery of care |
| 3 | Joosten-Weyn Banningh | Satisfaction with the partner relationship | satisfaction with relationships | life impact | social functioning |
| 50 | Han | level of satisfaction | satisfaction with the intervention | life impact | delivery of care |
| 81 | Djabelkhir | motivation | satisfaction with the intervention | life impact | delivery of care |
| 85 | Combourieu Donnezan | appreciation | satisfaction with the intervention | life impact | delivery of care |
| 113 | Savulich | enjoyment | satisfaction with the intervention | life impact | delivery of care |
| 113 | Savulich | level of self-confidence | self-confidence | life impact | emotional functioning/wellbeing |
| 30 | Greenaway | self-efficacy | self-efficacy | life impact | emotional functioning/wellbeing |
| 21 | Jean | individual's beliefs and emotions regarding self-perception | self-esteem | life impact | emotional functioning/wellbeing |
| 81 | Djabelkhir | global self-worth | self-esteem | life impact | emotional functioning/wellbeing |
| 67 | Tarnanas | language and semantic memory | semantic memory | life impact | cognitive functioning |
| 97 | Larouche | semantic memory | semantic memory | life impact | cognitive functioning |
| 105 | de Jager | semantic memory | semantic memory | life impact | cognitive functioning |
| 142 | Bahar-Fuchs | Olfactory Discrimination, Taste Identification, Olfactory Recognition | sensory functioning | physiological/clinical | general outcomes |
| 9 | Chiu | disease severity and progression of illness | severity of symptoms | physiological/clinical | nervous system outcomes |
| 10 | Petersen | Global Deterioration | severity of symptoms | physiological/clinical | nervous system outcomes |
| 11 | Feldman | global deterioration | severity of symptoms | physiological/clinical | nervous system outcomes |
| 149 | Padala | improvement / clinical global impression (CGI)) outcomes | severity of symptoms | physiological/clinical | nervous system outcomes |
| 101 | Mahendran | Sleep quality | sleep quality | physiological/clinical | general outcomes |
| 128 | Tsai | social participation | social participation | life impact | social functioning |
| 147 | Marciniak | Spiritual Well-Being | spiritual wellbeing | life impact | cognitive functioning |
| 92 | Giuli | perceived psychological stress | stress | life impact | emotional functioning/wellbeing |
| 97 | Larouche | Psychological stress | stress | life impact | emotional functioning/wellbeing |
| 61 | Lam | Subjective cognitive complaints | subjective cognitive functioning | life impact | cognitive functioning |
| 68 | Martin | subjective cognitive functioning | subjective cognitive functioning | life impact | cognitive functioning |
| 70 | Park | Subjective cognitive assessment | subjective cognitive functioning | life impact | cognitive functioning |
| 97 | Larouche | cognitive complaints / perception of memory and attention difficulties | subjective cognitive functioning | life impact | cognitive functioning |
| 104 | Doody | subjective cognitive problems | subjective cognitive functioning | life impact | cognitive functioning |
| 3 | Joosten-Weyn Banningh | Alertness to memory failure and other changes | subjective memory | life impact | cognitive functioning |
| 13 | Rapp | Perceptions of memory impairment | subjective memory | life impact | cognitive functioning |
| 14 | Belleville | subjective memory | subjective memory | life impact | cognitive functioning |
| 16 | Vidovich | perception of memory | subjective memory | life impact | cognitive functioning |
| 21 | Jean | subjective memory | subjective memory | life impact | cognitive functioning |
| 40 | Troyer | Satisfaction with memory ability | subjective memory | life impact | cognitive functioning |
| 45 | Belleville | memory complaint | subjective memory | life impact | cognitive functioning |
| 47 | Han | Subjective memory | subjective memory | life impact | cognitive functioning |
| 54 | Finn | Subjective memory impairment | subjective memory | life impact | cognitive functioning |
| 81 | Djabelkhir | subjective memory complaints | subjective memory | life impact | cognitive functioning |
| 90 | Busse | Memory Complaints | subjective memory | life impact | cognitive functioning |
| 92 | Giuli | subjective memory complaints | subjective memory | life impact | cognitive functioning |
| 113 | Savulich | self-rated memory ability | subjective memory | life impact | cognitive functioning |
| 142 | Bahar-Fuchs | memory awareness | subjective memory | life impact | cognitive functioning |
| 63 | Bayer-Carter | CSF tau | tau burden | physiological/clinical | nervous system outcomes |
| 101 | Mahendran | telomere length | telomere length | physiological/clinical | general outcomes |
| 10 | Petersen | time to the development of possible or probable Alzheimer’s disease dementia | time to progression to dementia | physiological/clinical | nervous system outcomes |
| 11 | Feldman | time to the clinical diagnosis of AD | time to progression to dementia | physiological/clinical | nervous system outcomes |
| 111 | Miao | syndrome differentiation | Traditional Chinese medicine symptoms of dementia | physiological/clinical | general outcomes |
| 130 | Lin | CM dementia syndromes / Xin (Heart) and Shen (Kidney) deficiency | Traditional Chinese medicine symptoms of dementia | physiological/clinical | general outcomes |
| 40 | Troyer | participants' thoughts about the relationship between lifestyle factors (i.e., stress/relaxation, nutrition, physical activity, and participation in cognitively-stimulating activities) and memory | understanding of factors affecting memory | life impact | cognitive functioning |
| 33 | Suzuki | VEGFR1 | vascular endothelial growth factor | physiological/clinical | vascular outcomes |
| 128 | Tsai | serum VEGF | vascular endothelial growth factor | physiological/clinical | vascular outcomes |
| 14 | Belleville | Verbal fluency | verbal fluency | life impact | cognitive functioning |
| 31 | Olchik | Verbal fluency | verbal fluency | life impact | cognitive functioning |
| 33 | Suzuki | Verbal fluency | verbal fluency | life impact | cognitive functioning |
| 34 | Talassi | verbal fluency | verbal fluency | life impact | cognitive functioning |
| 37 | Wenisch | Verbal Fluency | verbal fluency | life impact | cognitive functioning |
| 58 | Fiatarone Singh | verbal fluency | verbal fluency | life impact | cognitive functioning |
| 62 | Law | verbal fluency | verbal fluency | life impact | cognitive functioning |
| 79 | Yu | verbal fluency | verbal fluency | life impact | cognitive functioning |
| 83 | Lin | verbal fluency | verbal fluency | life impact | cognitive functioning |
| 88 | Köbe | verbal fluency (semantic and phonemic) | verbal fluency | life impact | cognitive functioning |
| 91 | Hong | Semantic/phonemic fluency | verbal fluency | life impact | cognitive functioning |
| 92 | Giuli | phonemic fluency / semantic fluency | verbal fluency | life impact | cognitive functioning |
| 110 | Lazarou | Verbal Fluency | verbal fluency | life impact | cognitive functioning |
| 118 | Devanand | category fluency | verbal fluency | life impact | cognitive functioning |
| 132 | Yogev-Seligmanna | verbal fluency | verbal fluency | life impact | cognitive functioning |
| 24 | Tsolaki | Visual-constructive abilities | visuoconstructional abilities | life impact | cognitive functioning |
| 67 | Tarnanas | praxis and visuospatial skills | visuoconstructional abilities | life impact | cognitive functioning |
| 69 | Poptsi | visual constructive abilities | visuoconstructional abilities | life impact | cognitive functioning |
| 71 | Brum | visuo-constructional capacity | visuoconstructional abilities | life impact | cognitive functioning |
| 75 | Doshi | visuospatial/constructional | visuoconstructional abilities | life impact | cognitive functioning |
| 80 | Ciarmiello | visuoconstruction | visuoconstructional abilities | life impact | cognitive functioning |
| 97 | Larouche | visuo-constructive abilities | visuoconstructional abilities | life impact | cognitive functioning |
| 32 | Rojas | Visuospatial | visuospatial abilities | life impact | cognitive functioning |
| 51 | Hagovská | attention, psychomotor speed and visual search capability | visuospatial abilities | life impact | cognitive functioning |
| 52 | Zhang | Visuospatial ability | visuospatial abilities | life impact | cognitive functioning |
| 79 | Yu | visuospatial functions | visuospatial abilities | life impact | cognitive functioning |
| 97 | Larouche | visuo-perception | visuospatial abilities | life impact | cognitive functioning |
| 101 | Mahendran | Visuospatial abilities | visuospatial abilities | life impact | cognitive functioning |
| 102 | Eyre | visual-spatial skills and visual memory | visuospatial abilities | life impact | cognitive functioning |
| 107 | Lee | visuospatial skills | visuospatial abilities | life impact | cognitive functioning |
| 110 | Lazarou | visuospatial ability | visuospatial abilities | life impact | cognitive functioning |
| 112 | Park | Visual spatial abiliy | visuospatial abilities | life impact | cognitive functioning |
| 114 | Zhang | visuospatial ability | visuospatial abilities | life impact | cognitive functioning |
| 117 | Reynolds | Visuospatial Function | visuospatial abilities | life impact | cognitive functioning |
| 118 | Devanand | visuospatial skills | visuospatial abilities | life impact | cognitive functioning |
| 120 | Zhang | visuo-spatial ability | visuospatial abilities | life impact | cognitive functioning |
| 132 | Yogev-Seligmanna | visio-spatial abilities and memory | visuospatial abilities | life impact | cognitive functioning |
| 136 | Sungkarat | visuospatial ability | visuospatial abilities | life impact | cognitive functioning |
| 88 | Köbe | folate / B vitamin metabolism / vitamin B12 | vitamin B metabolism | physiological/clinical | metabolism and nutrition outcomes |
| 33 | Suzuki | Walking speed | walking | life impact | physical functioning |
| 42 | Park | walking speed | walking | life impact | physical functioning |
| 85 | Combourieu Donnezan | Single-task walking / dual task walking condition / complex walking | walking | life impact | physical functioning |
| 99 | Bae | walking speed | walking | life impact | physical functioning |
| 134 | Khanthong | speed | walking | life impact | physical functioning |
| 152 | Lipardo | walking speed | walking | life impact | physical functioning |
| 3 | Joosten-Weyn Joosten-Weyn Banningh | general well-being | wellbeing | life impact | emotional functioning/wellbeing |
| 25 | Gagnon | subjective personal well-being | wellbeing | life impact | emotional functioning/wellbeing |
| 36 | Joosten-Weyn Banningh | wellbeing | wellbeing | life impact | emotional functioning/wellbeing |
| 113 | Savulich | desire to continue | willingness to continue with intervention | life impact | delivery of care |
| 118 | Devanand | confrontation naming for language | word-finding | life impact | cognitive functioning |
| 29 | Carretti | Verbal working memory / Visuospatial WM | working memory | life impact | cognitive functioning |
| 42 | Park | Working memory | working memory | life impact | cognitive functioning |
| 43 | Jirayucharoensak | Spatial working memory / Spatial Span Length | working memory | life impact | cognitive functioning |
| 44 | Jeong | Working memory | working memory | life impact | cognitive functioning |
| 54 | Finn | Visual working memory | working memory | life impact | cognitive functioning |
| 55 | Finn | Visual Working memory | working memory | life impact | cognitive functioning |
| 57 | González-Palau | Attention and working memory | working memory | life impact | cognitive functioning |
| 61 | Lam | attention and working memory | working memory | life impact | cognitive functioning |
| 74 | Bernini | working memory | working memory | life impact | cognitive functioning |
| 76 | Duff | Digit Span | working memory | life impact | cognitive functioning |
| 79 | Yu | working memory span | working memory | life impact | cognitive functioning |
| 81 | Djabelkhir | working memory | working memory | life impact | cognitive functioning |
| 83 | Lin | working memory | working memory | life impact | cognitive functioning |
| 90 | Busse | working memory | working memory | life impact | cognitive functioning |
| 91 | Hong | working memory | working memory | life impact | cognitive functioning |
| 92 | Giuli | working memory | working memory | life impact | cognitive functioning |
| 94 | Emsaki | working and auditory memories | working memory | life impact | cognitive functioning |
| 99 | Bae | Spatial working memory | working memory | life impact | cognitive functioning |
| 101 | Mahendran | working memory | working memory | life impact | cognitive functioning |
| 114 | Zhang | working memory | working memory | life impact | cognitive functioning |
| 120 | Zhang | working memory | working memory | life impact | cognitive functioning |
| 128 | Tsai | working memory span | working memory | life impact | cognitive functioning |
| 141 | Vermeij | near-transfer working-memory / far-transfer working-memory | working memory | life impact | cognitive functioning |
| 144 | Marra | working memory | working memory | life impact | cognitive functioning |
| 147 | Marciniak | working memory | working memory | life impact | cognitive functioning |
