# Supplementary material for: Outcomes reported in interventional studies for mild cognitive impairment: a scoping umbrella review to inform core outcome set development
Source: Alzheimers Dement. 2026 Sep 5;22(9):e71800. doi: 10.1002/alz.71800 (PMC13546403; doi:10.1002/alz.71800)
Supplement: Supplementary file 4 [file ALZ-22-e71800-s003.docx]

Supplementary Materials – Appendix E

Most frequently reported outcome measures for each outcome reported

| **Outcome** | **Number of outcome measures*** | **Most frequently used outcome measures: N of studies** |
| --- | --- | --- |
| **Cognitive outcomes** | | |
| global cognition | 51 | **1. Mini-Mental State Examination (MMSE): 58** 2. Montreal Cognitive Assessment (MoCA): 22 3. ADAS-Cog: 21 |
| executive function | 21 | **1. Trail Making Test (TMT) or Color Trails Test: 31** 2. Stroop Colour and Word Test: 19 3. Verbal Fluency Test: 14 |
| episodic memory | 24 | **1. Auditory Verbal Learning Test (AVLT, incl. Rey's, California): 22** 2. Word List Recall & Recognition (incl. subtests from RBANS, Côte-des-Neiges Computerized Memory Battery): 13 3. Complex Figure Reproduction (incl. Rey-Osterrieth Complex Figure test and WMS): 12 |
| memory | 22 | **1. Auditory Verbal Learning Test (AVLT, incl. Rey's, California): 11** 2. Story recall (incl. Logical Memory Test from WMS or WAIS): 9 3. Word List Recall & Recognition (incl. subtests from MoCA, CERAD): 6 |
| attention | 25 | **1. Trail Making Test (TMT) or Color Trails Test: 9** 2. Digit Span: 8 3. Stroop Colour and Word Test: 4 |
| working memory | 13 | **1. Digit Span: 13** 2. Spatial Span: 6 3. N-back test: 3 |
| processing speed | 9 | **1. Digit Symbol Substitution Test (incl. WAIS): 11** 2. Trail Making Test (TMT): 5 All other measures were used by only one study. |
| visuospatial abilities | 6 | **1. WAIS Block Design Test: 7** 2. Rey-Osterrieth Complex Figure test: 5 3. Clock Drawing Test: 4 |
| verbal fluency | 1 | **Verbal Fluency Tests** (letter and/or category) or specifically the Controlled Oral Word Association Test (COWAT) were used for all studies. |
| subjective memory | 11 | **1. Memory Functioning Questionnaire: 3** 2. *Questionnaire d'Auto-Evaluation de la Memoire:* 2 All other measures were used by only one study. |
| language | 7 | **1. Verbal Fluency Tests (incl. COWAT): 8** 2. Boston Naming Test: 6 3. Boston Diagnostic Aphasia Examination: 2 |
| immediate (short-term) memory | 6 | **1. Digit Span: 5** All other measures were used by only one study. |
| neuropsychology | 14 | **1. RBANS, AVLT, MMSE, and Trail Making Tests** were each used by two studies.  All other tests were used by only one study. |
| associative memory | 3 | **1. Face-name associative learning task: 3** 2. Learning word pairs (incl. Verbal Paired Associates): 2 3. Object-location associative learning task: 1 |
| visuoconstructional abilities | 3 | **1. Rey-Osterrieth Complex Figure test: 6** 2. Clock Drawing Test: 2 3: Line Orientation: 1 |
| logical memory | 1 | **Story recall** (incl. WMS Logical Memory) was used to assess logical memory. |
| subjective cognitive functioning | 4 | All measures were used by only one study: Memory Inventory for Chinese, Cognitive Failures Questionnaire, Cognitive Difficulties Scale, Perceived Deficits Questionnaire. |
| memory strategies | 5 | 1. **Multifactorial Memory Questionnaire** (full or Strategy subscale only): 5 All other measures were used by only one study. |
| everyday memory | 3 | All measures were used by only one study: Prospective Memory Test, Prospective and Retrospective Memory Questionnaire, Rivermead Behavioural Memory Test |
| inhibitory control | 2 | **1. Stroop Colour and Word test: 2** 2. Categorisation Working Memory Span: 1 |
| semantic memory | 3 | **1. Verbal Fluency Test: 2** All other measures were used by only one study. |
| non-memory cognition | 2 | **1. Trail Making Test: 2** All other measures were used by only one study (one study used 6 different outcome measures). |
| confidence in cognition and memory | 2 | All measures were used by only one study: bespoke Likert scale, Questionnaire of Confidence |
| everyday cognitive function | 1 | Both studies used the **Cognitive Failures Questionnaire**. |
| information processing | 2 | All measures were used by only one study: CANTAB Rapid Visual Information Processing, story recall. |
| meta-cognitive ability | 2 | All measures were used by only one study: Judgement of Learning, Meta Memory Questionnaire |
| perceived control over memory | 1 | Two studies used the **Memory Controllability Inventory.** |
| abstract thinking | 2 | One study measured abstract thinking using two subtests (Similarities and Matrix Reasoning) from the WAIS. |
| cognitive flexibility | 2 | One study measured cognitive flexibility using the number-letter switching subtest from the Delis-Kaplan Executive Function system and cognitive control tests from Lumosity. |
| cognitive/perceptual load | 1 | One study measured cognitive/perceptual load using the Timed Up and Go test combined with a cognitive test. |
| concentration | 1 | One study measured concentration using Digit Span. |
| declarative memory | 1 | One study measured declarative memory using story recall. |
| fluid intelligence | 1 | One study measured fluid intelligence using the Culture Fair Test Scale 3. |
| prospective memory | 1 | One study measured prospective memory using the Envelope Task. |
| spiritual wellbeing | 1 | One study measured spiritual wellbeing using the Spiritual Health and Life-Orientation Measure. |
| understanding of factors affecting memory | 1 | One study measured understanding of factors affecting memory using a bespoke questionnaire on meta-memory and memory-related habits and behaviours (e.g., stress, nutrition, activity). |
| orientation | 1 | One study measured orientation using the Supra-span of Corsi. |
| word-finding | 1 | One study measured word-finding using the Boston Naming Test. |
| Delivery of care outcomes | | |
| adherence and tolerability | 2 | 1. Completion (rate) of intervention-related tasks (e.g., taking capsules, attending therapy sessions): 12 2. Self-reported anxiety and fatigue during sessions: 1 |
| satisfaction with the intervention | 1 | All studies used bespoke satisfaction rating scales (incl. Visual Analog Scale): 4 |
| satisfaction with outcomes | 5 | All measures were used by only one study: bespoke satisfaction rating, Self-Assessment of Cognition Questionnaire, Satisfaction Questionnaire, Functional Activities Questionnaire, Cognitive Failures Questionnaire |
| acceptability and feasibility | 2 | All measures were used by only one study: bespoke questionnaire, Technology Acceptance Questionnaire |
| long-term maintenance of intervention | 1 | One study measured long-term maintenance of intervention using a participant diary. |
| willingness to continue with intervention | 1 | One study measured willingness to continue with intervention using a Visual Analog Scale. |
| Emotional functioning/wellbeing | | |
| mood | 16 | 1. Geriatric Depression Scale (15, 20, or 30-item): 5 2. Montgomery Asberg Depression Rating Scale, Depression Anxiety and Stress Scale (21-item), and Neuropsychiatric Inventory were all used by two studies. The remaining scales were all used by a single study and covered mood questionnaires and questionnaires specific to depression, anxiety, stress, and apathy. |
| psychological wellbeing | 4 | All measures were used by only one study: Multifactorial Memory Questionnaire Contentment subtest, General Well-Being Schedule, Geriatric Depression Scale (15-item), and Geriatric Anxiety Scale (20-item) |
| wellbeing | 3 | All measures were used by only one study: RAND SF-36 (full), Well-Being Scale, and a selection of subtests from the RAND SF-36 (Role-Emotional, Mental Health, Vitality, and Social Functioning) |
| apathy | 1 | Three studies used the **Apathy Evaluation Scale**. |
| self-esteem | 1 | Two studies used the **Rosenberg Self-Esteem Scale**. |
| stress | 1 | Two studies used the **Perceived Stress Scale**. |
| acceptance | 2 | One study used the Illness Cognition Questionnaire and one study used the Acceptance subtest only from the Illness Cognition Questionnaire. |
| helplessness | 1 | Two studies used the **Helplessness subtest from the Illness Cognition Questionnaire**. |
| mindfulness | 2 | All measures were used by only one study: Mindful Attention and Awareness Scale, Five-Facet Mindfulness Questionnaire |
| distress | 1 | One study reported measuring distress using the Geriatric Depression Scale (15-item). |
| coping strategies | 1 | One study reported measuring coping strategies using the Brief-COPE. |
| resilience to stress | 1 | One study reported measuring resilience to stress using the Connor-Davidson Resilience scale. |
| self-confidence | 1 | One study reported measuring self-confidence using a Visual Analog Scale. |
| self-efficacy | 1 | One study reported measuring self-efficacy using the Self-Efficacy for managing MCI scale. |
| Global quality of life | | |
| quality of life | 9 | 1. **Quality of Life-Alzheimer's Disease (QOL-AD)**: 8 2. RAND 36-SF: 3 All other measures were used by a single study. |
| health-related quality of life | 3 | Two measures were used twice (RAND 36-SF and EQ-5D-5L). One study used the Impact Rating Scale, modified from the Illness Intrusiveness Scale. |
| Physical functioning | | |
| physical activity levels | 8 | 1. **Step count**: 4 2. Minutes of exercise at moderate-vigorous intensity: 3 3. Two studies each reported minutes of exercise per week or used a Physical Activity Questionnaire. |
| balance | 9 | 1. **Timed Up and Go**: 4 2. Two studies each used the Functional Reach test and Berg Balance Scale.  All other measures were only used once. |
| physical functioning | 9 | 1. **ADL scale**: 2 All other measures were used only once but included balance measures, physical fitness tests, and gait speed. |
| walking | 3 | 1. **Walking speed (single-task walking)**: 6 One study also used walking speed and errors made during two dual-task walking conditions: working memory test and Stroop Carpet test. |
| functional mobility | 1 | All four studies used **Timed Up and Go** test. |
| falls | 5 | Two studies each used the Physiological Profile Assessment and number of falls. All other measures were used once. |
| gait | 3 | 1. **GAITRite Portable Walkway System**: 2 The GAITUp System and actigraphy were each used by a single study. |
| psycho-motor skills | 7 | All measures were used by a single study: TMT, Stroop Colour and Word, Digit Symbol Substitution Test, CogState Detection Test, percent primary submovement, movement jerk, movement time. |
| physical fitness | 6 | 1. Senior (Physical) Fitness Test: 2 All other measures were used by a single study. |
| reaction time | 4 | All measures were used by a single study: Stroop Colour and Word, Choice Reaction Time, Disjunctive Reaction Time, and reaction time during cognitive-motor tasks |
| confidence in balance | 1 | Activities-Specific Balance Confidence scale was used by a single study. |
| number of outdoor activities | 1 | Self-reported number of times someone went outdoors was used by a single study. |
| Physical functioning | | |
| instrumental activities of daily living | 13 | 1. **Instrumental Activities of Daily Living Scale**: 5 2. Bayer Activities of Daily Living Scale: 3 3. ADCS Mild Cognitive Impairment Activities of Daily Living Scale: 2 |
| functional ability |  | 1. **Instrumental Activities of Daily Living Scale**: 4 2. Activities of Daily Living Scale and Functional Activities Questionnaire were both used in 3 studies. 3. Function and Cognitive Assessment Test and Functional Rating Scale for Symptoms of Dementia were both used in 2 studies. |
| basic activities of daily living | 7 | 1. Activities of Daily Living Scale: 8 2. ADCS Mild Cognitive Impairment Activities of Daily Living Scale: 2 All other measures were used only once. |
| participation in activities | 2 | One study each used the Leisure Activity Questionnaire and US Health Interview Survey to measure participation in activities. |
| occupational performance | 1 | One study used the Canadian Occupational Performance Measure to measure occupational performance. |
| Social functioning | | |
| relationships with others | 2 | One study each used the Social Network Satisfaction Questionnaire and Lubben Social Network Scale to measure relationships with others. |
| conversational ability | 1 | One study used a biosensor device to measure conversation time. |
| satisfaction with relationships | 1 | One study used the Maudsley Marital Questionnaire Emotional Satisfaction subtest to meure satisfaction with relationships. |
| social participation | 1 | One study used an unnamed questionnaire published previously in the paper 'A study of the relationships among interpersonal intimacy, social participation, and mental health in elders'. |
| Personal circumstances | | |
| goal-oriented rehabilitation | 1 | One study measured goal-oriented rehabilitation using the Canadian Occupational Performance Measure. |
| Endocrine outcomes | | |
| insulin-like growth factor 1 | 1 | Three studies used **blood (plasma or serum) levels** to measure insulin-like growth factor 1. |
| cortisol | 1 | One study used blood plasma to measure cortisol. |
| cortisol awakening response | 1 | One study used serial saliva samples to measure cortisol awakening response. |
| General outcomes | | |
| body composition | 2 | Two studies used a body composition analyser and two studies recorded height and weight. |
| bespoke outcome related to intervention | 1 | Two studies reported bespoke 'behavioral effects'/'training progress'/'task performance' relating to a cognitive task completed during imaging but did not give any further information allowing for categorisation of the outcome. |
| Traditional Chinese medicine symptoms of dementia | 2 | One study used the Chinese Syndrome Differentiation Scale and one study used the Chinese Medicine Dementia Syndromes Scale. |
| physical health | 2 | One study used the Medical Health Questionnaire and one used the RAND SF-36. |
| deqi sensations | 1 | One study used a bespoke 10-point scale to capture deqi sensations (subjective sensations during acupuncture). |
| fibroblast growth factor-2 | 1 | One study used blood serum to measure fibroblast growth factor 2. |
| sensory functioning | 1 | One study used Sniffin' Sticks to measure sensory functioning. |
| sleep quality | 1 | One study used a Visual Analog Scale to measure sleep quality. |
| alcohol intake | 1 | One study used the Alcohol Use Disorders Identification Test to measure alcohol intake. |
| telomere length | 1 | One study used whole blood samples to measure telomere length. |
| Immune system outcomes | | |
| cytokines | 2 | 1. **Interleukins (IL-1B, IL-15, IL-8, IL-10, IL-6) in blood**: 4 2. Tumor necrosis factor alpha in blood: 3 |
| C-reactive protein | 1 | C-reactive protein was measured by two studies, both in **blood** samples. |
| platelet factor 4 | 1 | Platelet factor 4 was measured in one study in blood plasma. |
| peripheral blood monocytes | 1 | Peripheral blood monocytes was measured in one study in blood plasma. |
| phagocytic activity | 1 | Phagocytic activity was measured in one study in whole blood. |
| Metabolism and nutrition outcomes | | |
| cholesterol | 1 | Cholesterol was measured in **blood samples (serum and plasma)** and included specific measures of low-density lipoproteins, high-density lipoproteins, triglycerides, and total cholesterol. |
| insulin | 1 | Insulin was measured in blood (serum and plasma). |
| insulin sensitivity | 3 | 1. **Homeostasis model assessment:** 2 One study used an oral glucose tolerance test and one study used a hyperinsulinemic-euglycemic clamp technique. |
| haemoglobin A1c | 1 | Haemoglobin A1c was measured in blood (serum). |
| erythrocytes fatty acid membrane composition | 1 | Erythrocytes fatty acid membrane composition was measured in blood. |
| leptin | 1 | Leptin was measured in blood (serum). |
| adiposity | 1 | Adiposity was measured by calculating the percentage of body fat. |
| vitamin B metabolism | 1 | Vitamin B metabolism was measured in blood. |
| homocysteine | 1 | Homocysteine was measured in blood. |
| omega-3 intake | 1 | Omega-3 intake was measured using the omega-3 index in blood. |
| Musculoskeletal and connective tissue outcomes | | |
| muscular strength | 4 | Each outcome was used by only one study: z-score of one-rep max on various lifts, mean loads of 8 reps, handheld dynamometer, 30-second sit-stand test. |
| handgrip strength | 1 | All four studies used a **handheld dynamometer**. |
| muscle endurance (lower limbs) | 1 | One study used a 30-second sit-stand test. |
| Nervous system outcomes | | |
| brain volume or structure | 2 | 1. **Magnetic resonance imaging (MRI):** 15 2. Computerised Tomography: 1 |
| functional connectivity | 2 | **1. (Functional) Magnetic resonance imaging (MRI):** 10 2. Electromagnetic Tomography: 1 |
| resting state or spontaneous brain activity | 2 | **1. (Functional) Magnetic resonance imaging (MRI):** 9 2. Electroencephalography (EEG): 1 |
| brain activation during a cognitive task | 2 | **1. (Functional) Magnetic resonance imaging (MRI):** 9 2. (Functional) near-infrared spectroscopy: 1 |
| brain-derived neurotrophic factor | 1 | All studies used **blood (plasma or serum)** to measure brain-derived neurotrophic factor. |
| progression to dementia (incidence) | 5 | Each outcome measure was only used by a single study and included diagnostic criteria (DSM, NIA-AA) and standardised tests (MMSE, CDR, Dementia Rating Scale-2). |
| dementia severity | 2 | 1. **Clinical Dementia Rating (CDR):** 3 2. Clinical Dementia Rating-Sum of Boxes (CDR-SB): 1 |
| severity of symptoms | 3 | 1. Global Deterioration Scale: 2 2. Clinical Global Impression Scale / Clinician's Interview-Based Impression of Change Scale: 1 |
| brain metabolism | 1 | All three studies used Fluorodeoxyglucose Positron Emission Tomography **(FDG-PET).** |
| amyloid-beta burden | 3 | All measures were used by a single study: blood (plasma), cerebrospinal fluid, and amyloid PET scan. |
| brain function | 2 | All measures were used by a single study: functional MRI, EEG |
| brain perfusion and cerebrovascular health | 3 | 1. MRI: 2 2. Blood pressure / carotid intima-media thickness: 1 |
| EEG frequency | 2 | One study used relative spectral power and one study used resting-state EEG. |
| time to progression to dementia | 2 | Two studies used time to development of dementia using standardised criteria: one specified which criteria (NINCDS-ADRDA and DSM-IV-TR) and one did not. |
| EEG signatures | 1 | One study used event-related potentials on EEG. |
| tau burden | 1 | One study measured tau burden in cerebrospinal fluid. |
| Psychiatric outcomes | | |
| depression | 9 | 1. **Geriatric Depression Scale**: 23 2. Beck Depression Inventory / Hamilton Depression Scale: 4 3. Center for Epidemiological Studies-Depression Scale: 2 |
| anxiety | 6 | 1. **Goldberg Anxiety Scale:** 2 All other measures were only used once. |
| neuropsychiatric symptoms | 2 | 1. **Neuropsychiatric Inventory:** 4 2. Hospital Anxiety and Depression Scale: 1 |
| behavioural disturbance | 3 | 1. **Neuropsychiatric Inventory:** 2 2. State-Trait Anxiety Inventory and Geriatric Depression Scale were each used in a single study. |
| rumination | 1 | One study used the Ruminative Response Scale to measure rumination. |
| behavioural and psychological symptoms of dementia (BPSD) | 1 | One study used the Neuropsychiatric Inventory to measure BPSD. |
| Respiratory, thoracic, and mediastinal outcomes | | |
| cardiorespiratory fitness | 8 | 1. **VO2 max (or peak):** 5 2. Heart rate/blood pressure: 2 All other measures were used by a single study. |
| aerobic fitness | 5 | Each measure was used by a single study: stationary walk test, Rockport test, VO2, carbon dioxide production, maximal exercise on cycling ergometer |
| Vascular outcomes | | |
| cardiovascular health | 4 | All measures were used by a single study: carotid ultrasound, lipids, blood pressure, VO2 max |
| vascular endothelial growth factor | 1 | Two studies used **blood (serum)** to measure vascular endothelial growth factor. |
| blood pressure | 1 | Arterial blood pressure was used by a single study. |
| Societal/caregiver burden | | |
| caregiver burden | 2 | 1. **Zarit Scale of Caregiver Burden:** 4 2. Sense of Competence Questionnaire: 1 |

* Translations or cultural adaptations of a measure into other languages are included as a single outcome. For some well-known tasks such as auditory verbal learning tests (AVLT), verbal fluency, digit span, the exact outcome measurement tool used was often not specified so these have been included as a single outcome measure.
