# Supplementary material for: Outcomes reported in interventional studies for mild cognitive impairment: a scoping umbrella review to inform core outcome set development
Source: Alzheimers Dement. 2026 Sep 5;22(9):e71800. doi: 10.1002/alz.71800 (PMC13546403; doi:10.1002/alz.71800)
Supplement: Supplementary file 5 [file ALZ-22-e71800-s004.docx]

Supplementary Materials – Appendix B

Further information on review methodology

65 reviews were eligible at full-text screening. Due to individual studies frequently being included across multiple reviews, reviews were stratified according to intervention type: cognitive interventions, computerised cognitive training, exercise, dance, non-invasive brain stimulation, pharmacological, traditional Chinese herbal medicine (including acupuncture and tai chi), non-pharmacological, multidomain intervention, and unspecified.

We extracted data from all eligible articles within an intervention category with fewer than 5 reviews, i.e.:

- Computerised cognitive training (N = 3)
- Dance (N = 2)
- Pharmacological (N = 2)
- Non-invasive brain stimulation (N = 2)
- Unspecified (N = 1)

For categories with 5 or more reviews, we randomly selected 50% of reviews and extracted data from all eligible articles within them:

- Cognitive: 20 reviews were identified, so N=10 randomly selected for data extraction
- Exercise: 8 reviews were identified, so N=4 randomly selected for data extraction
- Traditional Chinese medicine: 13 reviews were identified, so N=7 randomly selected for data extraction
- Non-pharmacological: 7 reviews were identified, so N=4 randomly selected for data extraction
- Multidomain interventions: 7 reviews were identified, so N=4 randomly selected for data extraction

In total, data was extracted from eligible articles within 39 different reviews (39/65, 60% of eligible reviews at full-text screening). Details of the reviews and articles from which data were extracted are provided in the table below.

Summary of included review articles

| Review citation | | N (%) of articles included | Study identifiers for studies included from review | Unique studies added | Reason for studies excluded (N) | Top-line findings |
| --- | --- | --- | --- | --- | --- | --- |
| **Cognitive interventions** (N=10) | | | | | |  |
| 1. **Chandler** MJ, Parks AC, Marsiske M, Rotblatt LJ, Smith GE. Everyday Impact of Cognitive Interventions in Mild Cognitive Impairment: a Systematic Review and Meta-Analysis. Neuropsychol Rev. 2016 Sep;26(3):225-251. doi: 10.1007/s11065-016-9330-4. Epub 2016 Sep 8. PMID: 27632385; PMCID: PMC5048589. | | 23/30 (77%) | 13, 14, 16, 18, 20, 21, 22, 24, 25, 30, 32, 34, 35, 36, 40, 54, 55, 58, 61, 71, 72 | 2 | Study population (N=7) | Cognitive interventions had small effects on metacognition and activities of daily living, but not quality of life. More research is needed. |
| 2. **Chow** G, Gan JKE, Chan JKY, Wu XV, Klainin-Yobas P. Effectiveness of psychosocial interventions among older adults with mild cognitive impairment: a systematic review and meta-analysis. Aging Ment Health. 2021 Nov;25(11):1986-1997. doi: 10.1080/13607863.2020.1839861. Epub 2020 Nov 17. PMID: 33200623. | | 8/10 (80%) | 16, 22, 35, 41, 42, 43, 44, 45, | 2 | Study population (N=2) | Psychosocial interventions enhanced memory but not executive function, depression, or activities of daily living. Interventions with longer treatment durations and individual formats were more beneficial. |
| 3. **Gates** NJ, Sachdev PS, Fiatarone Singh MA, Valenzuela M. Cognitive and memory training in adults at risk of dementia: a systematic review. BMC Geriatr. 2011 Sep 25;11:55. doi: 10.1186/1471-2318-11-55. PMID: 21942932; PMCID: PMC3191477. | | 7/10 (70%) | 13, 14, 18, 34, 37, 40, 88, | 0 | Study population (N=3) | Cognitive interventions can produce moderate-to-large effects on memory, however the number of high-quality trials remains low. |
| 4. **Gómez-Soria** I, Peralta-Marrupe P, Calatayud-Sanz E, Latorre E. Efficacy of cognitive intervention programs in amnesic mild cognitive impairment: A systematic review. Arch Gerontol Geriatr. 2021 May-Jun;94:104332. doi: 10.1016/j.archger.2020.104332. Epub 2021 Jan 14. PMID: 33486120. | | 7/7 (100%) | 21, 26, 30, 67, 69, 70, 113 | 1 | n/a | Cognitive interventions improved cognitive scores on Mini-Mental State Examination in patients with amnestic MCI, but not other cognitive outcome tools (e.g., ADAS-Cog). Cognitive training and rehabilitation showed some domain-specific benefits for memory, language, and executive function. |
| 5. **Hong** YJ, Jang EH, Hwang J, Roh JH, Lee JH. The Efficacy of Cognitive Intervention Programs for Mild Cognitive Impairment: A Systematic Review. *Curr Alzheimer Res*. 2015;12(6):527-542. doi:10.2174/1567205012666150530201636 | | 30/32 (91%) | 1, 3, 4, 13, 14, 16, 18, 19, 20, 21, 22, 24, 25, 26, 27, 28, 29, 30, 31, 32, 33, 34, 35, 36, 37, 38, 39 | 3 | Study population (N=2) | Cognitive interventions improved outcomes related to trained tasks, in addition to some positive outcomes in neuroimaging and transfer effects. |
| 6. **Huckans** M, Hutson L, Twamley E, Jak A, Kaye J, Storzbach D. Efficacy of cognitive rehabilitation therapies for mild cognitive impairment (MCI) in older adults: working toward a theoretical model and evidence-based interventions. Neuropsychol Rev. 2013 Mar;23(1):63-80. doi: 10.1007/s11065-013-9230-9. Epub 2013 Mar 8. PMID: 23471631; PMCID: PMC3640648. | | 10/14 (71%) | 5, 6, 13, 19, 20, 22, 24, 30, 40, 63 | 1 | Study population (N=4) | Substantial heterogeneity in outcome measures and study design quality, though most evidence suggested cognitive rehabilitation helps targeted behaviours and global cognition. More evidence is needed across other important measures and on long-term outcomes. |
| 7. **Li** H, Li J, Li N, Li B, Wang P, Zhou T. Cognitive intervention for persons with mild cognitive impairment: A meta-analysis. *Ageing Res Rev*. 2011;10(2):285-296. doi:10.1016/j.arr.2010.11.003 | | 14/20 (70%) | 1, 2, 3, 4, 13, 14, 18, 19, 20, 34, 35, 37, 39, 40 | 0 | Study population (N=5)  Not available in English (N=1)* | Cognitive interventions improved language, anxiety, and functional ability and other cognitive domains and quality of life to a lesser extent. |
| 8. **Liang** JH, Shen WT, Li JY, Qu XY, Li J, Jia RX, Wang YQ, Wang S, Wu RK, Zhang HB, Hang L, Xu Y, Lin L. The optimal treatment for improving cognitive function in elder people with mild cognitive impairment incorporating Bayesian network meta-analysis and systematic review. Ageing Res Rev. 2019 May;51:85-96. doi: 10.1016/j.arr.2019.01.009. Epub 2019 Jan 22. PMID: 30682429. | | 11/13 (85%) | 18, 22, 24, 30, 32, 44, 46, 47, 48, 49 | 1 | Study population (N=2) | Cognitive stimulation has the strongest evidence in improving cognition in MCI patients. Cognitive training also had benefits, and cognitive rehabilitation had the least support. |
| 9. **Simon** SS, Yokomizo JE, Bottino CM. Cognitive intervention in amnestic Mild Cognitive Impairment: a systematic review. *Neurosci Biobehav Rev*. 2012;36(4):1163-1178. doi: 10.1016/j.neubiorev.2012.01.007 | | 19/20 (95%) | 1, 2, 4, 6, 7, 18, 19, 20, 21, 22, 35, 36, 37, 38, 39, 40, 59, 64, 65 | 3 | Study population (N=1) | Cognitive interventions helped with global cognition, specifically memory, of MCI patients in non-standardised cognitive measures and subjective measures, but findings in standardised neuropsychological tests were limited. |
| 10. **Sung** CM, Jen HJ, Liu D, et al. The effect of cognitive training on domains of attention in older adults with mild cognitive impairment and mild dementia: A meta-analysis of randomised controlled trials. *J Glob Health*. 2023;13:04078. Published 2023 Jun 30. doi:10.7189/jogh.13.04078 | | 12/17 (71%) | 16, 19, 25, 33, 54, 58, 74, 75, 76, 77, 78, 79 | 3 | Study population (N=5) | Cognitive training improved attention (overall, selective, and divided) and global cognitive function, but with relatively low effectiveness. |
| **Computer-based cognitive interventions** (N=3) | | | | | | |
| 11. **Ge** S, Zhu Z, Wu B, McConnell ES. Technology-based cognitive training and rehabilitation interventions for individuals with mild cognitive impairment: a systematic review. BMC Geriatr. 2018 Sep 15;18(1):213. doi: 10.1186/s12877-018-0893-1. PMID: 30219036; PMCID: PMC6139138. | | 19/26 (73%) | 18, 19, 23, 25, 27, 34, 46, 50, 54, 57, 58, 82, 83, 113, 140, 141, 142, 143, | 4 | Study population (N=7) | Technology-based cognitive training and rehabilitation have shown potential benefits for several cognitive domains but inconsistent methodologies limit conclusions. |
| 12. **Heo** S, Park J. Cognition-specific computerized cognitive training in older adults with mild cognitive impairment: a systematic review and meta-analysis. International Journal of Gerontology. 2021; 15(2):90-96. doi: 10.6890/IJGE.202104_15(2).00 | | 7/8 (88%) | 27, 47, 58, 81, 83, 112, 113 | 1 | Study population (N=1) | Cognition-specific computerised cognitive training was benefit for global cognition, memory, and working memory but not attention or executive function. Long-term trials are warranted. |
| 13. **Zhang** H, Huntley J, Bhome R, Holmes B, Cahill J, Gould RL, Wang H, Yu X, Howard R. Effect of computerised cognitive training on cognitive outcomes in mild cognitive impairment: a systematic review and meta-analysis. BMJ Open. 2019 Aug 18;9(8):e027062. doi: 10.1136/bmjopen-2018-027062. PMID: 31427316; PMCID: PMC6701629. | | 15/17 (88%) | 18, 23, 25, 27, 46, 47, 51, 54, 55, 58, 80, 81, 82, 83, 113, 51 |  | Study population (N=2) | Computerised cognitive training improves cognition in older adults with MCI, however outcome heterogeneity and interventions limits quality of evidence. |
|  | **Dance interventions** (N = 2) | | | | | |
|  | 14. **Chan** JSY, Wu J, Deng K, Yan JH. The effectiveness of dance interventions on cognition in patients with mild cognitive impairment: A meta-analysis of randomized controlled trials. Neurosci Biobehav Rev. 2020 Nov;118:80-88. doi: 10.1016/j.neubiorev.2020.07.017. Epub 2020 Jul 17. PMID: 32687886. | 4/5 (80%) | 100, 109, 110 | 0 | Study population (N=1) | Dance interventions improved global cognition, attention, immediate and delayed recall, and visuospatial ability in MCI patients, but there were few studies and high heterogeneity across some cognitive domains. |
|  | 15. **Yuan** Y, Li X, Liu W. Dance activity interventions targeting cognitive functioning in older adults with mild cognitive impairment: A meta-analysis. Front Psychol. 2022 Sep 27;13:966675. doi: 10.3389/fpsyg.2022.966675. PMID: 36237681; PMCID: PMC9553227. | 5/12 (42%)* | 100, 109, 110, 146 | 0 | Study population (N=2)  Not available in English (N=5)* | Dance interventions improved global cognition, visuospatial functioning, cognitive flexibility, attention, and balance in older adults with MCI, but more rigorous study designs are warranted. |
|  | **Exercise interventions** (N = 4) | | | | | |
|  | 16. **Huang** X, Zhao X, Cai Y, Wan Q. The cerebral changes induced by exercise interventions in people with mild cognitive impairment and Alzheimer's disease: A systematic review. Arch Gerontol Geriatr. 2022 Jan-Feb;98:104547. doi: 10.1016/j.archger.2021.104547. Epub 2021 Oct 3. PMID: 34634494. | 14/19 (74%) | 58, 91, 100, 127, 128, 129, 131, 132 | 3 | Study population (N=5) | Exercise interventions showed positive effects on multiple outcomes relating to brain volume, functional connectivity, and brain activity. |
|  | 17. **Loprinzi** PD, Blough J, Ryu S, Kang M. Experimental effects of exercise on memory function among mild cognitive impairment: systematic review and meta-analysis. Phys Sportsmed. 2019 Feb;47(1):21-26. doi: 10.1080/00913847.2018.1527647. Epub 2018 Oct 8. PMID: 30246596. | 9/11 | 6, 33, 56, 57, 58, 60, 62, 129 | 3 | Study population (N=2) | Exercise may help to improve memory function in MCI patients. |
|  | 18. **Song** D, Yu DSF, Li PWC, Lei Y. The effectiveness of physical exercise on cognitive and psychological outcomes in individuals with mild cognitive impairment: A systematic review and meta-analysis. Int J Nurs Stud. 2018 Mar;79:155-164. doi: 10.1016/j.ijnurstu.2018.01.002. Epub 2018 Jan 4. PMID: 29334638. | 10/14 (71%) | 5, 6, 33, 58, 61, 90, 91 | 1 | Study population (N=4) | Physical exercise and specifically aerobic exercise benefited global cognition in patients with MCI, but evidence on domain-specific cognitive outcomes and psychological outcomes remains unclear. |
|  | 19. **Wang** R, Zhang H, Li H, Ren H, Sun T, Xu L, Liu Y, Hou X. The influence of exercise interventions on cognitive functions in patients with amnestic mild cognitive impairment: A systematic review and meta-analysis. Front Public Health. 2022 Nov 15;10:1046841. doi: 10.3389/fpubh.2022.1046841. PMID: 36457329; PMCID: PMC9706097. | 10/10 (100%) | 33, 42, 91, 110, 127, 134, 135, 136 | 3 | n/a | Exercise enhanced global cognition and some specific cognitive domains (e.g., immediate and delayed recall, executive functioning) but not others (e.g., working memory, attention) in amnestic MCI patients. |
| **Multi-domain interventions** (N = 4) | | | | | | |
| 20. **Lipardo** DS, Aseron AMC, Kwan MM, Tsang WW. Effect of Exercise and Cognitive Training on Falls and Fall-Related Factors in Older Adults With Mild Cognitive Impairment: A Systematic Review. Arch Phys Med Rehabil. 2017 Oct;98(10):2079-2096. doi: 10.1016/j.apmr.2017.04.021. Epub 2017 May 26. PMID: 28554873. | | 15/17 (88%) | 5, 16, 19, 20, 33, 40, 54, 55, 58, 61, 62, 66 | 1 | Study population (N=2) | Exercise, alone or combined with cognitive training, improved outcomes relating to falls including gait speed, cognitive function, and balance but more research is needed on direct fall outcomes. |
| 21. **Kiper** P, Richard M, Stefanutti F, Pierson-Poinsignon R, Cacciante L, Perin C, Mazzucchelli M, Viganò B, Meroni R. Combined Motor and Cognitive Rehabilitation: The Impact on Motor Performance in Patients with Mild Cognitive Impairment. Systematic Review and Meta-Analysis. J Pers Med. 2022 Feb 14;12(2):276. doi: 10.3390/jpm12020276. PMID: 35207764; PMCID: PMC8874573. | | 7/9 (78%) | 49, 51, 85, 152, 153, | 2 | Study population (N=2) | Combined physical and cognitive training enhanced balance compared to physical training alone and enhanced mobility compared to no intervention. There was no evidence that combined physical and cognitive training improved gait speed or quality of life following sessions, but longer follow-up trials are needed. |
| 22. **Salzman** T, Sarquis-Adamson Y, Son S, Montero-Odasso M, Fraser S. Associations of Multidomain Interventions With Improvements in Cognition in Mild Cognitive Impairment: A Systematic Review and Meta-analysis. JAMA Netw Open. 2022 May 2;5(5):e226744. doi: 10.1001/jamanetworkopen.2022.6744. Erratum in: doi: 10.1001/jamanetworkopen.2025.0896. PMID: 35503222; PMCID: PMC9066287. | | 23/28 (82%) | 33, 49, 51, 61, 68, 73, 84, 86, 88, 93, 99, 103, 138, 172 | 6 | Study population (N=4)  Paper retracted (N=1) | In the short-term, multidomain interventions were associated with improvements in global cognition as well as specific cognitive domains (e.g., executive functioning, memory) compared to single interventions. |
| 23. **Yang** C, Moore A, Mpofu E, Dorstyn D, Li Q, Yin C. Effectiveness of Combined Cognitive and Physical Interventions to Enhance Functioning in Older Adults With Mild Cognitive Impairment: A Systematic Review of Randomized Controlled Trials. Gerontologist. 2020 Nov 23;60(8):633-642. doi: 10.1093/geront/gnz149. PMID: 31697831. | | 8/10 (80%) | 49, 51, 58, 61, 85, 86, 88, 89, | 1 | Study population (N=2) | There is preliminary evidence to support positive effects of multi-component interventions in cognitive-motor abilities, but strength of the evidence is limited. |
| **Non-invasive brain stimulation** (N = 2) | | | | | | |
| 24. **Jiang** L, Cui H, Zhang C, Cao X, Gu N, Zhu Y, Wang J, Yang Z, Li C. Repetitive Transcranial Magnetic Stimulation for Improving Cognitive Function in Patients With Mild Cognitive Impairment: A Systematic Review. Front Aging Neurosci. 2021 Jan 14;12:593000. doi: 10.3389/fnagi.2020.593000. PMID: 33519418; PMCID: PMC7842279. | | 2/9 (22%)* | 144, 145 | 0 | Not available in English (N=7)* | Repetitive transcranial magnetic stimulation may help to improve global cognition and memory, but not executive functioning or attention. |
| 25. **Zhang** X, Lan X, Chen C, Ren H, Guo Y. Effects of Repetitive Transcranial Magnetic Stimulation in Patients With Mild Cognitive Impairment: A Meta-Analysis of Randomized Controlled Trials. Front Hum Neurosci. 2021 Oct 26;15:723715. doi: 10.3389/fnhum.2021.723715. PMID: 34764859; PMCID: PMC8576192. | | 5/12 (42%) | 87, 144, 145, 149, 150, | 3 | Study population (N=2)  Not available in English (N=5)* | High-frequency transcranial magnetic stimulation and multiple sessions help improve cognitive symptoms in MCI. |
| **Non-pharmacological interventions** (N = 4) | | | | | | |
| 26. **Hahn** EA, Andel R. Nonpharmacological therapies for behavioral and cognitive symptoms of mild cognitive impairment. *J Aging Health*. 2011;23(8):1223-1245. doi:10.1177/0898264311422745 | | 10/11 (91%) | 1, 2, 3, 4, 5, 6, 7, 8, 9, 10 | 3 | Study population (N=1) | Exercise and diet interventions may help with non-memory cognitive domains (e.g., cognitive speed, executive functioning) but not memory, however conclusions are limited by insufficient evidence. |
| 27. **Teixeira** V, Gobbi LT, Corazza DI, Stella F, Costa JL, Gobbi S. Non-pharmacological interventions on cognitive functions in older people with mild cognitive impairment (MCI). Arch Gerontol Geriatr. 2012 Jan-Feb;54(1):175-80. doi: 10.1016/j.archger.2011.02.014. Epub 2011 Mar 12. PMID: 21397960. | | 5/7 (71%) | 13, 14, 34, 35, 37 | 0 | Study population (N=7) | Physical activity and cognitive exercise may improve memory and executive functioning in patients with MCI, but more controlled studies are warranted. |
| 28. **Wang** YQ, Jia RX, Liang JH, Li J, Qian S, Li JY, Xu Y. Effects of non-pharmacological therapies for people with mild cognitive impairment. A Bayesian network meta-analysis. Int J Geriatr Psychiatry. 2020 Jun;35(6):591-600. doi: 10.1002/gps.5289. Epub 2020 Mar 12. PMID: 32119152. | | 14/25 (56%)* | 21, 22, 30, 32, 33, 46, 51, 61, 67, 77, 109, 110, 113, 151 | 1 | Study population (N=4)  Not available in English (N=7)* | Cognitive stimulation appears to be the optimal non-pharmacological therapy. Non-pharmacological interventions appear beneficial for cognition in MCI, but more high quality randomised controlled trials are needed. |
| 29. **Xu** Z, Sun W, Zhang D, Chung VC, Wong SY. Comparative effectiveness of non-pharmacological interventions for depressive symptoms in mild cognitive impairment: systematic review with network meta-analysis. Aging Ment Health. 2022 Nov;26(11):2129-2135. doi: 10.1080/13607863.2021.1998356. Epub 2021 Nov 28. PMID: 34841997. | | 20/22 (91%) | 16, 42, 44, 45, 48, 61, 70, 80, 92, 94, 95, 96, 97, 98, 99, 100, 101, 102, 110, | 6 | Study population (N=2) | Physical and cognitive interventions were effective for improving depressive symptoms in MCI patients. |
| **Pharmacological** (N = 2) | | | | | |  |
| 30. **Diniz** BS, Pinto JA Jr, Gonzaga ML, Guimarães FM, Gattaz WF, Forlenza OV. To treat or not to treat? A meta-analysis of the use of cholinesterase inhibitors in mild cognitive impairment for delaying progression to Alzheimer's disease. Eur Arch Psychiatry Clin Neurosci. 2009 Jun;259(4):248-56. doi: 10.1007/s00406-008-0864-1. Epub 2009 Feb 17. PMID: 19224111. | | 3/3 (100%) | 10, 11, 12 | 0 | n/a | Cholinesterase inhibitors may reduce progression to Alzheimer’s disease/dementia but were associated with greater risk of study dropout in patients with MCI, compared to patients on placebo. |
| 31. **Zhang** X, Lian S, Zhang Y, Zhao Q. Efficacy and safety of donepezil for mild cognitive impairment: A systematic review and meta-analysis. Clin Neurol Neurosurg. 2022 Feb;213:107134. doi: 10.1016/j.clineuro.2022.107134. Epub 2022 Jan 19. PMID: 35078087. | | 7/17 (41%)* | 10, 104, 106, 115, 116, 117, 119 | 4 | Not available in English (N=9)* | Donepezil significantly improved scores on the Mini-Mental State Examination and Montreal Cognitive Assessment, but not ADAS-Cog scores and disease progression was not slowed. Adverse reactions included nausea, vomiting, and diarrhoea. |
| **Traditional Chinese Medicine** (N = 7) | | | | | | |
| 32. **Dong L,** May BH, Feng M, et al. Chinese Herbal Medicine for Mild Cognitive Impairment: A Systematic Review and Meta-Analysis of Cognitive Outcomes. *Phytother Res*. 2016;30(10):1592-1604. doi:10.1002/ptr.5679 | | 3/51 (6%)* | 52, 108, 114 | 1 | Not available in English (N=48)* | Chinese herbal medicine may improve cognitive outcomes but small sample sizes and methodological weaknesses mean results should be interpreted with caution. |
| 33. **Leow** Y, Rashid NLBA, Klainin-Yobas P, Zhang Z, Wu XV. Effectiveness of mindfulness-based interventions on mental, cognitive outcomes and neuroplastic changes in older adults with mild cognitive impairment: A systematic review and meta-analysis. J Adv Nurs. 2023 Dec;79(12):4489-4505. doi: 10.1111/jan.15720. Epub 2023 May 29. PMID: 37248564. | | 9/10 (90%) | 97, 75, 79, 98, 133, 147, 148 | 3 | Wrong publication type (N=1) | Mindfulness-based interventions may improve cortical thickness and connectivity in regions associated with memory and attention, but more research is needed. |
| 34. **Li** W, Wang Q, Du S, Pu Y, Xu G. Acupuncture for mild cognitive impairment in elderly people: Systematic review and meta-analyses. Medicine (Baltimore). 2020 Sep 25;99(39):e22365. doi: 10.1097/MD.0000000000022365. PMID: 32991455; PMCID: PMC7523831. | | 1/15 (7%)* | 17 | 1 | Not available in English (N=14)* | Acupuncture improved cognitive scores and activities of daily living, however more rigorous long-term studies are needed. |
| 35. **Li** X, Lai L, Lu L, Yan L, Deng K, Li Z, Xu N, Zhao J. Comparative efficacy of acupuncture-related techniques for mild cognitive impairment: A Bayesian network analysis. Front Neurol. 2022 Nov 15;13:942682. doi: 10.3389/fneur.2022.942682. PMID: 36457861; PMCID: PMC9706122. | | 4/27 (15%)* | 121, 137, 138, 139, | 2 | Not available in English (N=23)* | Acupuncture appeared to improve global cognition, however there was publication bias. |
| 36. **Wang** W, Diwu Y, Liu Q, Zhou Y, Sayed TI, Wang D, Gou Y. Chinese herbal medicine for mild cognitive impairment using mini-mental state examination: A systematic review and meta-analysis. Medicine (Baltimore). 2021 Sep 24;100(38):e27034. doi: 10.1097/MD.0000000000027034. PMID: 34559097; PMCID: PMC8462642. | | 4/21 (19%)* | 111, 114, 120, 130 | 3 | Not available in English (N=17)* | Chinese herbal medicine, alone or combined with other treatments, can improve MMSE scores. |
| 37. **Xia** R, Ren J, Wang M, Wan Y, Dai Y, Li X, Wu Z, Chen S. Effect of acupuncture on brain functional networks in patients with mild cognitive impairment: an activation likelihood estimation meta-analysis. Acupunct Med. 2023 Oct;41(5):259-267. doi: 10.1177/09645284221146199. Epub 2023 Feb 15. PMID: 36790017. | | 5/8 (63%) | 122, 123, 124, 125, 126 | 5 | Not available in English (N=3)* | There is an overall positive effect of acupuncture on brain functional networks in older adults with MCI. |
| 38. **Yin** Z, Li Y, Zhang X, Zia M, Chen Z, Zhao L Liang F. Moxibustion Ameliorates Cognitive Function in Older Adults with Mild Cognitive Impairment: A Systematic Review and Meta-Analysis of Randomized Controlled Trials. European Journal of Integrative Medicine. 2022 Aug; 53:102133. doi: 10.1016/j.eujim.2022.102133 | | 1/9 (11%)* | 154 | 1 | Not available in English (N=8)* | There were preliminary findings that moxibustion may help with global cognition, but there were no differences in memory. |
| **Unspecified** (N = 1) | |  |  |  |  |  |
| 39. **Fitzpatrick-Lewis** D, Warren R, Ali MU, Sherifali D, Raina P. Treatment for mild cognitive impairment: a systematic review and meta-analysis. *CMAJ Open*. 2015;3(4):E419-E427. Published 2015 Dec 1. doi:10.9778/cmajo.20150057 | | 13/17 (76%) | 6, 10, 11, 12, 24, 32, 33, 104, 105, 106, 107, 108 | 2 | Study population (N=4) | Cholinesterase inhibitors and studies using vitamins/supplements showed no significant effects, however there was a small cognitive benefit of uncertain clinical significance for patients with MCI following a behavioural intervention compared to controls |
| N.B. “Study identifiers for studies included from review” will not sum to “N (%) of articles included” where several reports of the same study have been collapsed. To demonstrate overlap across reviews, study IDs unique to only a single included review are underlined. | | | | | | |

**Study population exclusion criteria**

- Non-community dwelling
- Mixed cohort without results explicitly provided for participants with MCI (e.g., MCI and dementia, MCI and subjective cognitive impairment)
- Unclear description of MCI diagnosis or criteria used. Studies were required to use standardised criteria for MCI (e.g., Petersen, DSM) or give an adequate description of criteria which aligns with most criteria for MCI: subjective cognitive complaints, objective cognitive impairment upon testing, no dementia and/or clinically confirmed diagnosis.

**Access exclusion criteria**

- Unable to access gain access to article (through university or NHS accounts)
- Article not published or available in English* - these articles were subtracted from the denominator used to calculate “≥ 70% included studies to be eligible” requirement to reduce bias against interventions predominantly conducted in China.
- Unable to find the article online* - these articles were subtracted from the denominator used to calculate “≥ 70% included studies to be eligible” requirement to reduce bias against interventions predominantly conducted in China.
