# Supplementary material for: Outcomes reported in interventional studies for mild cognitive impairment: a scoping umbrella review to inform core outcome set development
Source: Alzheimers Dement. 2026 Sep 5;22(9):e71800. doi: 10.1002/alz.71800 (PMC13546403; doi:10.1002/alz.71800)
Supplement: Supplementary file 6 [file ALZ-22-e71800-s005.docx]

Supplementary Materials – Appendix C

Study characteristics for included primary articles

|  | Study ID | Included in N reviews | Country | Study design | Intervention type | Type of MCI | Diagnostic criteria | N | N male | N female | Mean age |
| --- | --- | --- | --- | --- | --- | --- | --- | --- | --- | --- | --- |
| 1 | Londos (2008) [1] | 4 | Sweden | Pilot | Cognitive: Goal-oriented memory training | Amnestic MCI | American Academy of Neurology | 15 | 7 | 8 | 61.0 |
| 2 | Clare (2009) [2] | 3 | UK | Case study | Cognitive: Goal-oriented CR | Amnestic MCI | Petersen | 1 | 0 | 1 | 77.0 |
| 3 | Joosten-Weyn Banningh (2008) [3] | 3 | Netherlands | Pretest-posttest | Cognitive: CBT and psychoeducation | MCI | Petersen | 23 | 10 | 13 | 68.7 |
| 4 | Greenaway (2008) [4] | 4 | USA | Pretest-posttest | Cognitive: behavioural rehabilitation | Amnestic MCI | Mayo | 20 | 14 | 6 | 78.2 |
| 5 | Baker (2010) [5] | 5 | USA | RCT | Exercise: high-intensity aerobic training | Amnestic MCI | Petersen | 33 | 16 | 17 | 70 |
| 6 | van Uffelen (2008) [6, 7] | 5 | Netherlands | RCT | Multi-domain: walking, vitamin B supplements | MCI | Petersen | 152 | 85 | 67 | Not stated |
| 7 | Lodgson (2009) [8] | 1 | USA | Feasibility | Exercise: with behavioural support for memory loss | MCI | Petersen | 37 | 8 | 29 | 81.9 |
| 8 | Yan (2006) [9] | 1 | USA | Pretest-posttest | Exercise: motor training | Amnestic MCI | Petersen | 29 | 16 | 13 | 73.9 |
| 9 | Chiu (2008) [10] | 1 | Taiwan | RCT | Diet/nutrition: omega-3 fatty acids | Amnestic MCI | Petersen | 23 | Not stated | Not stated | Not stated |
| 10 | Petersen (2005) [11] | 4 | 2 countries: USA, Canada | RCT | Multi-domain: vitamin E supplements, donepezil | Amnestic MCI | Petersen | 769 | 417 | 352 | 72.9 |
| 11 | Feldman (2007) [12] | 2 | 14 countries | RCT | Pharmacological: Rivastigmine | MCI | Generic | 1,018 | 532 | 486 | 70.5 |
| 12 | Winblad (2008) [13] | 2 | 2 countries: USA, Canada | RCT | Pharmacological: Galantamine | MCI | Winblad | 2,048 | 916 | 1132 | 69.9 |
| 13 | Rapp (2002) [14] | 7 | USA | RCT | Cognitive: Psychoeducation, relaxation, memory skills training, cognitive restructuring | MCI | Petersen | 19 | 8 | 11 | 75.1 |
| 14 | Belleville (2006) [15] | 6 | Canada | RCT | Cognitive: Episodic memory strategies | MCI | Petersen | 28 | 14 | 14 | 65.9 |
| 15 | Liao (2019) [16, 17] | 2 | Taiwan | RCT | Virtual reality: Physical and cognitive training | MCI | Generic | 34 | 11 | 23 | 74.4 |
| 16 | Vidovich (2015) [18] | 4 | Australia | RCT | Cognitive: Cognitive activity strategy training | MCI | European Consortium on Alzheimer’s Disease Working Group on MCI | 160 | 74 | 86 | Not stated |
| 17 | Zhang (2013) [19] | 1 | China | RCT | Traditional Chinese Medicine: Scalp electroacupuncture | MCI | Generic | 233 | 109 | 124 | 73.67 |
| 18 | Rozzini (2006) [20] | 7 | Italy | RCT | Multi-domain: Cholinesterase inhibitors with and without cognitive rehabilitation | MCI | Petersen | 59 | Not stated | Not stated | Not stated |
| 19 | Barnes (2009) [21] | 7 | USA | RCT | Computerised cognitive training: Auditory processing training | MCI | Winblad | 47 | 28 | 19 | 74.0 |
| 20 | Kinsella (2009) [22] | 6 | Australia | RCT | Cognitive: Psychoeducation, everyday memory strategies | Amnestic MCI | Petersen | 44 | 19 | 25 | 76.8 |
| 21 | Jean (2010) [23] | 5 | Canada | RCT | Cognitive: Errorless learning and spaced retrieval | Amnestic MCI | Petersen | 22 | 9 | 13 | 68.6 |
| 22 | Buschert (2011) [24-26] | 7 | Germany | Pilot | Cognitive: Cognitive training, behavioural activation | Amnestic MCI | Petersen | 24 | 12 | 12 | 71.2 |
| 23 | Rosen (2011) [27] | 3 | USA | RCT | Computerised cognitive training: Auditory processing training | MCI | Winblad | 12 | Not stated | Not stated | Not stated |
| 24 | Tsolaki (2010) [28] | 5 | Greece | RCT | Cognitive: CR | MCI | Petersen | 176 | 50 | 126 | 67.8 |
| 25 | Gagnon (2012) [29] | 5 | Canada | RCT | Computerised cognitive training: attentional control and meta-cognition | MCI | Petersen | 24 | Not stated | Not stated | 67.7 |
| 26 | Hampstead (2012) [30] | 2 | USA | RCT | Cognitive: Memory strategy training | Amnestic MCI | Petersen | 28 | Not stated | Not stated | 72.0 |
| 27 | Herrera (2012) [31] | 4 | France | RCT | Computerised cognitive training: Memory and attention training | Amnestic MCI | Petersen | 22 | 11 | 11 | 76.6 |
| 28 | Moro (2012) [32] | 1 | Italy | Crossover study | Cognitive: CS, metacognition, memory strategies | Amnestic MCI | Petersen | 30 | Not stated | Not stated | 70.9 |
| 29 | Carretti (2013) [33] | 1 | Italy | Pilot | Cognitive: Working memory training | Amnestic MCI | Petersen | 20 | 10 | 10 | 71.2 |
| 30 | Greenaway (2013) [34] | 6 | USA | RCT | Cognitive: Memory support strategies | Amnestic MCI | Petersen | 40 | Not stated | Not stated | 72.5 |
| 31 | Olchik (2013) [35] | 1 | Brazil | RCT | Cognitive: Memory training | MCI | Gauthier and Touchon | 47 | 9 | 38 | 70.1 |
| 32 | Rojas (2013) [36] | 5 | Argentina | RCT | Cognitive: Cognitive training | MCI | Petersen | 30 | 17 | 13 | 74.5 |
| 33 | Suzuki (2013) [37-40] | 8 | Japan | RCT | Exercise: Aerobic, endurance, strength, balance, and gait training | MCI | Petersen | 100 | 51 | 49 | 75.0 |
| 34 | Talassi (2007) [41] | 6 | Italy | Non-randomised controlled study | Cognitive: CR | MCI | Petersen | 37 | 17 | 20 | 76.2 |
| 35 | Kurz (2009) [42] | 5 | Germany | Non-randomised controlled study | Cognitive: CR | MCI | Winblad | 30 | 13 | 17 | 70.6 |
| 36 | Joosten-Weyn Banningh (2011) [43, 44] | 3 | Netherlands | Non-randomised controlled study | Cognitive: CBT | MCI | Petersen | 93 | 48 | 45 | 70.2 |
| 37 | Wenisch (2007) [45] | 4 | France | Non-randomised controlled study | Cognitive: CS | MCI | Petersen | 12 | 4 | 8 | 73.0 |
| 38 | Belleville (2011) [46] | 2 | Canada | Non-randomised controlled study | Cognitive: Memory training | Amnestic MCI | Petersen | 15 | 5 | 10 | 70 |
| 39 | Hampstead (2008) [47] | 3 | USA | Pilot | Cognitive: Explicit memory training | Amnestic MCI | Petersen | 8 | 5 | 3 | 75 |
| 40 | Troyer (2006) [48] | 6 | Canada | RCT | Cognitive: Psychoeducation, memory training | Amnestic MCI | Petersen | 48 | 22 | 26 | 75.4 |
| 41 | Rovner (2018) [49] | 1 | USA | RCT | Cognitive: Behavioural activation | Amnestic MCI | NIA-AA | 221 | 46 | 175 | 75.8 |
| 42 | Park (2019) [50] | 3 | South Korea | RCT | Multi-domain: Cognitive and physical training | Amnestic MCI | Petersen | 49 | 15 | 34 | 71.6 |
| 43 | Jirayucharoensak (2019) [51] | 1 | Thailand | RCT | Multi-domain: Game-based neurofeedback versus exergame-based training | Amnestic MCI | Petersen | 65 | 0 | 65 | Not stated |
| 44 | Jeong (2015) [52] | 3 | South Korea | RCT | Cognitive: Group-based versus home-based | Amnestic MCI | Petersen | 148 | 48 | 100 | 69.6 |
| 45 | Belleville (2018) [53] | 2 | Canada | RCT | Cognitive: Cognitive training, psychosocial intervention | Amnestic MCI | Petersen | 127 | 57 | 70 | 72.2 |
| 46 | Barban (2015) [54] | 4 | 4 countries: Italy, Greece, Norway, Spain | RCT | Cognitive: Computerised cognitive training, reminiscence therapy | MCI | Petersen | 106 | 56 | 50 | 73.6 |
| 47 | Han (2017) [55] | 3 | South Korea | RCT | Computerised cognitive training: Spaced retrieval memory training | MCI | Winblad | 43 | 23 | 20 | 74.0 |
| 48 | Langoni (2018) [56, 57] | 2 | Brazil | RCT | Exercise: Aerobic and strength training | MCI | Albert | 52 | 12 | 40 | 72.3 |
| 49 | Shimada (2018) [58] | 4 | Japan | RCT | Multi-domain: Cognitive and physical training | MCI | Petersen | 308 | 154 | 154 | 71.6 |
| 50 | Han (2014) [59] | 1 | South Korea | Feasibility | Computerised cognitive training: Spaced retrieval memory training | MCI | Winblad | 10 | 1 | 9 | 72.1 |
| 51 | Hagovská (2016) [60-63] | 4 | Slovak Republic | RCT | Multi-domain: Cognitive and balance training | MCI | ICD-9 | 80 | 41 | 39 | 67.07 |
| 52 | Zhang (2015) [64] | 1 | China | RCT | Traditional Chinese Medicine: Bushen Capsule | Amnestic MCI | Petersen | 44 | 23 | 21 | 63.7 |
| 53 | Jeong (2021) [63] | 1 | South Korea | RCT | Multi-domain: Cognitive and physical training | Amnestic MCI | Petersen | 26 | 8 | 18 | 71 |
| 54 | Finn (2011) [65] | 5 | Australia | RCT | Computerised cognitive training: Attention, processing speed, visual memory, cognitive control | Amnestic MCI | Winblad | 25 | 9 | 16 | 74.2 |
| 55 | Finn (2014) [66] | 3 | Australia | RCT | Computerised cognitive training: Repetition lag training | Amnestic MCI | Winblad | 31 | 9 | 22 | 75.2 |
| 56 | Sugano (2012) [67] | 1 | Japan | RCT | Multi-domain: Cognitive and aerobic training | MCI | Petersen | 44 | 26 | 18 | 72.8 |
| 57 | González-Palau (2014) [68] | 2 | Spain | Pretest-posttest | Multi-domain: Computerised cognitive and physical training | Amnestic MCI | Petersen | 11 | 2 | 9 | 74.6 |
| 58 | Fiatarone Singh (2014) [69-71] | 11 | Australia | RCT | Multi-domain: Progressive resistance and cognitive training | MCI | Petersen | 100 | 32 | 68 | 70.1 |
| 59 | Jean (2008) [72] | 1 | Canada | Case study | Cognitive: Errorless learning | Amnestic MCI | Petersen | 2 | 1 | 1 | 62.5 |
| 60 | Smith (2013) [73] | 1 | USA | Pretest-posttest | Exercise: Treadmill walking | MCI | Albert | 17 | 7 | 10 | 78.7 |
| 61 | Lam (2015) [74] | 7 | China | RCT | Multi-domain: Exercise, cognitive, and social interventions | MCI | Winblad | 555 | 121 | 434 | 75.4 |
| 62 | Law (2014) [75] | 2 | Hong Kong | RCT | Multi-domain: Functional tasks, cognitive training | MCI | Albert | 83 | 33 | 50 | 73.8 |
| 63 | Bayer-Carter (2011) [76] | 1 | USA | RCT | Diet/nutrition: High versus low saturated fat diet | Amnestic MCI | Mayo | 29 | 16 | 13 | 67.6 |
| 64 | Hampstead (2011) [77] | 1 | USA | RCT | Cognitive: Explicit memory training | Amnestic MCI | Petersen | 6 | 4 | 2 | 73.5 |
| 65 | Akhtar (2007) [78] | 1 | UK | Pretest-posttest | Cognitive: Errorless learning | MCI | Petersen | 16 | Not stated | Not stated | 78.2 |
| 66 | Hampstead (2012) [79] | 1 | USA | RCT | Cognitive: Memory training | Amnestic MCI | Petersen | 18 | Not stated | Not stated | 71.2 |
| 67 | Tarnanas (2014) [80] | 2 | Greece | RCT | Virtual reality: CS | Amnestic MCI | Petersen | 114 | 41 | 64 | 70.3 |
| 68 | Martin (2019) [81] | 1 | Australia | RCT | Multi-domain: Cognitive training, transcranial direct current stimulation | Amnestic MCI | Albert | 68 | 23 | 55 | 71.7 |
| 69 | Poptsi (2018) [82] | 1 | Greece | RCT | Cognitive: Computerised versus traditional delivery | Amnestic MCI | Petersen | 71 | 22 | 49 | 68.6 |
| 70 | Park (2019) [83] | 2 | South Korea | RCT | Cognitive: Home-based cognitive training | Amnestic MCI | NIA-AA | 50 | 22 | 28 | 70.2 |
| 71 | Brum (2009) [84] | 1 | Portugal | RCT | Cognitive: Memory, executive function, attention, language, visuospatial, orientation | MCI | Petersen | 34 | 9 | 25 | 73.7 |
| 72 | Hwang (2012) [85] | 1 | South Korea | RCT | Cognitive: Memory, executive function, attention, visuo-construction, orientation, abstract thinking | Amnestic MCI | Petersen | 11 | 3 | 7 | 65.3 |
| 73 | Kim (2020)[86] | 1 | South Korea | RCT | Multi-domain intervention: Electroacupuncture, computerised cognitive training | MCI | Petersen | 32 | 4 | 28 | 72.1 |
| 74 | Bernini (2020) [87] | 1 | Italy | RCT | Cognitive: Computerised versus traditional delivery | PD-MCI | Movement Disorder Society Task Force guidelines | 53 | 31 | 22 | 64.7 |
| 75 | Doshi (2021) [88] | 2 | Singapore | RCT | Multi-domain: mindfulness, CR | MCI | DSM-V | 76 | 33 | 43 | 67.0 |
| 76 | Duff (2021) [89] | 1 | USA | RCT | Computerised cognitive training: Auditory processing training | Amnestic MCI | Albert | 113 | 62 | 51 | 74.9 |
| 77 | Li (2019) [90] | 2 | China | RCT | Computerised cognitive training: Memory, processing speed, attention, visuospatial training | MCI | Albert | 147 | 72 | 75 | 70.4 |
| 78 | Pantoni (2017) [91] | 1 | Italy | RCT | Cognitive: Attention training | MCI | Winblad | 43 | 28 | 15 | 75.1 |
| 79 | Yu (2021) [92] | 2 | Singapore | RCT | Traditional Chinese Medicine: Mindfulness training | MCI | Petersen | 54 | 14 | 40 | 71.6 |
| 80 | Ciarmiello (2015) [93] | 2 | Italy | RCT | Cognitive: CS | Amnestic MCI | Petersen | 30 | 12 | 17 | 71.5 |
| 81 | Djabelkhir (2017) [94] | 2 | France | RCT | Computerised cognitive training: CS, cognitive engagement | MCI | Petersen | 20 | 7 | 13 | 76.7 |
| 82 | Hyer (2016) [95] | 2 | USA | RCT | Computerised cognitive training: Working memory training | MCI | Winblad | 68 | 32 | 36 | 75.1 |
| 83 | Lin (2016) [96] | 3 | USA | Pilot | Computerised cognitive training: Visual speed-of-processing training | Amnestic MCI | Albert | 21 | 11 | 10 | 73.0 |
| 84 | Li (2021) [97] | 1 | China | RCT | Exercise: Aerobic, strength, balance, coordination, sensitivity training | MCI | Petersen | 84 | 33 | 51 | NA |
| 85 | Combourieu Donnezan (2018) [98] | 3 | France | RCT | Multi-domain: Cognitive and physical training | MCI | Petersen | 69 | Not stated | Not stated | 76.8 |
| 86 | Fogarty (2016) [99] | 2 | Canada | RCT | Multi-domain: Tai Chi, memory training | MCI | Petersen | 40 | 19 | 21 | 72.0 |
| 87 | Turriziani (2012) [100] | 1 | Italy | Non-randomised controlled study | Non-invasive brain stimulation: Repetitive transcranial magnetic stimulation | MCI | Petersen | 8 | 6 | 2 | 66.4 |
| 88 | Köbe (2016) [101] | 2 | Germany | RCT | Multi-domain: omega-3 fatty acids, aerobic exercise, CS | MCI | Mayo | 22 | 14 | 8 | 70.0 |
| 89 | Maffei (2017) [102] | 1 | Italy | RCT | Multi-domain: Cognitive and physical training | MCI | European Consortium on Alzheimer’s Disease Working Group on MCI | 113 | 58 | 55 | 74.5 |
| 90 | Busse (2008) [103] | 1 | Brazil | RCT | Exercise: Resistance training | MCI | Generic | 31 | 8 | 23 | 72.2 |
| 91 | Hong (2018) [104] | 3 | South Korea | RCT | Exercise: Resistance training | MCI | Petersen | 22 | 6 | 16 | 76.8 |
| 92 | Giuli (2016) [105] | 1 | Italy | RCT | Cognitive: Psychoeducation, strategy training, cognitive training | MCI | Petersen | 97 | 36 | 61 | 76.3 |
| 93 | Zheng (2021) [106] | 1 | China | RCT | Traditional Chinese Medicine: Baduanjin | MCI | Petersen | 69 | 23 | 46 | 65.51 |
| 94 | Emsaki (2017) [107] | 1 | Iran | RCT | Cognitive: Memory specificity training | Amnestic MCI | Petersen | 20 | N/A | N/A | 63.4 |
| 95 | Park (2020) [108] | 1 | South Korea | RCT | Virtual reality: Korean culture-based intervention | Amnestic MCI | Petersen | 21 | 7 | 14 | 70.6 |
| 96 | Masika (2020) [109] | 1 | Tanzania | RCT | Arts-based: Visual art therapy | MCI | DSM-V | 39 | 5 | 34 | 72.7 |
| 97 | Larouche (2018) [110-112] | 2 | Canada | RCT | Multi-domain: Mindfulness, psychoeducation | Amnestic MCI | Generic | 48 | 26 | 22 | 71.0 |
| 98 | Klainin-Yobas (2019) [113] | 2 | Singapore | RCT | Traditional Chinese Medicine: Mindfulness | MCI | Petersen | 55 | 14 | 41 | 71.4 |
| 99 | Bae (2019) [114] | 2 | Japan | RCT | Multi-domain: Cognitive and physical training, social activities | MCI | Winblad | 83 | 42 | 41 | 76.0 |
| 100 | Zhu (2018) [115] | 5 | China | RCT | Exercise: Aerobic dance routine | MCI | NIA-AA | 60 | 24 | 36 | 69.6 |
| 101 | Mahendran (2018) [116] | 1 | Singapore | RCT | Multi-domain: Art therapy, reminiscence therapy | MCI | Petersen | 68 | 12 | 56 | 71.1 |
| 102 | Eyre (2016) [117] | 1 | USA | RCT | Mind-body exercise: Kundalini yoga | MCI | Generic | 79 | 27 | 52 | 68.7 |
| 103 | Thapa (2020) [118] | 1 | South Korea | RCT | Virtual reality: Cognitive training | MCI | Consortium to Establish a Registry for Alzheimer’s Disease Assessment Packet (CERAD) | 68 | 16 | 52 | 72.5 |
| 104 | Doody (2009) [119] | 2 | USA | RCT | Pharmacological: Donepezil | Amnestic MCI | Petersen | 778 | 402 | 376 | 70.0 |
| 105 | de Jager (2012) [120] | 1 | UK | RCT | Diet/nutrition: Vitamin B supplements | MCI | Petersen | 223 | 80 | 143 | 76.8 |
| 106 | Salloway (2004) [121] | 2 | USA | RCT | Pharmacological: Donepezil | MCI | Generic | 269 | 155 | 114 | 72.4 |
| 107 | Lee (2012) [122] | 1 | Malaysia | RCT | Diet/nutrition: Fish oil supplements | MCI | Generic | 35 | 8 | 27 | 64.9 |
| 108 | Yakoot (2013) [123] | 1 | Egypt | RCT | Diet/nutrition: Memo® supplements | MCI | DSM-IV-TR | 60 | 33 | 27 | 66.2 |
| 109 | Doi (2017) [124] | 4 | Japan | RCT | Multi-domain: dance, music, psychoeducation | MCI | Petersen | 201 | 97 | 104 | 76.0 |
| 110 | Lazarou (2017) [125] | 1 | Greece | RCT | Exercise: Ballroom dancing | Amnestic MCI | Petersen | 129 | 28 | 101 | 66.8 |
| 111 | Miao (2012) [126] | 1 | China | RCT | Traditional Chinese Medicine: Oral Chinese medicine | Amnestic MCI | Petersen | 117 | 54 | 63 | 64.4 |
| 112 | Park (2017) [127] | 1 | South Korea | RCT | Computerised cognitive training: Cognition-specific traning, non-specific computer training | Amnestic MCI | Petersen | 78 | 42 | 36 | 67.3 |
| 113 | Savulich (2017) [128] | 3 | UK | RCT | Computerised cognitive training: Memory training game | MCI due to AD | Albert | 42 | 25 | 17 | 76.1 |
| 114 | Zhang (2014) [129] | 1 | China | RCT | Traditional Chinese Medicine: Congrongyizhi capsule | Amnestic MCI | Petersen | 39 | 18 | 21 | 62.7 |
| 115 | Dubois (2015) [130] | 1 | France | RCT | Pharmacological: Donepezil | MCI | Clinical diagnosis | 216 | 103 | 113 | 73.9 |
| 116 | Montero-Odasso (2019) [131] | 1 | Canada | RCT | Pharmacological: Donepezil | MCI | Winblad | 45 | 25 | 20 | 75.0 |
| 117 | Reynolds (2011) [132] | 1 | USA | RCT | Pharmacological: Donepezil, antidepressants | MCI | National Alzheimer's Coordinating Center | 130 | 30 | 100 | Not stated |
| 118 | Devanand (2018) [133] | 1 | USA | RCT | Pharmacological: Donepezil, antidepressants | MCI | Alzheimer's Disease Neuroimaging Initiative | 79 | 41 | 38 | 68.9 |
| 119 | Özenli (2007) [134] | 1 | Turkey | RCT | Pharmacological: Donepezil | MCI | DSM-IV | 51 | 23 | 28 | Not stated |
| 120 | Zhang (2016) [135] | 1 | China | RCT | Traditional Chinese Medicine: Bushen capsule | Amnestic MCI | Petersen | 60 | 28 | 32 | 64.7 |
| 121 | Tan (2017) [136] | 1 | China | RCT | Traditional Chinese Medicine: Acupuncture | Amnestic MCI | Petersen | 32 | 16 | 16 | 65.9 |
| 122 | Jia (2015) [137] | 1 | China | RCT | Traditional Chinese Medicine: Acupuncture | MCI | Generic | 8 | 2 | 6 | 74.1 |
| 123 | Li (2020) [138] | 1 | China | RCT | Traditional Chinese Medicine: Acupuncture | Amnestic MCI | NIA-AA | 28 | 17 | 11 | 66.8 |
| 124 | Liu (2014) [139] | 1 | China | Non-randomised controlled study | Traditional Chinese Medicine: Acupuncture | Amnestic MCI | Petersen | 12 | 1 | 11 | 59.3 |
| 125 | Shan (2018) [140] | 1 | China | RCT | Traditional Chinese Medicine: Acupuncture | MCI | Petersen | 14 | 6 | 8 | 67.0 |
| 126 | Wang (2012) [141] | 1 | China | Non-randomised controlled study | Traditional Chinese Medicine: Acupuncture | MCI | Petersen | 8 | 3 | 5 | 66.4 |
| 127 | Thomas (2020) [142-145] | 2 | USA | RCT | Exercise: Aerobic training, stretching | Amnestic MCI | Petersen | 70 | 27 | 43 | 66.3 |
| 128 | Tsai (2019) [146] | 1 | Taiwan | RCT | Exercise: Aerobic, resistance training | Amnestic MCI | Gauthier and Touchon | 45 | 17 | 38 | 65.5 |
| 129 | ten Brinke (2015) [147] | 1 | Canada | RCT | Exercise: Aerobic, resistance training | MCI | Generic | 39 | 0 | 39 | 75.2 |
| 130 | Lin (2020) [148] | 1 | China | RCT | Multi-domain: Tiaobu Xinshen, donepezil | MCI due to AD | DSM-IV | 83 | 44 | 39 | 68.9 |
| 131 | Tao (2019) [149] | 2 | China | RCT | Multi-domain: Baduanjin (Traditional Chinese Medicine), brisk walking, psychoeducation | MCI | Petersen | 57 | 18 | 39 | 65.6 |
| 132 | Yogev-Seligmanna (2021) [150] | 1 | Israel | RCT | Exercise: Aerobic training, balance and toning | Amnestic MCI | Albert | 27 | NA | NA | 71.4 |
| 133 | Wells (2013) [151] | 1 | USA | RCT | Mind-body exercise: Mindfulness | MCI | Alzheimer's Disease Neuroimaging Initiative | 14 | 6 | 8 | 73.7 |
| 134 | Khanthong (2021) [152] | 1 | Thailand | RCT | Mind-body exercise: Ruesi Dadton (traditional Thai exercise) | Amnestic MCI | Petersen | 71 | 15 | 56 | 60.9 |
| 135 | Lü (2015) [153] | 1 | China | RCT | Exercise: Resistance training | MCI | Generic | 45 | 13 | 32 | 69.7 |
| 136 | Sungkarat (2018) [154, 155] | 1 | Thailand | RCT | Mind-body exercise: Tai Chi | Amnestic MCI | Petersen | 66 | 9 | 57 | 67.9 |
| 137 | Zhu (2012) [156] | 1 | China | RCT | Traditional Chinese Medicine: Acupuncture, moxibustion | MCI | China Expert Consensus on Prevention and Treatment of Cognition Disorder | 60 | 39 | 21 | 71.5 |
| 138 | Sun (2021) [157] | 2 | China | RCT | Multi-domain: Acupressure, cognitive training | Amnestic MCI | Petersen | 151 | 54 | 97 | 70.8 |
| 139 | Kim (2021) [158] | 1 | South Korea | RCT | Traditional Chinese Medicine: Acupuncture | MCI | Petersen | 32 | 5 | 27 | 66.1 |
| 140 | Styliadis (2015) [159, 160] | 1 | Greece | Non-randomised controlled study | Multi-domain: Computerised cognitive training, physical training | MCI | Petersen | 70 | 25 | 45 | 70.8 |
| 141 | Vermeij (2015)[161] | 1 | Netherlands | Pilot | Computerised cognitive training: Working memory training | Amnestic MCI | Petersen | 18 | 14 | 4 | 68.4 |
| 142 | Bahar-Fuchs (2017) [162] | 1 | Australia | RCT | Computerised cognitive training: Tailored adaptive cognitive training | MCI | NIA-AA | 8 | 4 | 4 | 74.8 |
| 143 | Hagovská (2017) [163] | 1 | Slovak Republic | RCT | Cognitive: Computerised versus traditional delivery | MCI | Albert | 60 | 29 | 31 | 68 |
| 144 | Marra (2015) [164] | 2 | Brazil | RCT | Non-invasive brain stimulation: Transcranial magnetic stimulation | MCI | Petersen | 34 | 12 | 22 | 65.2 |
| 145 | Cui (2019) [165] | 2 | China | RCT | Non-invasive brain stimulation: Transcranial magnetic stimulation | MCI due to AD | NIA-AA | 21 | 8 | 13 | 74.0 |
| 146 | Bisbe (2020) [166] | 3 | Spain | RCT | Exercise: Choreographed exercise, physical therapy | Amnestic MCI | Petersen | 31 | 16 | 15 | 74.9 |
| 147 | Marciniak (2020) [167] | 1 | Czech Republic | Pilot | Multi-domain: mindfulness-based stress reduction, cognitive training | MCI | Petersen | 20 | 7 | 13 | 74.0 |
| 148 | Fam (2019) [168] | 1 | Singapore | RCT | Mind-body exercise: Mindfulness | MCI | Petersen | 36 | 10 | 26 | 71.7 |
| 149 | Padala (2018) [169] | 1 | USA | RCT | Non-invasive brain stimulation: Transcranial magnetic stimulation | MCI | Petersen | 9 | 8 | 1 | 66.0 |
| 150 | Yuan (2021) [170] | 1 | China | RCT | Non-invasive brain stimulation: Transcranial magnetic stimulation | Amnestic MCI | Petersen | 24 | 11 | 13 | 64.9 |
| 151 | Siu (2018) [171] | 1 | Hong Kong | Pretest-posttest | Mind-body exercise: Tai Chi | MCI | Generic | 160 | 42 | 118 | NA |
| 152 | Lipardo (2020) [172] | 1 | Phillipines | RCT | Multi-domain: Physical training, cognitive training | MCI | Winblad | 92 | 19 | 73 | 69.0 |
| 153 | Cintoli (2021) [173] | 1 | Italy | RCT | Multi-domain: Physical training, cognitive training | MCI | European Consortium on Alzheimer’s Disease Working Group on MCI | 93 | 43 | 50 | 74.9 |
| 154 | Liu (2022) [174] | 1 | China | RCT | Traditional Chinese Medicine: Moxibustion | MCI | Petersen | 47 | 14 | 33 | 64.2 |

Abbreviations: CBT = cognitive-behavioural therapy; CR = cognitive rehabilitation; CS = cognitive stimulation; MCI = mild cognitive impairment; RCT = randomised controlled trial.

**References**

1.	Londos, E., et al., *Effects of a goal-oriented rehabilitation program in mild cognitive impairment: a pilot study.* Am J Alzheimers Dis Other Demen, 2008. **23**(2): p. 177-83.

2.	Clare, L., et al., *Goal-oriented cognitive rehabilitation for an individual with Mild Cognitive Impairment: behavioural and neuroimaging outcomes.* Neurocase, 2009. **15**(4): p. 318-31.

3.	Joosten-Weyn Banningh, L.W., et al., *A cognitive behavioural group therapy for patients diagnosed with mild cognitive impairment and their significant others: feasibility and preliminary results.* Clin Rehabil, 2008. **22**(8): p. 731-40.

4.	Greenaway, M.C., et al., *A behavioral rehabilitation intervention for amnestic mild cognitive impairment.* Am J Alzheimers Dis Other Demen, 2008. **23**(5): p. 451-61.

5.	Baker, L.D., et al., *Effects of aerobic exercise on mild cognitive impairment: a controlled trial.* Arch Neurol, 2010. **67**(1): p. 71-9.

6.	van Uffelen, J.G., et al., *Walking or vitamin B for cognition in older adults with mild cognitive impairment? A randomised controlled trial.* Br J Sports Med, 2008. **42**(5): p. 344-51.

7.	van Uffelen, J.G., et al., *The effect of walking and vitamin B supplementation on quality of life in community-dwelling adults with mild cognitive impairment: a randomized, controlled trial.* Qual Life Res, 2007. **16**(7): p. 1137-46.

8.	Logsdon, R.G., et al., *Making physical activity accessible to older adults with memory loss: a feasibility study.* Gerontologist, 2009. **49 Suppl 1**(Suppl 1): p. S94-9.

9.	Yan, J.H. and M.B. Dick, *Practice effects on motor control in healthy seniors and patients with mild cognitive impairment and Alzheimer's disease.* Neuropsychol Dev Cogn B Aging Neuropsychol Cogn, 2006. **13**(3-4): p. 385-410.

10.	Chiu, C.C., et al., *The effects of omega-3 fatty acids monotherapy in Alzheimer's disease and mild cognitive impairment: a preliminary randomized double-blind placebo-controlled study.* Prog Neuropsychopharmacol Biol Psychiatry, 2008. **32**(6): p. 1538-44.

11.	Petersen, R.C., et al., *Vitamin E and donepezil for the treatment of mild cognitive impairment.* N Engl J Med, 2005. **352**(23): p. 2379-88.

12.	Feldman, H.H., et al., *Effect of rivastigmine on delay to diagnosis of Alzheimer's disease from mild cognitive impairment: the InDDEx study.* Lancet Neurol, 2007. **6**(6): p. 501-12.

13.	Winblad, B., et al., *Safety and efficacy of galantamine in subjects with mild cognitive impairment.* Neurology, 2008. **70**(22): p. 2024-35.

14.	Rapp, S., G. Brenes, and A.P. Marsh, *Memory enhancement training for older adults with mild cognitive impairment: a preliminary study.* Aging Ment Health, 2002. **6**(1): p. 5-11.

15.	Belleville, S., et al., *Improvement of episodic memory in persons with mild cognitive impairment and healthy older adults: evidence from a cognitive intervention program.* Dement Geriatr Cogn Disord, 2006. **22**(5-6): p. 486-99.

16.	Liao, Y.Y., et al., *Using virtual reality-based training to improve cognitive function, instrumental activities of daily living and neural efficiency in older adults with mild cognitive impairment.* Eur J Phys Rehabil Med, 2020. **56**(1): p. 47-57.

17.	Liao, Y.Y., et al., *Effects of Virtual Reality-Based Physical and Cognitive Training on Executive Function and Dual-Task Gait Performance in Older Adults With Mild Cognitive Impairment: A Randomized Control Trial.* Front Aging Neurosci, 2019. **11**: p. 162.

18.	Vidovich, M.R., et al., *The PACE study: a randomized clinical trial of cognitive activity strategy training for older people with mild cognitive impairment.* Am J Geriatr Psychiatry, 2015. **23**(4): p. 360-372.

19.	Zhang, H., et al., *Clinical observation on effect of scalp electroacupuncture for mild cognitive impairment.* J Tradit Chin Med, 2013. **33**(1): p. 46-50.

20.	Rozzini, L., et al., *Efficacy of cognitive rehabilitation in patients with mild cognitive impairment treated with cholinesterase inhibitors.* Int J Geriatr Psychiatry, 2007. **22**(4): p. 356-60.

21.	Barnes, D.E., et al., *Computer-based cognitive training for mild cognitive impairment: results from a pilot randomized, controlled trial.* Alzheimer Dis Assoc Disord, 2009. **23**(3): p. 205-10.

22.	Kinsella, G.J., et al., *Early intervention for mild cognitive impairment: a randomised controlled trial.* J Neurol Neurosurg Psychiatry, 2009. **80**(7): p. 730-6.

23.	Jean, L., et al., *Efficacy of a cognitive training programme for mild cognitive impairment: results of a randomised controlled study.* Neuropsychol Rehabil, 2010. **20**(3): p. 377-405.

24.	Förster, S., et al., *Effects of a 6-month cognitive intervention program on brain metabolism in amnestic mild cognitive impairment and mild Alzheimer's disease.* J Alzheimers Dis, 2011. **25**(4): p. 695-706.

25.	Buschert, V.C., et al., *Effects of a newly developed cognitive intervention in amnestic mild cognitive impairment and mild Alzheimer's disease: a pilot study.* J Alzheimers Dis, 2011. **25**(4): p. 679-94.

26.	Buschert, V.C., et al., *Long-term observation of a multicomponent cognitive intervention in mild cognitive impairment.* J Clin Psychiatry, 2012. **73**(12): p. e1492-8.

27.	Rosen, A.C., et al., *Cognitive training changes hippocampal function in mild cognitive impairment: a pilot study.* J Alzheimers Dis, 2011. **26 Suppl 3**(Suppl 3): p. 349-57.

28.	Tsolaki, M., et al., *Effectiveness of nonpharmacological approaches in patients with mild cognitive impairment.* Neurodegener Dis, 2011. **8**(3): p. 138-45.

29.	Gagnon, L.G. and S. Belleville, *Training of attentional control in mild cognitive impairment with executive deficits: results from a double-blind randomised controlled study.* Neuropsychol Rehabil, 2012. **22**(6): p. 809-35.

30.	Hampstead, B.M., et al., *Mnemonic strategy training improves memory for object location associations in both healthy elderly and patients with amnestic mild cognitive impairment: a randomized, single-blind study.* Neuropsychology, 2012. **26**(3): p. 385-99.

31.	Herrera, C., et al., *Positive effects of computer-based cognitive training in adults with mild cognitive impairment.* Neuropsychologia, 2012. **50**(8): p. 1871-81.

32.	Moro, V., et al., *Cognitive stimulation in a-MCI: an experimental study.* Am J Alzheimers Dis Other Demen, 2012. **27**(2): p. 121-30.

33.	Carretti, B., et al., *Benefits of training working memory in amnestic mild cognitive impairment: specific and transfer effects.* Int Psychogeriatr, 2013. **25**(4): p. 617-26.

34.	Greenaway, M.C., N.L. Duncan, and G.E. Smith, *The memory support system for mild cognitive impairment: randomized trial of a cognitive rehabilitation intervention.* Int J Geriatr Psychiatry, 2013. **28**(4): p. 402-9.

35.	Olchik, M.R., et al., *Memory training (MT) in mild cognitive impairment (MCI) generates change in cognitive performance.* Arch Gerontol Geriatr, 2013. **56**(3): p. 442-7.

36.	Rojas, G.J., et al., *Efficacy of a cognitive intervention program in patients with mild cognitive impairment.* Int Psychogeriatr, 2013. **25**(5): p. 825-31.

37.	Suzuki, T., et al., *A randomized controlled trial of multicomponent exercise in older adults with mild cognitive impairment.* PLoS One, 2013. **8**(4): p. e61483.

38.	Doi, T., et al., *Effects of multicomponent exercise on spatial-temporal gait parameters among the elderly with amnestic mild cognitive impairment (aMCI): preliminary results from a randomized controlled trial (RCT).* Arch Gerontol Geriatr, 2013. **56**(1): p. 104-8.

39.	Makizako, H., et al., *Does a multicomponent exercise program improve dual-task performance in amnestic mild cognitive impairment? A randomized controlled trial.* Aging Clin Exp Res, 2012. **24**(6): p. 640-6.

40.	Suzuki, T., et al., *Effects of multicomponent exercise on cognitive function in older adults with amnestic mild cognitive impairment: a randomized controlled trial.* BMC Neurol, 2012. **12**: p. 128.

41.	Talassi, E., et al., *Effectiveness of a cognitive rehabilitation program in mild dementia (MD) and mild cognitive impairment (MCI): a case control study.* Arch Gerontol Geriatr, 2007. **44 Suppl 1**: p. 391-9.

42.	Kurz, A., et al., *Cognitive rehabilitation in patients with mild cognitive impairment.* Int J Geriatr Psychiatry, 2009. **24**(2): p. 163-8.

43.	Joosten-Weyn Banningh, L.W., et al., *Group therapy for patients with mild cognitive impairment and their significant others: results of a waiting-list controlled trial.* Gerontology, 2011. **57**(5): p. 444-54.

44.	Joosten-Weyn Banningh, L.W., et al., *Long-term effects of group therapy for patients with mild cognitive impairment and their significant others: a 6- to 8-month follow-up study.* Dementia (London), 2013. **12**(1): p. 81-91.

45.	Wenisch, E., et al., *Cognitive stimulation intervention for elders with mild cognitive impairment compared with normal aged subjects: preliminary results.* Aging Clin Exp Res, 2007. **19**(4): p. 316-22.

46.	Belleville, S., et al., *Training-related brain plasticity in subjects at risk of developing Alzheimer's disease.* Brain, 2011. **134**(Pt 6): p. 1623-34.

47.	Hampstead, B.M., et al., *Explicit memory training leads to improved memory for face-name pairs in patients with mild cognitive impairment: results of a pilot investigation.* J Int Neuropsychol Soc, 2008. **14**(5): p. 883-9.

48.	Troyer, A.K., et al., *Changing everyday memory behaviour in amnestic mild cognitive impairment: a randomised controlled trial.* Neuropsychol Rehabil, 2008. **18**(1): p. 65-88.

49.	Rovner, B.W., et al., *Preventing Cognitive Decline in Black Individuals With Mild Cognitive Impairment: A Randomized Clinical Trial.* JAMA Neurol, 2018. **75**(12): p. 1487-1493.

50.	Park, H., et al., *Combined Intervention of Physical Activity, Aerobic Exercise, and Cognitive Exercise Intervention to Prevent Cognitive Decline for Patients with Mild Cognitive Impairment: A Randomized Controlled Clinical Study.* J Clin Med, 2019. **8**(7).

51.	Jirayucharoensak, S., et al., *A game-based neurofeedback training system to enhance cognitive performance in healthy elderly subjects and in patients with amnestic mild cognitive impairment.* Clin Interv Aging, 2019. **14**: p. 347-360.

52.	Jeong, J.H., et al., *Group- and Home-Based Cognitive Intervention for Patients with Mild Cognitive Impairment: A Randomized Controlled Trial.* Psychother Psychosom, 2016. **85**(4): p. 198-207.

53.	Belleville, S., et al., *MEMO+: Efficacy, Durability and Effect of Cognitive Training and Psychosocial Intervention in Individuals with Mild Cognitive Impairment.* J Am Geriatr Soc, 2018. **66**(4): p. 655-663.

54.	Barban, F., et al., *Protecting cognition from aging and Alzheimer's disease: a computerized cognitive training combined with reminiscence therapy.* Int J Geriatr Psychiatry, 2016. **31**(4): p. 340-8.

55.	Han, J.W., et al., *Efficacy of the Ubiquitous Spaced Retrieval-based Memory Advancement and Rehabilitation Training (USMART) program among patients with mild cognitive impairment: a randomized controlled crossover trial.* Alzheimers Res Ther, 2017. **9**(1): p. 39.

56.	Langoni, C.D.S., et al., *The effect of group exercises on balance, mobility, and depressive symptoms in older adults with mild cognitive impairment: a randomized controlled trial.* Clin Rehabil, 2019. **33**(3): p. 439-449.

57.	Langoni, C.D.S., et al., *Effect of Exercise on Cognition, Conditioning, Muscle Endurance, and Balance in Older Adults With Mild Cognitive Impairment: A Randomized Controlled Trial.* J Geriatr Phys Ther, 2019. **42**(2): p. E15-e22.

58.	Shimada, H., et al., *Effects of Combined Physical and Cognitive Exercises on Cognition and Mobility in Patients With Mild Cognitive Impairment: A Randomized Clinical Trial.* J Am Med Dir Assoc, 2018. **19**(7): p. 584-591.

59.	Han, J.W., et al., *Development of the ubiquitous spaced retrieval-based memory advancement and rehabilitation training program.* Psychiatry Investig, 2014. **11**(1): p. 52-8.

60.	Hagovska, M. and I. Nagyova, *The transfer of skills from cognitive and physical training to activities of daily living: a randomised controlled study.* Eur J Ageing, 2017. **14**(2): p. 133-142.

61.	Hagovská, M. and Z. Olekszyová, *Impact of the combination of cognitive and balance training on gait, fear and risk of falling and quality of life in seniors with mild cognitive impairment.* Geriatr Gerontol Int, 2016. **16**(9): p. 1043-50.

62.	Hagovská, M., P. Takáč, and O. Dzvoník, *Effect of a combining cognitive and balanced training on the cognitive, postural and functional status of seniors with a mild cognitive deficit in a randomized, controlled trial.* Eur J Phys Rehabil Med, 2016. **52**(1): p. 101-9.

63.	Hagovská, M. and Z. Olekszyová, *Relationships between balance control and cognitive functions, gait speed, and activities of daily living.* Z Gerontol Geriatr, 2016. **49**(5): p. 379-85.

64.	Zhang, J., et al., *The Effects of Bushen Capsule on Episodic Memory in Amnestic Mild Cognitive Impairment Patients: A Pilot Placebo Controlled fMRI Study.* J Alzheimers Dis, 2015. **46**(3): p. 665-76.

65.	Finn, M. and S. McDonald, *Computerised Cognitive Training for Older Persons With Mild Cognitive Impairment: A Pilot Study Using a Randomised Controlled Trial Design.* Brain Impairment, 2011. **12**(3): p. 187-199.

66.	Finn, M. and S. McDonald, *Repetition-lag training to improve recollection memory in older people with amnestic mild cognitive impairment. A randomized controlled trial.* Neuropsychol Dev Cogn B Aging Neuropsychol Cogn, 2015. **22**(2): p. 244-58.

67.	Sugano, K., et al., *Effect of cognitive and aerobic training intervention on older adults with mild or no cognitive impairment: a derivative study of the nakajima project.* Dement Geriatr Cogn Dis Extra, 2012. **2**(1): p. 69-80.

68.	González-Palau, F., et al., *The effects of a computer-based cognitive and physical training program in a healthy and mildly cognitive impaired aging sample.* Aging Ment Health, 2014. **18**(7): p. 838-46.

69.	Mavros, Y., et al., *Mediation of Cognitive Function Improvements by Strength Gains After Resistance Training in Older Adults with Mild Cognitive Impairment: Outcomes of the Study of Mental and Resistance Training.* J Am Geriatr Soc, 2017. **65**(3): p. 550-559.

70.	Fiatarone Singh, M.A., et al., *The Study of Mental and Resistance Training (SMART) study—resistance training and/or cognitive training in mild cognitive impairment: a randomized, double-blind, double-sham controlled trial.* J Am Med Dir Assoc, 2014. **15**(12): p. 873-80.

71.	Suo, C., et al., *Therapeutically relevant structural and functional mechanisms triggered by physical and cognitive exercise.* Mol Psychiatry, 2016. **21**(11): p. 1633-1642.

72.	Jean, L., et al., *Towards a cognitive stimulation program using an errorless learning paradigm in amnestic mild cognitive impairment.* Neuropsychiatr Dis Treat, 2007. **3**(6): p. 975-85.

73.	Smith, J.C., et al., *Semantic memory functional MRI and cognitive function after exercise intervention in mild cognitive impairment.* J Alzheimers Dis, 2013. **37**(1): p. 197-215.

74.	Lam, L.C., et al., *Would older adults with mild cognitive impairment adhere to and benefit from a structured lifestyle activity intervention to enhance cognition?: a cluster randomized controlled trial.* PLoS One, 2015. **10**(3): p. e0118173.

75.	Law, L.L., et al., *Effects of functional tasks exercise on older adults with cognitive impairment at risk of Alzheimer's disease: a randomised controlled trial.* Age Ageing, 2014. **43**(6): p. 813-20.

76.	Bayer-Carter, J.L., et al., *Diet intervention and cerebrospinal fluid biomarkers in amnestic mild cognitive impairment.* Arch Neurol, 2011. **68**(6): p. 743-52.

77.	Hampstead, B.M., et al., *Activation and effective connectivity changes following explicit-memory training for face-name pairs in patients with mild cognitive impairment: a pilot study.* Neurorehabil Neural Repair, 2011. **25**(3): p. 210-22.

78.	Akhtar, S., C.J. Moulin, and P.C. Bowie, *Are people with mild cognitive impairment aware of the benefits of errorless learning?* Neuropsychol Rehabil, 2006. **16**(3): p. 329-46.

79.	Hampstead, B.M., et al., *Mnemonic strategy training partially restores hippocampal activity in patients with mild cognitive impairment.* Hippocampus, 2012. **22**(8): p. 1652-8.

80.	Tarnanas, I., A. Tsolakis, and M. Tsolaki, *Assessing Virtual Reality Environments as Cognitive Stimulation Method for Patients with MCI*, in *Technologies of Inclusive Well-Being: Serious Games, Alternative Realities, and Play Therapy*, A.L. Brooks, S. Brahnam, and L.C. Jain, Editors. 2014, Springer Berlin Heidelberg: Berlin, Heidelberg. p. 39-74.

81.	Martin, D.M., et al., *A Pilot Double-Blind Randomized Controlled Trial of Cognitive Training Combined with Transcranial Direct Current Stimulation for Amnestic Mild Cognitive Impairment.* J Alzheimers Dis, 2019. **71**(2): p. 503-512.

82.	Poptsi, E., et al., *A Comparative Single-Blind Randomized Controlled Trial With Language Training in People With Mild Cognitive Impairment.* Am J Alzheimers Dis Other Demen, 2019. **34**(3): p. 176-187.

83.	Park, J., et al., *Effect of 12-week home-based cognitive training on cognitive function and brain metabolism in patients with amnestic mild cognitive impairment.* Clin Interv Aging, 2019. **14**: p. 1167-1175.

84.	Brum, P.S., O.V. Forlenza, and M.S. Yassuda, *Cognitive training in older adults with Mild Cognitive Impairment: Impact on cognitive and functional performance.* Dement Neuropsychol, 2009. **3**(2): p. 124-131.

85.	Hwang, H.R., et al., *The effect of cognitive training in patients with mild cognitive impairment and early Alzheimer's disease: a preliminary study.* J Clin Neurol, 2012. **8**(3): p. 190-7.

86.	Kim, J.H., et al., *Cognitive Improvement Effects of Electroacupuncture Combined with Computer-Based Cognitive Rehabilitation in Patients with Mild Cognitive Impairment: A Randomized Controlled Trial.* Brain Sci, 2020. **10**(12).

87.	Bernini, S., et al., *A double-blind randomized controlled trial of the efficacy of cognitive training delivered using two different methods in mild cognitive impairment in Parkinson's disease: preliminary report of benefits associated with the use of a computerized tool.* Aging Clin Exp Res, 2021. **33**(6): p. 1567-1575.

88.	Doshi, K., et al., *Mindfulness-Based Training Does Not Improve Neuropsychological Outcomes in Mild Cognitive Impairment More Than Spontaneous Reversion Rates: A Randomized Controlled Trial.* J Alzheimers Dis, 2021. **84**(1): p. 449-458.

89.	Duff, K., et al., *Computerized Cognitive Training in Amnestic Mild Cognitive Impairment: A Randomized Clinical Trial.* J Geriatr Psychiatry Neurol, 2022. **35**(3): p. 400-409.

90.	Li, B.Y., et al., *Computerized cognitive training for Chinese mild cognitive impairment patients: A neuropsychological and fMRI study.* Neuroimage Clin, 2019. **22**: p. 101691.

91.	Pantoni, L., et al., *Effect of Attention Training in Mild Cognitive Impairment Patients with Subcortical Vascular Changes: The RehAtt Study.* J Alzheimers Dis, 2017. **60**(2): p. 615-624.

92.	Yu, J., et al., *Mindfulness intervention for mild cognitive impairment led to attention-related improvements and neuroplastic changes: Results from a 9-month randomized control trial.* J Psychiatr Res, 2021. **135**: p. 203-211.

93.	Ciarmiello, A., et al., *FDG-PET in the Evaluation of Brain Metabolic Changes Induced by Cognitive Stimulation in aMCI Subjects.* Curr Radiopharm, 2015. **8**(1): p. 69-75.

94.	Djabelkhir, L., et al., *Computerized cognitive stimulation and engagement programs in older adults with mild cognitive impairment: comparing feasibility, acceptability, and cognitive and psychosocial effects.* Clin Interv Aging, 2017. **12**: p. 1967-1975.

95.	Hyer, L., et al., *Cognitive Training Program to Improve Working Memory in Older Adults with MCI.* Clin Gerontol, 2016. **39**(5): p. 410-427.

96.	Lin, F., et al., *Cognitive and Neural Effects of Vision-Based Speed-of-Processing Training in Older Adults with Amnestic Mild Cognitive Impairment: A Pilot Study.* J Am Geriatr Soc, 2016. **64**(6): p. 1293-8.

97.	Li, L., et al., *Multi-component exercise training improves the physical and cognitive function of the elderly with mild cognitive impairment: a six-month randomized controlled trial.* Annals of Palliative Medicine, 2021. **10**(8): p. 8919-8929.

98.	Combourieu Donnezan, L., et al., *Effects of simultaneous aerobic and cognitive training on executive functions, cardiovascular fitness and functional abilities in older adults with mild cognitive impairment.* Mental Health and Physical Activity, 2018. **15**: p. 78-87.

99.	Fogarty, J.N., et al., *Taoist Tai Chi® and Memory Intervention for Individuals with Mild Cognitive Impairment.* J Aging Phys Act, 2016. **24**(2): p. 169-80.

100.	Turriziani, P., et al., *Enhancing memory performance with rTMS in healthy subjects and individuals with Mild Cognitive Impairment: the role of the right dorsolateral prefrontal cortex.* Front Hum Neurosci, 2012. **6**: p. 62.

101.	Köbe, T., et al., *Combined omega-3 fatty acids, aerobic exercise and cognitive stimulation prevents decline in gray matter volume of the frontal, parietal and cingulate cortex in patients with mild cognitive impairment.* Neuroimage, 2016. **131**: p. 226-38.

102.	*Randomized trial on the effects of a combined physical/cognitive training in aged MCI subjects: the Train the Brain study.* Sci Rep, 2017. **7**: p. 39471.

103.	Busse, A., et al., *Effects of resistance training exercise on cognitive performance in elderly individuals with memory impairment: Results of a controlled trial.* Einstein, 2008. **6**.

104.	Hong, S.G., J.H. Kim, and T.W. Jun, *Effects of 12-Week Resistance Exercise on Electroencephalogram Patterns and Cognitive Function in the Elderly With Mild Cognitive Impairment: A Randomized Controlled Trial.* Clin J Sport Med, 2018. **28**(6): p. 500-508.

105.	Giuli, C., et al., *The Effects of Cognitive Training for Elderly: Results from My Mind Project.* Rejuvenation Res, 2016. **19**(6): p. 485-494.

106.	Zheng, G., et al., *Traditional Chinese Mind-Body Exercise Baduanjin Modulate Gray Matter and Cognitive Function in Older Adults with Mild Cognitive Impairment: A Brain Imaging Study.* Brain Plast, 2021. **7**(2): p. 131-142.

107.	Emsaki, G., et al., *Memory specificity training can improve working and prospective memory in amnestic mild cognitive impairment.* Dement Neuropsychol, 2017. **11**(3): p. 255-261.

108.	Park, J.H., et al., *Feasibility and Tolerability of a Culture-Based Virtual Reality (VR) Training Program in Patients with Mild Cognitive Impairment: A Randomized Controlled Pilot Study.* Int J Environ Res Public Health, 2020. **17**(9).

109.	Masika, G.M., D.S.F. Yu, and P.W.C. Li, *Can Visual Art Therapy Be Implemented With Illiterate Older Adults With Mild Cognitive Impairment? A Pilot Mixed-Method Randomized Controlled Trial.* J Geriatr Psychiatry Neurol, 2021. **34**(1): p. 76-86.

110.	Larouche, E., C. Hudon, and S. Goulet, *Mindfulness mechanisms and psychological effects for aMCI patients: A comparison with psychoeducation.* Complement Ther Clin Pract, 2019. **34**: p. 93-104.

111.	Larouche, E., C. Hudon, and S. Goulet, *Mindfulness mechanisms and psychological effects for aMCI patients: A comparison with psychoeducation.* Complementary Therapies in Clinical Practice, 2019. **34**: p. 93-104.

112.	Larouche, E., C. Hudon, and S. Goulet, *Investigating a Mindfulness-Based Intervention as an Attentional Network Training to Improve Cognition in Older Adults with Amnestic Mild Cognitive Impairment: A Randomized-controlled Trial.* Neuropsychologie clinique et appliquée, 2018. **2**.

113.	Klainin-Yobas, P., et al., *The effects of mindfulness and health education programs on the emotional state and cognitive function of elderly individuals with mild cognitive impairment: A randomized controlled trial.* J Clin Neurosci, 2019. **68**: p. 211-217.

114.	Bae, S., et al., *The effect of a multicomponent intervention to promote community activity on cognitive function in older adults with mild cognitive impairment: A randomized controlled trial.* Complement Ther Med, 2019. **42**: p. 164-169.

115.	Zhu, Y., et al., *Effects of a specially designed aerobic dance routine on mild cognitive impairment.* Clin Interv Aging, 2018. **13**: p. 1691-1700.

116.	Mahendran, R., et al., *Art therapy is associated with sustained improvement in cognitive function in the elderly with mild neurocognitive disorder: findings from a pilot randomized controlled trial for art therapy and music reminiscence activity versus usual care.* Trials, 2018. **19**(1): p. 615.

117.	Eyre, H.A., et al., *A randomized controlled trial of Kundalini yoga in mild cognitive impairment.* Int Psychogeriatr, 2017. **29**(4): p. 557-567.

118.	Thapa, N., et al., *The Effect of a Virtual Reality-Based Intervention Program on Cognition in Older Adults with Mild Cognitive Impairment: A Randomized Control Trial.* J Clin Med, 2020. **9**(5).

119.	Doody, R.S., et al., *Donepezil treatment of patients with MCI: a 48-week randomized, placebo-controlled trial.* Neurology, 2009. **72**(18): p. 1555-61.

120.	de Jager, C.A., et al., *Cognitive and clinical outcomes of homocysteine-lowering B-vitamin treatment in mild cognitive impairment: a randomized controlled trial.* Int J Geriatr Psychiatry, 2012. **27**(6): p. 592-600.

121.	Salloway, S., et al., *Efficacy of donepezil in mild cognitive impairment: a randomized placebo-controlled trial.* Neurology, 2004. **63**(4): p. 651-7.

122.	Lee, L.K., et al., *Docosahexaenoic acid-concentrated fish oil supplementation in subjects with mild cognitive impairment (MCI): a 12-month randomised, double-blind, placebo-controlled trial.* Psychopharmacology (Berl), 2013. **225**(3): p. 605-12.

123.	Yakoot, M., A. Salem, and S. Helmy, *Effect of Memo®, a natural formula combination, on Mini-Mental State Examination scores in patients with mild cognitive impairment.* Clin Interv Aging, 2013. **8**: p. 975-81.

124.	Doi, T., et al., *Effects of Cognitive Leisure Activity on Cognition in Mild Cognitive Impairment: Results of a Randomized Controlled Trial.* J Am Med Dir Assoc, 2017. **18**(8): p. 686-691.

125.	Lazarou, I., et al., *International Ballroom Dancing Against Neurodegeneration: A Randomized Controlled Trial in Greek Community-Dwelling Elders With Mild Cognitive impairment.* Am J Alzheimers Dis Other Demen, 2017. **32**(8): p. 489-499.

126.	Miao, Y.C., et al., *Effects of Chinese medicine for tonifying the kidney and resolving phlegm and blood stasis in treating patients with amnestic mild cognitive impairment: a randomized, double-blind and parallel-controlled trial.* Zhong Xi Yi Jie He Xue Bao, 2012. **10**(4): p. 390-7.

127.	Park, J.H. and J.H. Park, *Does cognition-specific computer training have better clinical outcomes than non-specific computer training? A single-blind, randomized controlled trial.* Clin Rehabil, 2018. **32**(2): p. 213-222.

128.	Savulich, G., et al., *Cognitive Training Using a Novel Memory Game on an iPad in Patients with Amnestic Mild Cognitive Impairment (aMCI).* Int J Neuropsychopharmacol, 2017. **20**(8): p. 624-633.

129.	Zhang, J., et al., *The effects of CCRC on cognition and brain activity in aMCI patients: a pilot placebo controlled BOLD fMRI study.* Curr Alzheimer Res, 2014. **11**(5): p. 484-93.

130.	Dubois, B., et al., *Donepezil decreases annual rate of hippocampal atrophy in suspected prodromal Alzheimer's disease.* Alzheimers Dement, 2015. **11**(9): p. 1041-9.

131.	Montero-Odasso, M., et al., *Donepezil for gait and falls in mild cognitive impairment: a randomized controlled trial.* Eur J Neurol, 2019. **26**(4): p. 651-659.

132.	Reynolds, C.F., 3rd, et al., *Maintenance treatment of depression in old age: a randomized, double-blind, placebo-controlled evaluation of the efficacy and safety of donepezil combined with antidepressant pharmacotherapy.* Arch Gen Psychiatry, 2011. **68**(1): p. 51-60.

133.	Devanand, D.P., et al., *Donepezil Treatment in Patients With Depression and Cognitive Impairment on Stable Antidepressant Treatment: A Randomized Controlled Trial.* Am J Geriatr Psychiatry, 2018. **26**(10): p. 1050-1060.

134.	Özenli, Y., D. Yağcı, and S. Karaca, *Efficacy of donepezil on cognitive functions in mild cognitive impairment.* Psychiatry and Clinical Psychopharmacology, 2007. **17**(2): p. 62-67.

135.	Zhang, J., et al., *A Two-Year Treatment of Amnestic Mild Cognitive Impairment using a Compound Chinese Medicine: A Placebo Controlled Randomized Trial.* Scientific Reports, 2016. **6**(1): p. 28982.

136.	Tan, T.T., et al., *Modulatory effects of acupuncture on brain networks in mild cognitive impairment patients.* Neural Regen Res, 2017. **12**(2): p. 250-258.

137.	Jia, B., et al., *The Effects of Acupuncture at Real or Sham Acupoints on the Intrinsic Brain Activity in Mild Cognitive Impairment Patients.* Evid Based Complement Alternat Med, 2015. **2015**: p. 529675.

138.	Li, H., et al., *The Long-Term Effects of Acupuncture on Hippocampal Functional Connectivity in aMCI with Hippocampal Atrophy: A Randomized Longitudinal fMRI Study.* Neural Plasticity, 2020. **2020**(1): p. 6389368.

139.	Liu, Z., et al., *Exploring the Patterns of Acupuncture on Mild Cognitive Impairment Patients Using Regional Homogeneity.* PLOS ONE, 2014. **9**(6): p. e99335.

140.	Shan, Y., et al., *Neuronal Specificity of Acupuncture in Alzheimer's Disease and Mild Cognitive Impairment Patients: A Functional MRI Study.* Evid Based Complement Alternat Med, 2018. **2018**: p. 7619197.

141.	Wang, Z., et al., *Effect of acupuncture in mild cognitive impairment and Alzheimer disease: a functional MRI study.* PLoS One, 2012. **7**(8): p. e42730.

142.	Tarumi, T., et al., *Exercise Training in Amnestic Mild Cognitive Impairment: A One-Year Randomized Controlled Trial.* J Alzheimers Dis, 2019. **71**(2): p. 421-433.

143.	Tarumi, T., et al., *Cerebral White Matter Integrity in Amnestic Mild Cognitive Impairment: A 1-Year Randomized Controlled Trial of Aerobic Exercise Training.* J Alzheimers Dis, 2020. **73**(2): p. 489-501.

144.	Tomoto, T., et al., *One-Year Aerobic Exercise Reduced Carotid Arterial Stiffness and Increased Cerebral Blood Flow in Amnestic Mild Cognitive Impairment.* J Alzheimers Dis, 2021. **80**(2): p. 841-853.

145.	Thomas, B.P., et al., *Brain Perfusion Change in Patients with Mild Cognitive Impairment After 12 Months of Aerobic Exercise Training.* J Alzheimers Dis, 2020. **75**(2): p. 617-631.

146.	Tsai, C.L., et al., *Distinctive Effects of Aerobic and Resistance Exercise Modes on Neurocognitive and Biochemical Changes in Individuals with Mild Cognitive Impairment.* Curr Alzheimer Res, 2019. **16**(4): p. 316-332.

147.	ten Brinke, L.F., et al., *Aerobic exercise increases hippocampal volume in older women with probable mild cognitive impairment: a 6-month randomised controlled trial.* Br J Sports Med, 2015. **49**(4): p. 248-54.

148.	Lin, Z.Y., et al., *Tiaobu Xinshen Recipe () Improved Mild Cognitive Impairment of Alzheimer's Disease Patients with Xin (Heart) and Shen (Kidney) Deficiency.* Chin J Integr Med, 2020. **26**(1): p. 54-58.

149.	Tao, J., et al., *Mind-body exercise improves cognitive function and modulates the function and structure of the hippocampus and anterior cingulate cortex in patients with mild cognitive impairment.* Neuroimage Clin, 2019. **23**: p. 101834.

150.	Yogev-Seligmann, G., et al., *Neurocognitive Plasticity Is Associated with Cardiorespiratory Fitness Following Physical Exercise in Older Adults with Amnestic Mild Cognitive Impairment.* J Alzheimers Dis, 2021. **81**(1): p. 91-112.

151.	Wells, R.E., et al., *Meditation's impact on default mode network and hippocampus in mild cognitive impairment: a pilot study.* Neurosci Lett, 2013. **556**: p. 15-9.

152.	Khanthong, P., et al., *Traditional Thai exercise (Ruesi Dadton) for improving motor and cognitive functions in mild cognitive impairment: a randomized controlled trial.* J Exerc Rehabil, 2021. **17**(5): p. 331-338.

153.	Lü, J., et al., *Effects of momentum-based dumbbell training on cognitive function in older adults with mild cognitive impairment: a pilot randomized controlled trial.* Clin Interv Aging, 2016. **11**: p. 9-16.

154.	Sungkarat, S., et al., *Tai Chi Improves Cognition and Plasma BDNF in Older Adults With Mild Cognitive Impairment: A Randomized Controlled Trial.* Neurorehabil Neural Repair, 2018. **32**(2): p. 142-149.

155.	Sungkarat, S., et al., *Effects of Tai Chi on Cognition and Fall Risk in Older Adults with Mild Cognitive Impairment: A Randomized Controlled Trial.* J Am Geriatr Soc, 2017. **65**(4): p. 721-727.

156.	Zhu, C.-f., et al., *Influence of acupuncture-moxibustion treatment on cognition disorder.* Journal of Acupuncture and Tuina Science, 2012. **10**(4): p. 218-222.

157.	Sun, J., et al., *Acupressure and Cognitive Training Can Improve Cognitive Functions of Older Adults With Mild Cognitive Impairment: A Randomized Controlled Trial.* Front Psychol, 2021. **12**: p. 726083.

158.	Kim, J.-H., et al., *Factors contributing to cognitive improvement effects of acupuncture in patients with mild cognitive impairment: a pilot randomized controlled trial.* Trials, 2021. **22**(1): p. 341.

159.	Styliadis, C., et al., *Neuroplastic Effects of Combined Computerized Physical and Cognitive Training in Elderly Individuals at Risk for Dementia: An eLORETA Controlled Study on Resting States.* Neural Plasticity, 2015. **2015**(1): p. 172192.

160.	Klados, M.A., et al., *Beta-Band Functional Connectivity is Reorganized in Mild Cognitive Impairment after Combined Computerized Physical and Cognitive Training.* Front Neurosci, 2016. **10**: p. 55.

161.	Vermeij, A., et al., *Transfer and maintenance effects of online working-memory training in normal ageing and mild cognitive impairment.* Neuropsychol Rehabil, 2016. **26**(5-6): p. 783-809.

162.	Bahar-Fuchs, A., et al., *Tailored and Adaptive Computerized Cognitive Training in Older Adults at Risk for Dementia: A Randomized Controlled Trial.* J Alzheimers Dis, 2017. **60**(3): p. 889-911.

163.	Hagovská, M., O. Dzvoník, and Z. Olekszyová, *Comparison of Two Cognitive Training Programs With Effects on Functional Activities and Quality of Life.* Res Gerontol Nurs, 2017. **10**(4): p. 172-180.

164.	Drumond Marra, H.L., et al., *Transcranial Magnetic Stimulation to Address Mild Cognitive Impairment in the Elderly: A Randomized Controlled Study.* Behav Neurol, 2015. **2015**: p. 287843.

165.	Cui, H., et al., *Repetitive Transcranial Magnetic Stimulation Induced Hypoconnectivity Within the Default Mode Network Yields Cognitive Improvements in Amnestic Mild Cognitive Impairment: A Randomized Controlled Study.* J Alzheimers Dis, 2019. **69**(4): p. 1137-1151.

166.	Bisbe, M., et al., *Comparative Cognitive Effects of Choreographed Exercise and Multimodal Physical Therapy in Older Adults with Amnestic Mild Cognitive Impairment: Randomized Clinical Trial.* J Alzheimers Dis, 2020. **73**(2): p. 769-783.

167.	Marciniak, R., et al., *The Effect of Mindfulness-Based Stress Reduction (MBSR) on Depression, Cognition, and Immunity in Mild Cognitive Impairment: A Pilot Feasibility Study.* Clin Interv Aging, 2020. **15**: p. 1365-1381.

168.	Fam, J., et al., *Mindfulness practice alters brain connectivity in community-living elders with mild cognitive impairment.* Psychiatry Clin Neurosci, 2020. **74**(4): p. 257-262.

169.	Padala, P.R., et al., *Repetitive transcranial magnetic stimulation for apathy in mild cognitive impairment: A double-blind, randomized, sham-controlled, cross-over pilot study.* Psychiatry Res, 2018. **261**: p. 312-318.

170.	Yuan, L.Q., et al., *Neuroimaging mechanisms of high-frequency repetitive transcranial magnetic stimulation for treatment of amnestic mild cognitive impairment: a double-blind randomized sham-controlled trial.* Neural Regen Res, 2021. **16**(4): p. 707-713.

171.	Siu, M.-y. and D.T.F. Lee, *Effects of tai chi on cognition and instrumental activities of daily living in community dwelling older people with mild cognitive impairment.* BMC Geriatrics, 2018. **18**(1): p. 37.

172.	Lipardo, D.S. and W.W. Tsang, *Effects of combined physical and cognitive training on fall prevention and risk reduction in older persons with mild cognitive impairment: a randomized controlled study.* Clin Rehabil, 2020. **34**(6): p. 773-782.

173.	Cintoli, S., et al., *Effects of combined training on neuropsychiatric symptoms and quality of life in patients with cognitive decline.* Aging Clin Exp Res, 2021. **33**(5): p. 1249-1257.

174.	Liu, C., et al., *Altered functional connectivity density in mild cognitive impairment with moxibustion treatment: A resting-state fMRI study.* Brain Res, 2022. **1775**: p. 147732.
